# Ensemble structure of the N-terminal domain (1–267) of FUS in a biomolecular condensate

**DOI:** 10.1016/j.bpj.2024.01.023

**Published:** 2024-01-26

**Authors:** Laura Esteban-Hofer, Leonidas Emmanouilidis, Maxim Yulikov, Frédéric H.-T. Allain, Gunnar Jeschke

**Affiliations:** 1 ETH Zurich, Department of Chemistry and Applied Biosciences, Zurich, Switzerland; 2 ETH Zurich, Department of Biology, Zurich, Switzerland

## Abstract

Solutions of some proteins phase separate into a condensed state of high protein concentration and a dispersed state of low concentration. Such behavior is observed in living cells for a number of RNA-binding proteins that feature intrinsically disordered domains. It is relevant for cell function via the formation of membraneless organelles and transcriptional condensates. On a basic level, the process can be studied in vitro on protein domains that are necessary and sufficient for liquid-liquid phase separation (LLPS). We have performed distance distribution measurements by electron paramagnetic resonance for 13 sections in an N-terminal domain (NTD) construct of the protein fused in sarcoma (FUS), consisting of the QGSY-rich domain and the RGG1 domain, in the denatured, dispersed, and condensed state. Using 10 distance distribution restraints for ensemble modeling and three such restraints for model validation, we have found that FUS NTD behaves as a random-coil polymer under good-solvent conditions in both the dispersed and condensed state. Conformation distribution in the biomolecular condensate is virtually indistinguishable from the one in an unrestrained ensemble, with the latter one being based on only residue-specific Ramachandran angle distributions. Over its whole length, FUS NTD is slightly more compact in the condensed than in the dispersed state, which is in line with the theory for random coils in good solvent proposed by de Gennes, Daoud, and Jannink. The estimated concentration in the condensate exceeds the overlap concentration resulting from this theory. The QGSY-rich domain is slightly more extended, slightly more hydrated, and has slightly higher propensity for LLPS than the RGG1 domain. Our results support previous suggestions that LLPS of FUS is driven by multiple transient nonspecific hydrogen bonding and $\pi -sp^{2}$ interactions.

## Significance

Liquid-liquid phase separation (LLPS) underlies the formation of membraneless organelles under normal and stress conditions. The protein fused in sarcoma (FUS) is involved in the formation of stress granules, whose aberrant aging is related to amyotrophic lateral sclerosis and frontotemporal dementia. By deriving an ensemble model for the N-terminal domain (NTD), which undergoes LLPS in vitro, we obtain insight into the polymer solvation regime and chain extension of FUS NTD in a biomolecular condensate. Our results rationalize the slight compaction of FUS NTD upon LLPS and contribute to the understanding of the thermodynamics of biomolecular condensates.

## Introduction

Persistent as well as transient membraneless organelles are biomolecular condensates that arise from liquid-liquid phase separation (LLPS) (1,2,3,4). Their formation and integrity depend on multi-valent interactions between proteins and RNA (1,5), with charge-charge, charge-$sp^{2}$, and *π*-*π* interactions playing an important role (6). Often, intrinsically disordered regions (IDRs) of proteins are necessary or sufficient for driving LLPS (6,7,8). The importance of IDRs and of LLPS for the formation of biomolecular condensates establishes a strong link between polymer physics and biophysics (9). Biomolecular condensates, such as stress granules in cells, are not in a thermodynamic equilibrium state and may thus age. Such aging can lead to fibril formation of prion-like IDRs and to solidification (10), which is related to neurodegenerative diseases (11,12).

LLPS can be recapitulated in vitro through the formation of liquid droplets (13). Such experiments provide access to the basic polymer physics that underlies the formation of biomolecular condensates (14), although they may not necessarily capture the rich structure of inhomogeneous membraneless organelles (11). Although structural characterization of liquid droplets and their components have substantially improved our understanding of biomolecular condensates during the past decade, many questions remain open (15). In particular, it is difficult to determine the ensemble structure of proteins in biomolecular condensates, since the major techniques for structure determination, such as X-ray crystallography and cryoelectron microscopy, are not applicable for the condensed phase. NMR spectroscopy is applicable but does not usually provide a sufficient number of restraints. Here, we apply distance distribution measurements in the nanometer range by the electron paramagnetic resonance (EPR) technique double electron-electron resonance (DEER), which has recently been demonstrated to be applicable to liquid droplets (16).

**Figure 1:**
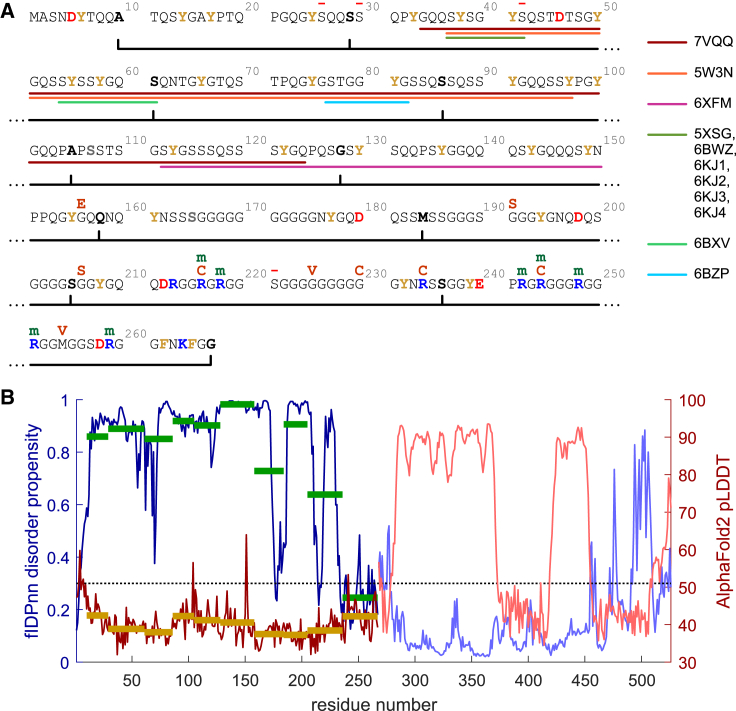
(*A*) Annotated sequence of FUS NTD, comprising the QGSY-rich region (residues 1–165) and the RGG1 domain (residues 166–267). Our construct contains three additional N-terminal residues (GSS) remaining from the linker of a GB1-tag used in protein preparation. Residues that are potentially important in multi-valent interactions driving LLPS are typeset in bold and colored. Residues mutated for spin labeling are typeset bold and black (mutations for ensemble structure determination) or gray (mutations for ensemble validation). The black lines with vertical pointers to spin-label sites visualize sections for which distance distributions were measured. Colored lines denote sections for which structures have been deposited in the PDB, with access codes given in the legend on the right. Crimson single-letter codes above the sequence denote established mutations known to cause amyotrophic lateral sclerosis. Red minus signs above the sequence denote phosphorylation sites and green letters “m” denote arginine methylation sites. The information was compiled from UniProt entry P35637. (*B*) Disorder predictions for full-length FUS by fIDPnn (23) (*blue/light blue and left y axis*) and AlphaFold2 (23) (*red/pink and right y axis*). FUS NTD (1–267) is shown in dark colors and the remainder of the protein in light colors. The horizontal dotted line denotes the disorder threshold of fIDPnn. Thick horizontal lines are mean values of the fIDPnn (*green*) and AlphaFold2 prediction (*orange*) for the chain sections for which we measured distance distributions. To see this figure in color, go online.

The multifunctional RNA-binding protein fused in sarcoma (FUS) is a component of stress granules and is involved in numerous cellular processes, such as DNA repair and several RNA processing steps, including transcription, splicing, transport, and translation (17,18). FUS comprises several IDRs, including an N-terminal domain (NTD) rich in glutamine, glycine, serine, and tyrosine (QGSY-rich region) and three arginine-glycine-glycine-rich domains (RGG domains). In addition, it contains two globular domains, an RNA-recognition motif, and a zinc finger. The QGSY-rich region of FUS (residues 1–165) can phase separate as a separate entity (19). This process is driven by a combination of hydrophobic, hydrogen bond, and *π*-interactions (20). The interactions are not confined to a single protein region and involve all major residue types. This protein region remains disordered upon liquid demixing, and there is no indication for the formation of any secondary structure elements (19,20). The phase-separating properties of the QGSY-rich region are further enhanced in the presence of the RGG1 domain (residues 166–267), stemming probably from cation-*π* interactions between the aromatic residues in the QGSY-rich domain and the arginine residues in the RGG1 domain (21,22). This NTD of the protein is the main driver of liquid-liquid phase separation of FUS (22) and is the focus of this work. The annotated sequence of FUS NTD, comprising the QGSY-rich region and the RGG1 domain, is shown in Fig. 1 *A*. Fig. 1 *B* shows disorder predictions by fIDPnn (23), which performed consistently well in a recent critical assessment of protein intrinsic disorder prediction (24) and by AlphaFold2 (25), whose pLDDT confidence prediction was found to be a competitive predictor of disorder (26). AlphaFold2 predicts the whole NTD to be disordered, whereas fIDPnn reports part of RGG1, in particular near the C terminus of this domain, as weakly ordered.

Based on distance distribution measurements by EPR in agarose-gel-stabilized biphasic samples of FUS (16) and analysis of the data in terms of Gaussian distributions (27), we here derive ensemble models for FUS NTD dispersed in the protein-poor phase and FUS NTD condensed to a protein-rich phase after LLPS. We compare these ensembles to an ensemble for denatured FUS NTD in a buffer containing 3 M urea and to an unrestrained ensemble generated by an approach (28) that takes into account only residue-specific Ramachandran statistics. The paper is structured as follows. First, we demonstrate that all 10 sections of FUS NTD for which we measured distance distributions are slightly more compact in the condensed state than in the dispersed state, whereas the denatured state is the least compact one. We then show that ensemble models of all three states are approximated well by polymer random coils in a good solvent. By considering the deviation of section root-mean-square (RMS) end-to-end distances from expectations for a random coil (29) we find subtle differences between the ensembles, in particular for the section 128–158, which is more extended in the denatured and dispersed states. We demonstrate that, despite the random-coil character, positive and negative charge density as well as the propensities to act as cationic or aromatic sites in cation-*π* interactions remain spatially separated. We consider the correlation between the radius of gyration and asphericity (30) in the ensemble model for the condensed state as well as hydration of the QGSY-rich and RGG1 domains. Finally, we discuss our results in the context of previous work on LLPS of the QGSY-rich region of FUS, of polymer physics, and of thermodynamics.

## Materials and methods

All reagents were purchased from Merck (Sigma-Aldrich) unless otherwise specified.

### Protein expression and purification

*Escherichia coli* strain BL21 (DE3) was used to express histidine- and GB1-tagged FUS NTD (1–267) inserted into a pET24b vector. Cells were grown at 37°C in M9 media prepared with D_2_O for deuterated samples, or in LB-broth medium (BD Difco) for nonisotopically labeled protein. The media were supplemented with kanamycin (PanReac AppliChem) as a selective agent. Once the optical density (OD) at 600 nm reached 0.6, over-expression was induced by addition of 20 *μ*M isopropyl-*β*-D-1-thiogalactopyranoside (PanReac AppliChem) and subsequently incubated at 20°C for approximately 16 h.

Cell pellets were harvested by centrifugation 10 min at 5000 rpm and 4°C and suspended in lysis buffer (50 mM HEPES, 150 mM NaCl, pH 7.5) followed by sonication. Although the soluble supernatant was discarded, the insoluble pellet was resuspended in resuspension buffer (50 mM HEPES, 0.5 M NaCl, 8 M urea, pH 7.5). The sample was loaded onto a nickel column (Qiagen) and washed first with 15–20 mL of wash buffer A (50 mM HEPES, 0.5 M NaCl, 1 M urea, pH 7.5) and then with 15–20 mL of wash buffer B (50 mM HEPES, 150 mM NaCl, 1 M urea, 20 mM imidazole, pH 7.5). The protein was eluted with 10 mL of elution buffer (50 mM HEPES, 150 mM NaCl, 1 M urea, 250 mM imidazole, pH 7.5). Subsequently, imidazole was removed and the His-tag connected by a Tobacco Etch Virus (TEV) cleavage site was cleaved simultaneously during two dialysis steps (using Spectra/Por dialysis tubing with a molecular weight cutoff of 3 kDa), first against dialysis buffer D1 (50 mM HEPES, 150 mM NaCl, 1 M urea, 5 mM *β*-mercaptoethanol (PanReac AppliChem)) and then against dialysis buffer D2 (50 mM HEPES, 150 mM NaCl, 6 M urea, pH 7.5). The sample was loaded onto a second nickel column to remove the cleaved tag and any nonspecifically binding molecules. Protein was collected from the flow-through and subsequently concentrated up to 2 mM, unless otherwise specified. Constructs carrying cysteine mutations were concentrated to approximately 100 *μ*M concentration. The purity of the samples was analyzed on a 13% SDS gel. Protein samples were stored at −80°C until further use.

### PCR mutagenesis

Point mutations were introduced by a three-step PCR protocol (see Table 1) using primers carrying the desired base change. The PCR reaction contained 5 *μ*L of 10× Pfu buffer (Promega); 4 *μ*L of deoxyribonucleotide triphosphate solution (containing 2.5 mM of each deoxyribonucleotide triphosphate, Invitrogen); 0.5 *μ*L of the forward and backward primer at 100 *μ*M, respectively; 1 *μ*L DNA template at approximately 50 ng/*μ*L; 1 *μ*L of dimethyl sulfoxide (NEB); 1 *μ*L of Pfu polymerase (Promega); and addition of Milli-Q water to a total volume of 50 *μ*L. In some instances, Phusion polymerase (NEB) with 10 *μ*L of 5× Phusion buffer (NEB) was used as an alternative to Pfu polymerase and 10× Pfu buffer.

**Table 1:** PCR Protocol Employed for FUS Mutagenesis

Step	Temperature	Time
Initial denaturation	95°C	1 min
23 cycles	95°C	20 s
	55–71°C	30 s
	72°C	8 min
Final extension	72°C	5 min
Hold	4°C	–

The methylated, nonmutated parental DNA template was then digested by addition of 1 *μ*L of Dpn I (NEB) to the PCR product and incubation at 37°C for approximately 1–2 h. Subsequently, the Dpn I-treated DNA was amplified in *E. coli* cells (strain TOP10) and isolated using a MiniPrep kit (Qiagen or Macherey-Nagel). For constructs with several mutations, the protocol was applied repeatedly. The incorporation of the correct mutation was verified by Sanger sequencing (Microsynth).

### Site-directed spin labeling

The protein solution was diluted to a protein concentration of 30 *μ*M and incubated for approximately 1 h with 5 mM DTT (Dithiothreitol, PanReac AppliChem). The reducing agent was washed out with a desalting column packed with G-25 resin (GE Healthcare). Depending on the sample volume either a PD10, PD MidiTrap, or PD MiniTrap column was used for the desalting steps. The sample was then incubated for approximately 2 h with a fivefold to 10-fold excess of the nitroxide spin label ((1-oxyl-2,2,5,5-tetramethylpyrroline-3-methyl)methanethiosulfonate, Toronto Research Chemicals). Subsequently, free spin label was washed out using a desalting column packed with G-25 resin. The protein was then concentrated in Amicon Ultra-4 Centrifugal Filter Units to a concentration of 900 *μ*M. These steps were performed in dialysis buffer D2 to keep the protein soluble. The labeling efficiency was determined by continuous-wave (CW) EPR spectroscopy.

Iodoacetamido and maleimido spin labels were also tested, but protein precipitated upon incubation with these labels and free spin label was still present in the samples after extensive washing.

### Sample preparation in agarose gel

#### General procedure

To form stabilized liquid droplets of FUS, agarose buffer (30 mM HEPES, 200 mM KCl, 0.5% (w/v) agarose (Thermo Fisher), pH 7.3) was boiled to solubilize agarose powder and then cooled at room temperature. Agarose buffer was still liquid at approximately 55°C and was added to an Eppendorf tube containing the protein sample. The mixture was quickly transferred to a preheated sample tube (∼55°C). Due to the small volume used, the temperature drops quickly, leading to liquid-droplet formation and agarose gelation. The residual urea concentration was 0.6 M. The same procedure was used for the denatured-state sample, with the exception that, in addition, the agarose gel contains 3 M urea to keep the protein soluble.

#### Agarose-gel sample preparation for DEER experiments

Spin-labeled protein was diluted 1:10 in dialysis buffer D1 yielding a protein concentration of approximately 90 *μ*M in 50 mM HEPES, 150 mM NaCl, 1.5 M urea, pH = 7.5 buffer. To minimize intermolecular distance contributions, we added wild-type protein for spin dilution purposes. The stock concentration of the wild-type protein was approximately 1.25 M for denatured samples, and 2.5 mM for biphasic samples (both in dialysis buffer D2). Spin-labeled protein and the wild-type protein were premixed in an Eppendorf tube to yield a spin dilution factor of 1:10 for denatured samples and 3:80 for biphasic samples (total volume: 6.3 *μ*L). Boiled agarose buffer (containing 3 M urea for denatured samples) at a temperature of approximately 55°C was added to the protein mixture to yield a final protein concentration of 50 *μ*M for denatured samples and 200 *μ*M for biphasic samples at a total volume of 40 *μ*L. Then 35 *μ*L were then transferred to a preheated sample tube (∼55°C, 3 mm OD fused quartz, 0.3±0.1 mm wall thickness, Aachener Quarzglas Technologie Heinrich) and the mixture was allowed to cool down before shock-freezing in pre-cooled isopentane.

Dispersed monophasic samples were prepared with a protein concentration of 5 *μ*M to ensure that the protein does not undergo phase separation. These samples were prepared under the same buffer conditions as biphasic samples. Spin-labeled protein was premixed with dialysis buffer D1 and D2 to a total volume of 6.3 *μ*L (the same volume used for the biphasic sample preparation before the addition of agarose buffer to ensure the same buffer composition). The dialysis buffers D1 and D2 were added at appropriate ratios to achieve a final urea concentration of 0.6 M after addition of all components. Warm agarose buffer (30 mM HEPES, 200 mM KCl, 0.5% (w/v) agarose, pH 7.3) was then added to this mixture to yield 40 *μ*L, and 35 *μ*L of the sample was transferred to a preheated tube. Once the sample temperature dropped to room temperature, the sample was shock-frozen in pre-cooled isopentane.

All samples were prepared with water of natural isotope abundance, except for the denatured-state sample for the four-pulse DEER measurement, which was prepared with D_2_O to prolong the phase memory time.

Samples were stored in a liquid-nitrogen container.

### Light microscopy

Differential contrast imaging was performed at the ScopeM facility with a Leica DMI6000B microscope and using a 63×1.4 NA oil objective. The 20-*μ*L samples with 50 *μ*M total protein concentration prepared in agarose buffer were loaded onto a glass coverslip (Bellco) and covered with an additional smaller coverslip (Menzel). To minimize evaporation, samples were sealed using nail polish (Electron Microscopy Sciences).

### Turbidity measurements

For turbidity measurements, 20-*μ*L liquid-droplet samples (50 *μ*M total protein concentration) in agarose buffer were prepared as described above and pipetted while liquid onto 384-well plates (CORNING 4581). Turbidity at 600 nm was measured using a Synergy 2 (Biotek) microplate reader at room temperature. Agarose buffer was used as blank. All samples were examined in triplicate.

### Pulsed EPR measurements

Pulsed EPR measurements were performed on a homebuilt Q-band spectrometer (31) or a Bruker Elexsys E680 spectrometer equipped with an incoherent arbitrary waveform generator (32). A temperature of 50 K was achieved by liquid-helium cooling and was controlled by a He flow cryostat (ER 4118CF, Oxford Instruments) and a temperature control unit (ITC 503, Oxford Instruments). A sample tube loaded with 35 *μ*L of sample was shock-frozen in pre-cooled isopentane and subsequently inserted into a homebuilt Q-band resonator for 3-mm OD sample tubes (31).

An echo-detected field-swept EPR spectrum was acquired using a Hahn-echo sequence. The pulse sequence for the four-pulse DEER experiment was $\pi /2_{\text{obs}}-\tau_{1}-\pi_{\text{obs}}-t_{1}-\pi_{\text{pump}}-\left(\tau_{1}+\tau_{2}-t_{1}\right)-\pi_{\text{obs}}-\tau_{2}$. The pump pulse was applied on the spectral maximum and the observer pulses were applied at a frequency offset of 100 MHz. The 4-pulse DEER measurements on the Bruker Elexsys E680 spectrometer were acquired with a pulse delay $\tau_{1}$ of 200 ns and a dead-time delay of 80 ns. Measurements on the homebuilt Q-band spectrometer were acquired with a pulse delay $\tau_{1}$ of 400 ns and a dead-time delay of 280 ns. All traces were acquired using 16-ns pulses and eight-step nuclear modulation averaging with an averaging time step of 16 ns.

For the five-pulse version, the experiment was recorded with the sequence $\pi /2_{\text{obs}}-\left(\tau /2-t_{0}\right)-\pi_{\text{pump}}-t_{0}-\pi_{\text{obs}}-t^{\prime }-\pi_{\text{pump}}-\left(\tau -t^{\prime }+\delta \right)-\pi_{\text{obs}}-\left(\tau /2+\delta \right)$ with *δ* = 120 ns to separate the stimulated echo from the refocused echo. The 5-pulse DEER data were recorded with HS{1,6} pump pulses of 150 MHz in width and conventional 32-ns observer pulses at a frequency offset of 70 MHz. Two traces with shifted artifact were recorded alternately. For the first trace, $t_{0}$ was set to 300 ns. For the second trace, $t_{0}$ and the initial value of the delay t’ before the moving pump pulse were increased by a multiple of the time increment, i.e., 192 ns. For details on the nuclear modulation averaging, see the supplementary information in (33).

### DEER data analysis

All DEER data were analyzed using a Matlab-based version of DeerLab (release 0.9.2, available at github.com/JeschkeLab/DeerLab-Matlab) (34) by fitting a single Gaussian distribution as validated earlier (27) and below.

We used a multi-pathway kernel K(t,r), which allows fitting of a distance distribution to four-pulse and five-pulse primary DEER data V(t), including any additional contributions that might arise from other pathways. This kernel has the form

(1) $$K\left(t,r\right)=\left[\lambda_{0}+\lambda_{1}K_{0}\left(t,r\right)+\lambda_{2}K_{0}\left(t-T_{0,2},r\right)\right]B\left(t\right)\text{,}$$

where $K_{0}$ is the elementary dipolar kernel, $\lambda_{0}$ accounts for the contribution of unmodulated dipolar pathways, $\lambda_{1}$ and $\lambda_{2}$ describe the amplitudes of the modulated dipolar pathways, and $T_{0,2}$ is the refocusing time of the additional modulated dipolar pathway. The background function B(t) is a product of stretched exponential functions as specified below. Although $K_{0}$ is fixed, $\lambda_{0}$, $\lambda_{1}$, $\lambda_{2}$, $T_{0,2}$, and the parameters that specify B(t) are fit parameters. For all fitted signals, distributions, and parameters, bootstrapped confidence intervals were estimated from 1000 bootstrap samples drawn from a Gaussian distribution based on the noise in the measurements. The primary DEER data and output files of data analysis are found in database Zenodo: zenodo.8214049.

#### Analysis of the denatured state and the dispersed monophasic state

The DEER data were modeled according to the kernel above with an exponential background function (d=3). Distance distributions were fitted by a single Gaussian distribution.

#### Analysis of the biphasic state

Due to the presence of the two different species in the biphasic sample, we modeled the measured primary signal as a linear combination of two dipolar signals $V_{\text{disp}}\left(t\right)$ corresponding to spins in the dispersed fraction and $V_{\text{cond}}\left(t\right)$ to those in the condensed phase weighted by the fractions $f_{\text{disp}}$ and $1-f_{\text{disp}}$. Using the appropriate kernel functions $K_{\text{disp}}\left(t,r\right)$ and $K_{\text{cond}}\left(t,r\right)$, which differ by their background function, this model can be expressed as

(2) $$V\left(t\right)=f_{\text{disp}}\int K_{\text{disp}}\left(t,r\right)P_{\text{disp}}\left(r\right)dr+\left(1-f_{\text{disp}}\right)\int K_{\text{cond}}\left(t,r\right)P_{\text{cond}}\left(r\right)dr\text{.}$$

The two components of the signal arise from different distance distributions originating from the dispersed $P_{\text{disp}}\left(r\right)$ and condensed $P_{\text{cond}}\left(r\right)$ fractions, which were modeled as two Gaussian distributions of the form

(3) $$P\left(r\right)=\frac{1}{\sigma \sqrt{2\pi }}e^{-\frac{{\left(r-\left\langle r\right\rangle \right)}^{2}}{2\sigma^{2}}}$$

where ⟨r⟩ is the mean distance and *σ* the standard deviation.

Due to the difference in spin concentration inside and outside of the liquid droplets, they also exhibit significantly different background functions: a slowly decaying one

(4) $$B_{\text{disp}}\left(t\right)=e^{-k_{\text{disp}}\left(\lambda_{1}\left|t\right|+\lambda_{2}\left|t-T_{0,2}\right|\right)}$$

for the dispersed fraction with a very constrained decay rate $k_{\text{disp}}$ due to the low local spin concentration, and a rapidly decaying function

(5) $$B_{\text{cond}}\left(t\right)=e^{-k_{\text{cond}}\left(\lambda_{1}{\left|t\right|}^{d_{\text{cond}}/3}+\lambda_{2}{\left|t-T_{0,2}\right|}^{d_{\text{cond}}/3}\right)}\text{,}$$

for the condensed fraction. For the dispersed phase we employed an exponential background function ($d_{\text{disp}}$ = 3) as the basis function for the multi-pathway background, whereas for the condensed phase we employed a stretched exponential function as the basis function.

We performed a global analysis (35) of two five-pulse DEER signals, whose secondary pathway is shifted in time with respect to each other. All model parameters ($f_{\text{disp}}$, $\lambda_{0}$, $\lambda_{1}$, $\lambda_{2}$, $d_{\text{cond}}$, $k_{\text{disp}}$, $k_{\text{cond}}$, ${\left\langle r\right\rangle }_{\text{disp}}$, ${\left\langle r\right\rangle }_{\text{cond}}$, $\Gamma_{\text{disp}}$, and $\Gamma_{\text{cond}}$) were fitted globally to both datasets, except for the refocusing time $T_{0,2}$, which was fitted locally to each dataset. We constrained $f_{\text{disp}}$ to ±10% of the fraction of proteins in the dispersed phase as determined by Diffusion Order SpectroscopY (DOSY) NMR experiments and restricted the parameter range of $P_{\text{disp}}\left(r\right)$ to the upper and lower 95% confidence interval of the mean and full width at half maximum of the dispersed monophasic distance distribution. Global optimization was employed by means of a multi-start algorithm to ensure that the solution is a global solution.

### Ensemble generation and analysis

Ensembles for unrestrained, denatured, dispersed, and biphasic FUS NTD were generated with the software package MMMx (commit f1e3459 from 6 December 2021, downloadable at GitHub: https://github.com/gjeschke/MMMx) (29) with dependencies on SCWRL4 (36). The raw ensemble generation, ensemble fit calculations, and ensemble analysis were performed on the ETH Euler cluster. Protein ensembles were analyzed and visualized with MMMx (commit ddff3f8 from 5 May 2023).

Restrained and unrestrained ensembles were generated using the Flex module and the FUS NTD sequence, including the residual N-terminal sequence GGS left over from TEV cleavage. Flex is a Monte-Carlo conformer generator that samples from residue-specific Ramachandran angle statistics and can consider distance distribution restraints already during backbone building by rejecting conformers as soon as their probability to conform to the restraints drops below a threshold value; 5000 conformers were generated for each ensemble in several runs. In the restrained version, distance distributions were specified by mean distances and standard deviations of the Gaussian fits. The restraint files used for the ensemble generation and all ensemble models can be found in database Zenodo: zenodo.8214049. The restrained raw ensembles were contracted in an ensemble-fitting step using the Gaussian restraints. Ensemble fitting maximizes overlap of the experimental distance distributions with distance distributions simulated the ensemble. In simulation of the distance distributions, spin-label conformation distribution is accounted for by a rotamer library approach. Conformers with populations (weights) smaller than 1% of the largest population are discarded in ensemble fitting. Ensemble fitting of the full raw ensembles converged for the denatured and dispersed ensembles with final sizes of 181 and 551 conformers, respectively. Ensemble fitting of the condensed ensemble was performed on five batches of 1000 conformers each. The contracted ensembles were then combined and an additional ensemble-fitting step was performed with the reduced ensemble to yield a final representative ensemble of 2102 conformers.

The unrestrained ensemble and the representative restrained ensembles were analyzed using the EnsembleAnalysis module of MMMx. The additional GGS residues at the N terminus were left out in plots. Conformers within each ensemble were superimposed by aligning the principal axes systems of their inertia tensors. In the new frame, the *x* axis corresponds to the minimum moment of inertia and the *z* axis to the maximum moment of inertia. The N terminus has a smaller *x* and *z* coordinate than the C terminus. With the three principal values $I_{x}$, $I_{y}$, and $I_{z}$ of the inertia tensor, asphericity *A* of a conformer is computed as (37,38)

(6) $$A=1-3\frac{I_{2}}{I_{1}^{2}}\text{,}$$

where $I_{1}=I_{x}+I_{y}+I_{z}$ and $I_{2}=I_{x}I_{y}+I_{x}I_{z}+I_{y}I_{z}$. For computing mean asphericity ⟨A⟩ of the ensemble, $I_{2}$ is replaced by $\left\langle I_{2}\right\rangle$ and $I_{1}^{2}$ is replaced by $\left\langle I_{1}^{2}\right\rangle$.

Ensemble visualization is based on population-weighted electron density maps of the inertia-frame superimposed conformers. Electron density maps of individual conformers were computed by implementation of an earlier approach (39) in MMMx. The approaches for coloring of isosurfaces of the density maps for electrostatic interaction and cation-*π* interaction are described in the supporting material. Residue weightings for cation-*π* interaction are based on (40).

### Analysis of hydration

Hydration of FUS NTD conformers was assessed using the Accutar Open Access platform as described in (41). We first sorted the conformers in the ensemble describing the condensed state by similarity according to a distance RMS deviation criterion. Then we submitted PDB files for the first 25 and last 25 conformers to the Accutar Bio Server. Coordinates of the oxygen atoms of the generated water molecules were extracted from the output by the auxiliary MMMx function rd_accutar.m. We identified the coordinated residue in the input PDB file as the residue harboring the protein atom with the shortest distance to the water oxygen atom. Data were analyzed in terms of the average number of water molecules, the standard deviation of this number, and the mean number of water molecules coordinated to each residue type using a Matlab script. We found these numbers to be converged after analyzing 50 conformers at the level required for our discussion.

## Results

### FUS NTD compacts over its whole length upon LLPS

Distance distributions in the nanometer range can provide restraints for ensemble modeling of IDRs of proteins (28). In recent work, we modeled the conformational ensemble of the glycine-rich domain (133 residues) of the RNA-binding protein heterogeneous nuclear ribonucleoprotein (hnRNP) A1 based on 19 distance distribution restraints and demonstrated by jackknife resampling that the model was sufficiently restrained (27). In later work, we found this model to be in good agreement with a small-angle x-ray scattering (SAXS) curve and in reasonable agreement with paramagnetic relaxation enhancement NMR data (42). The case of FUS in biomolecular condensates is more challenging for three reasons. First, the presence of a zinc finger in full-length FUS interferes with spin labeling. Therefore, we focus on the NTD comprising residues 1–267. However, this deprives us of a folded domain and thus of reference spin-labeling sites that could be used for the relative localization of the IDRs (“beacon” sites). Second, the distance range of DEER measurements depends critically on phase memory time of electron spins, which is therefore prolonged often by using deuterated solvent and sometimes by using deuterated protein (43). With deuterated FUS NTD, we found substantially different phase separation behavior than with natural isotope abundance protein (Fig. S1 in supporting material). Neither is it advisable at our current understanding to deuterate the solvent, as this has been found to affect phase transition temperatures of polymer hydrogels (44) as well as small-angle neutron scattering of protein gels (45). It has been pointed out that substitution of water protons by deuterons modifies the balance between intramolecular and hydration interactions (46). Since Terahertz spectroscopy has revealed that hydration interactions change upon LLPS (47,48), we refrain from solvent deuteration in the current study. We have recently established methodology (16) based on the five-pulse DEER experiment (49), which provides access to distances up to about 60 Å in FUS liquid droplets for natural isotope composition. However, this is still limiting for a construct with 267 residues. Third, paramagnetic relaxation enhancement measurements in the condensate by NMR spectroscopy do not provide spatial information (20) and SAXS does not provide information on the conformational distribution of individual protein molecules in a condensate. In principle, protein deuteration could provide contrast in small-angle neutron scattering, but such a strategy is not advisable, as discussed above. Hence, we are deprived of the advantages (50) of integrating distance distribution restraints with data from other techniques for ensemble structure determination.

In this situation, we opted for mapping the conformational ensemble by a set of distance distributions with mean distances near the middle of the accessible distance range (30–40 Å). This entails sequence separations between labels of about 20–30 residues (28). Restrictions on the choice of labeling sites further complicate the situation. The NTD of FUS contains no native cysteines, requiring the introduction of cysteines in engineered positions for site-directed spin labeling. The accessibility of a residue and the potential disruption of the protein’s structure or function need to be considered when introducing mutations with the purpose of site-directed spin labeling. The accessibility of a site in structured protein domains for labeling is typically probed by a rotamer analysis using the protein’s structure (51). In our case, residues in IDRs are expected to be accessible. Our main concern here is potential disruption of the phase separation behavior by the labels. Therefore, we excluded sites if the physicochemical properties of the amino acid differed strongly from the ones of cysteine, the candidate involved a mutation site previously identified in patients (52), the mutation involved amino acids or motifs relevant for phase separation (21), or the residues are involved in RNA binding (53). Avoiding sites within the NTD that can form fibrillar assemblies (54,55,56,57,58) (Fig. 1) was not always possible. There is no indication that structured states are formed upon phase separation of FUS NTD (16,20), and, hence, mutations on the fibril cores should not influence LLPS.

With these considerations in mind, we selected the mutation sites A10, S29, S61, S86, A105, G128, Q158, M184, S205, S236, and G267, which cover the entire NTD. These mutation sites divide the NTD into 10 sections that are flanked by two engineered cysteines, allowing us to characterize the domain by intramolecular distance measurements by EPR. Six sections cover the QGSY-rich region, three the RGG1 domain, and one section is localized in the boundary between both domains. Three additional double cysteine mutants were generated for ensemble validation: S89C S107C, S165C M184C, and M184C S236C. We spin labeled all constructs with a methanethiosulfonate spin label ((1-oxyl-2,2,5,5-tetramethylpyrroline-3-methyl)methanethiosulfonate), which is the size of a large native amino acid side chain with a hydrophobicity in between that of methionine and tryptophan, suggesting a comparatively small perturbation on the system (59). Importantly, spin-labeled cysteine double mutants retain the ability to phase separate (Fig. S2 *A*) with similar turbidity values to the wild-type protein (Fig. S2 *B*).

**Figure 2:**
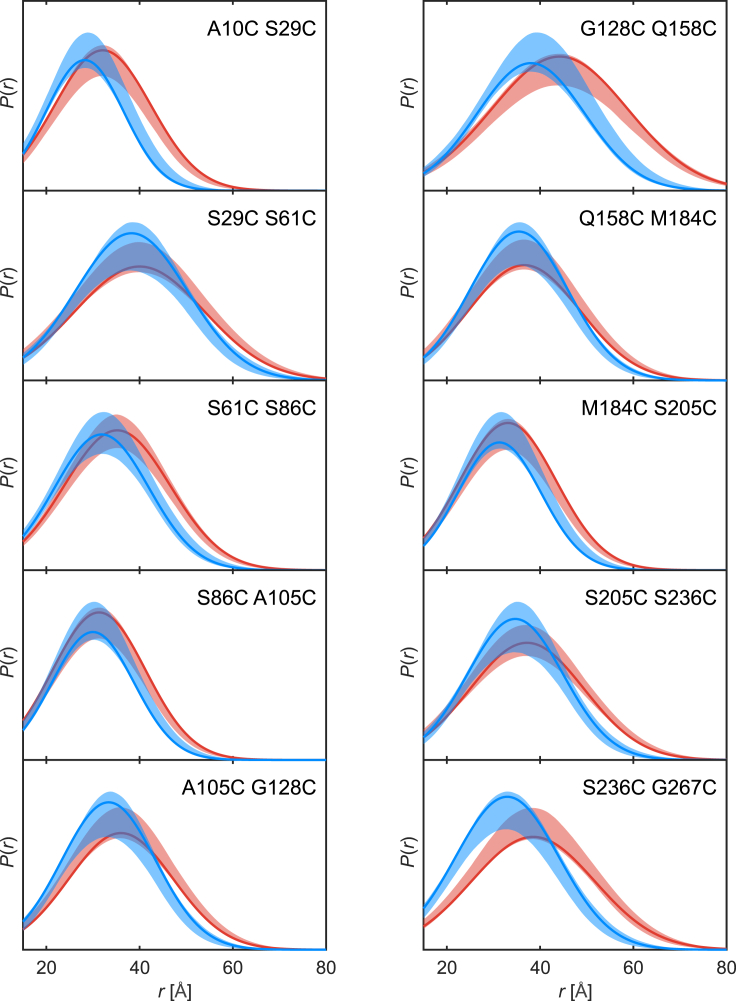
Distance distribution restraints of the NTD in the biphasic state. Spin-label positions are indicated in the upper right corner of each panel. The dispersed fraction is shown in red, the condensed fraction in blue, and the 95% confidence intervals obtained via 1000 bootstrap samples as shaded areas. The primary DEER data are visualized in Fig. S7 and the fit residuals in Fig. S8. To see this figure in color, go online.

In previous work, we established by ensemble simulations that distance distributions for spin-labeled peptide random coils are well approximated by Gaussians (27). In experiments for two of the double mutants of FUS, we found that Gaussian distributions fitted experimental DEER data well for the dispersed monophasic state, the biphasic state, and bulk condensed FUS NTD (16). We checked that distance distributions back-calculated from an unrestrained ensemble of FUS NTD are also very well approximated by Gaussians (Fig. S3). By analyzing DEER data for biphasic FUS NTD samples in terms of Gaussian distributions for the dispersed fraction (red) and condensed fraction (blue), we find that all 10 sections are more compact in the biomolecular condensate (Fig. 2). This confirms and extends our earlier finding (16) of compaction of FUS NTD upon LLPS. Note that starting values for the fit parameters for the dispersed state (Table S1) were derived from measurements on monophasic dispersed samples (Fig. S4). The primary data for the monophasic dispersed (Fig. S4) and biphasic (Fig. S5) samples are fitted very well by the Gaussian distributions, as is apparent also from the fit residuals for the biphasic sample (Fig. S6). For comparison, we measured DEER data for all double mutants for FUS NTD in its denatured state (3 M urea), which are also well fitted by Gaussian distributions (Fig. S7 for primary data and Fig. S8 for corresponding distance distributions). Parameters of the Gaussian fits for the denatured state and for the dispersed and condensed fraction in the biphasic state are shown in Table S2. Not unexpectedly, the mean distance is longer for all sections in the denatured state than in the dispersed state. Hence, FUS NTD extends upon adding 3 M urea, whereas it compacts upon undergoing LLPS. We cannot safely exclude different urea concentrations in the dispersed and condensed phase as a reason for the compaction, but we will see below that compaction is in line with expectations from polymer physics without invoking an effect due to urea.

### Dispersed and condensed states behave as random coils under good-solvent conditions

Based on the restraints in Table S2, we modeled three ensembles of FUS NTD that we call the denatured, dispersed, and condensed ensemble in the following. They were deposited in the Protein Ensemble Database with accession numbers PED00495, PED00494, and PED00493, respectively. Given the very similar restraints for the dispersed state obtained in a dilute monophasic sample and the biphasic sample, we computed only one dispersed-state ensemble based on the restraints from the biphasic sample. In a first step, we generated raw ensembles using residue-specific Ramachandran angle distributions for sampling and the Gaussian restraints for rejecting conformers that were in disagreement with the experimental data (28). In a second step, we reweighted the ensembles by fitting populations (29,50). This step provides smaller ensembles, as we reject conformers with populations that are by more than a factor of 100 smaller than the largest conformer population. Distance distributions back-calculated from the such obtained ensembles are in virtually perfect agreement with the Gaussian distributions (Figs. S8–S10), as quantified by their overlap

(7) $$o=\sum_{i}\min\left\{P_{\text{sim}}\left(r_{i}\right),P_{\exp}\left(r_{i}\right)\right\}\text{,}$$

where $P_{\text{sim}}\left(r\right)$ and $P_{\exp}\left(r\right)$ are the simulated and experimental distance distributions, given as normalized vectors. The overlap can range from 0 for disjoint to 1 for identical distributions. We find values between 0.979 and 0.996, much closer to unity than for the weakly structured glycine-rich IDR of hnRNP A1 (27,42). For the denatured ensemble with only 181 conformers, the overlap deficiency 1−o of up to 0.022 is most likely dominated by sampling statistics. For the dispersed and condensed ensemble, overlap deficiency is within the combined uncertainties from the experiment and from assuming a Gaussian distribution.

**Figure 3:**
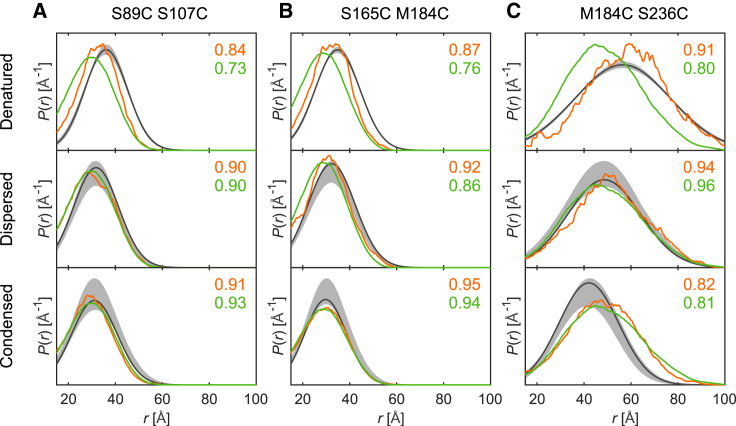
Ensemble validation by comparison of experimental and predicted distance distributions. Experimental distance distributions (*gray*) of (*A*) S89C S107C, (*B*) S165C M184C, and (*C*) M184C S236C in the denatured (*top row*), dispersed (*middle row*), and condensed (*bottom row*) states compared to predicted distance distributions of the corresponding ensemble (*orange*) and the unrestrained ensemble (*green*). Overlap values between experimental and predicted distributions are color coded and displayed in the upper right corner of each plot. Shaded gray areas denote 95% confidence intervals obtained via 1000 bootstrap samples. To see this figure in color, go online.

For ensemble validation, we measured data for the three additional double mutants (S89C S107C, S165C M184C, and M184C S236C) in the denatured and biphasic states (Fig. 3). Overlap deficiency of the corresponding ensembles is larger than for the distributions used in ensemble fitting and ranges up to 0.18 (M184C S236C condensed state). The deviations are highly significant only for S89C S107C and S165C M184C in the denatured state and for M184C S236C in the condensed state. For the broader distribution M184C S236C, overlap deficiency is dominated by sampling statistics for the denatured ensemble (181 conformers) and dispersed ensemble (561 conformers). Back-calculated distributions S89C S107C and S165C M184C are rather close to the uncertainty bands for the dispersed and condensed ensembles. The ensemble model of the denatured state underestimates the extension with respect to the unrestrained ensemble for S89C S107C and S165C M184C. This indicates that adding more experimental data could still improve the model. The situation is less clear-cut for M184C S236C in the condensed fraction of the biphasic sample. We cannot safely exclude that the experimental uncertainty estimate is too optimistic, as the primary DEER data is rather short for detecting distances up to 80 Å (43). For this reason, we did not attempt to measure further double mutants with a sequence separation of more than 50 between spin labels.

**Figure 4:**
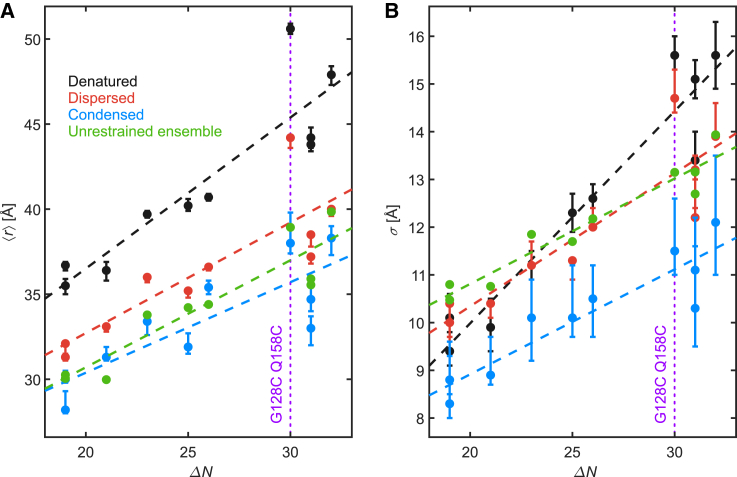
Overview of the Gaussian fit parameters of all ensembles. (*A*) Mean distances ⟨r⟩ and (*B*) standard deviations *σ* of experimental Gaussian distance distributions for site pairs in FUS NTD in the denatured (*black*), biphasic dispersed (*red*), and biphasic condensed state (*blue*) compared to the back-calculated values of the unrestrained ensemble (*green*) as a function of the sequence separation ΔN between the labeled residues. Error bars correspond to the 95% confidence intervals. Linear fits (*dashed lines*) are a guide to the eye. The conspicuous section G128C Q158C is marked by the purple vertical line. Note that more than one section can have the same sequence length ΔN. To see this figure in color, go online.

We compared the restraint sets for the three ensemble models among themselves and to the unrestrained ensemble by plotting the mean values ⟨r⟩ and standard deviations *σ* of the 10 measured sections versus sequence separation ΔN (Fig. 4). Within this range of sequence separations, the dependence of both parameters on ΔN is nearly linear. We thus added linear fits as a guide to the eye. The only section with a highly significant deviation from this linear dependence is G128C Q158C in the denatured and dispersed cases. This section is marked in Fig. 4 by a purple vertical line. A comparison reveals that only the denatured state deviates substantially from the unrestrained ensemble. In the dispersed state, FUS NTD is slightly more extended than in the unrestrained ensemble, with widths *σ* of the distance distributions being very similar to the unrestrained case except for section G128C Q158C. The condensed state exhibits mean distances whose difference to the ones in the unrestrained ensemble is hardly significant. The widths *σ*, although they have rather large error bars, are systematically lower than for the unrestrained ensemble. Altogether, the comparison indicates that all ensembles are rather close to random coils, except for section G128C Q158C in the denatured and dispersed state. Unrestrained protein ensembles are known to fit well to a random-coil model (60). Note that the section G128 Q158 harbors the only known missense mutation within the QGSY-rich domain that causes amyotrophic lateral sclerosis (G156E) (61). This mutation was also shown to increase the propensity of aggregation of FUS in vitro and in cell culture (62). In addition, this extended section overlaps with the fibril structure found for an FUS construct composed of the C-terminal half of the low-complexity domain (residues 111–214) (57).

### Parts of FUS NTD slightly contract or expand compared to a random coil

To obtain further insight into this finding, we rely on the fact that the RMS end-to-end distance $R=\sqrt{\left\langle R_{\text{end}-\text{end}}^{2}\right\rangle }$ of each section of a random coil exhibits the same scaling behavior with section sequence length ΔN. There exist N+1−ΔN sections with sequence separation ΔN, whereas in FUS NTD ΔN is running from 1 to N=267. Hence, we have *R* values for 35,778 distinct chain sections. For a random coil, we expect that *R* for all these sections conforms to the same scaling law with respect to ΔN,

(8) $$R=b\cdot \Delta N^{\nu }$$

where ν=0.5 corresponds to the Gaussian coil encountered in a *θ* solvent, ν=1/3 to the poor-solvent limit, and ν=3/5 to the good-solvent limit in Flory theory of real polymer chains. For the good-solvent limit, more elaborate field theory predicts a value of ν=0.588±0.001 (63). As there exist N+1−ΔN sections with sequence separation ΔN, an ensemble model provides distributions R(ΔN). We have demonstrated before that such distributions can reveal deviations from random-coil behavior (27). The average $\bar{R}\left(\Delta N\right)$ over all sections with the same ΔN is also expected to scale with ΔN according to Eq. 8. Deviation from random-coil behavior can be recognized in this dependence (27).

**Figure 5:**
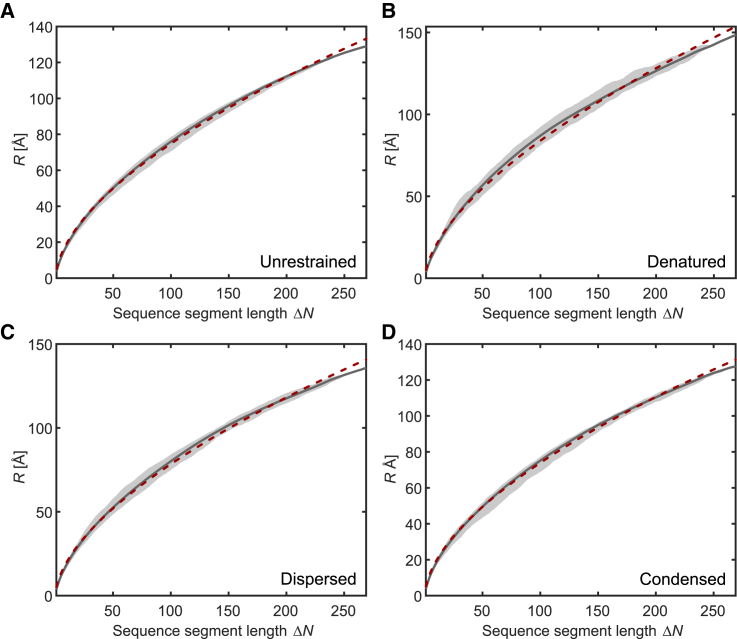
Distribution of RMS C*α*-to-C*α* distances *R* (*gray shaded areas*) for all sections with sequence separation ΔN, their averages $\bar{R}\left(\Delta N\right)$ (*solid gray lines*), and random-coil fits according to Eq. 8 (*crimson lines*) for (*A*) the unrestrained ensemble, (*B*) the denatured ensemble, (*C*) the dispersed ensemble, and (*D*) the condensed ensemble. To see this figure in color, go online.

For all FUS NTD ensembles, we find narrow distributions of *R* at given sequence separation ΔN and rather good agreement of the scaling of $\bar{R}$ with ΔN with random-coil behavior (Fig. 5). For small ΔN, the chains are slightly more extended than expected and for large ΔN they are slightly less extended. We tentatively assign this behavior to the RGG1 domain being slightly more compact (see below) and the QGSY-rich domain contributing more shorter sections due to being longer. Parameters *b* and *ν* of the random-coil models for all ensembles are reported in Table 2 together with ensemble size, radius of gyration $R_{g}$, and the geometric mean $\bar{o}={\left(\prod_{m=1}^{M}o_{m}\right)}^{1/M}$ of the overlaps of all experimental distance distributions for a given ensemble with the distance distributions back-calculated for the same label pairs from the unrestrained ensemble. The empirical repeat unit length b=5.1 Å is the same for all ensembles. It is longer than $R_{0}=1.98\pm 0.37$ Å found by a simpler model of rigid sections with flexible linkers (60). Our value is similar to b=5.5 Å found in the context of interpreting Förster resonance energy transfer (FRET) experiments on intrinsically disordered proteins (64). The scaling exponent of 0.583 for the unrestrained ensemble is very close to the theoretical value of 0.588 for a random coil in a good solvent. The same applies to the dispersed and condensed ensemble, whereas the denatured ensemble features, on average, more extended chains than expected for a random coil. Values of *ν* slightly larger than 0.6 have also been found for denatured proteins in a Förster resonance energy transfer study, albeit at larger concentrations of urea (64). Our finding of good-solvent conditions for FUS NTD in the dispersed state is in contrast to earlier observations for proteins with Q/N/S-rich sequences that undergo LLPS (9), such as polyglutamine (65) and the yeast prion protein Sup35 (66) as well as for polar oligomers (GG)_1_5 and (GS)_8_ (67), which collapse in aqueous solution in their dispersed state. Altogether, the random-coil model appears to be a good approximation for all four ensembles of FUS NTD. However, the small deviations from this model may be significant.

**Table 2:** FUS NTD Ensemble Characteristics

	Size	*b* (Å)	*ν*	$R_{g}$ (Å)	${\bar{o}}_{\text{ref}}$
Unrestrained	5000	5.1	0.583	51.3	–
Denatured	181	5.1	0.610	58.2	0.77
Dispersed	551	5.1	0.592	54.0	0.93
Condensed	2102	5.1	0.581	50.7	0.91

Values for the ensemble size, chain dimension, radius of gyration, and mean overlap with the back-calculated distance distributions of the unrestrained ensemble ${\bar{o}}_{\text{ref}}$.

### The C-terminal section of the QGSY-rich domain appears to be extended

**Figure 6:**
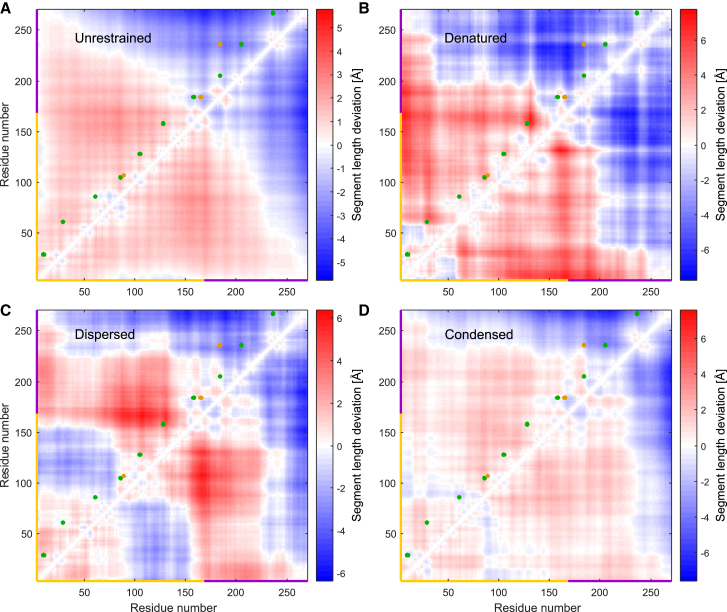
Section-length deviation ΔR from the average RMS C*α*-C*α* distance for a given sequence separation ΔN. The section-length deviations are shown for (*A*) the unrestrained, (*B*) the denatured, (*C*) the dispersed, and (*D*) the condensed ensemble. Blue regions correspond to more compact and red regions to more extended sections, with the corresponding color bars shown on the right. Green points above the diagonal denote pairs of labeling sites used in the generation of the experimental ensembles, whereas orange points denote site pairs used for ensemble validation. The QGSY-rich domain is denoted yellow on the axis, whereas the RGG1 domain is indicated in purple. To see this figure in color, go online.

To obtain more insight into the small deviations from random-coil behavior, we subtract from the section RMS end-to-end distance *R* the average $\bar{R}$ over all sections with the same sequence length ΔN. We compute the differences $\Delta R_{i,j}=R_{i,j}-\bar{R}\left(\Delta N\right)$ for all chain sections from a residue i(i=1…267) to a residue j(j=1…267). Fig. 6 presents all these differences in a two-dimensional plot. Each point corresponds to one pair of residues. Spin-label site pairs used for restraining the ensemble are denoted by green points above the diagonal, whereas validation pairs are denoted by orange points. Red shades denote sections that are more extended than the average, whereas blue shades denote sections that are more compact. In Fig. 5, the ΔR correspond to the vertical difference of a point in the gray area from the solid gray line.

For the unrestrained ensemble (Fig. 6 *A*), we find that the QGSY-rich domain (residues 1–165) is more extended and the RGG1 domain (residues 166–267) more compact than average. Generation of the unrestrained ensemble (28) does not consider any intramolecular interactions beyond clash avoidance and those interactions that contribute to residue-specific Ramachandran plots. Hence, this effect can only be explained by bulk of sidechains and Ramachandran preferences. Indeed, sidechains are, on average, less bulky in the RGG1 domain than in the QGSY-rich domain (Fig. S11 *A*) and the population of extended conformations in residue-specific Ramachandran plots is larger in the QGSY-rich domain (Fig. S11 *B*). Both features are caused mainly by the larger fraction of Gly residues in RGG1.

The experimentally informed ensembles exhibit additional patterns, most strongly for the dispersed state (Fig. 6 *C*), followed by the denatured state (Fig. 6 *B*) and the condensed state (Fig. 6 *D*). As this feature may depend on measurements of a single double mutant, we performed a technical repeat (see Fig. S12; Table S3 in the supporting material) and repeated ensemble fitting for the dispersed state by using the unrestrained raw ensemble and 1) the original distance distribution restraint, 2) the restraint obtained with the technical repeat, and 3) not restrained on section 128–158. The results are compared in Fig. S13 to the result shown in Fig. 6 *C*. We find that the pattern of extension and compaction is the same in all ensembles, although the extent of extension of section 128–158 is substantially reduced when the corresponding restraint is skipped.

The dependence of the slight extension of section 128–158 on data obtained with just one spin-labeled double mutant raises the concern of a spin-labeling artifact. Molecular dynamics simulations on amyloid-*β*42 suggested a bias to the conformation ensemble due to insertion of a spin label at the N terminus (68). Kinetics of fibrilization of tau peptides was found to be affected by spin labels at some sites (69). Given that the mean distance between sites 128 and 158 is much larger than the combined size of the two labels and that the two other sections containing one of the two labeling sites, 105–128 and 158–184, have inconspicuous chain extensions, we consider it rather unlikely that the effect is due to the spin labeling. Further work is needed to verify special behavior of section 128–158 and understand its cause.

### Properties of the QGSY-rich and RGG1 domains differ despite the random-coil behavior

**Figure 7:**
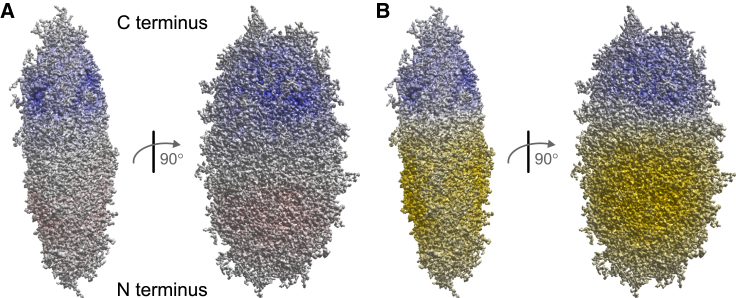
Visualization of (*A*) electrostatic and (*B*) cation-*π* interaction potentials of FUS NTD in the condensed state. The spatial extension of the ensemble is visualized by transforming all conformers to the principal axes frame of their inertia tensor (*x axis upright*) and computing a population-weighted pseudo-electron density of the ensemble. Electrostatic potential is visualized from blue (positive) to red (negative) by adding contributions of individual charged residues screened only by the ionic strength of the buffer (150 mM NaCl). Cation-*π* potential is visualized by adding contributions of individual cations (*blue*) and aromatic residues (*gold*) and assuming decay by the inverse square of the distance. For clarity, full color saturation is assigned to the maximum surface potential encountered. To see this figure in color, go online.

The differences in the ΔR matrix in the biphasic state between the dispersed fraction (Fig. 6 *C*) and the condensed fraction (Fig. 6 *D*) indicate that some intramolecular interactions in FUS NTD are replaced by intermolecular interactions during LLPS. To shed light on this issue, we attempted visualization of interaction potentials at ensemble level (Fig. 7). Note that such visualization is necessarily artificial to some extent, because individual conformers interact with each other rather than superimposed ensembles. Indeed, it is not obvious how the conformers should be superimposed. We aimed for a compact spatial representation that keeps N termini and C termini of individual conformers close to each other. To that end we transformed each conformer into the principal axes system of its inertia tensor, selecting the *x* and *z* axes as the axes with the smallest and largest moment of inertia, respectively. We chose the center of mass as frame origin. Directions of the *x* and *z* axes were selected such that the N terminus had negative *x* and *z* coordinates. For each conformer, we computed an electron density map (39) and constructed an ensemble map by population-weighted summation. Isosurface plots of such maps with the *y* axis upright are shown in Fig. 7.

This representation reveals that, on average, conformers in the ensemble are negatively charged in their N-terminal moiety and positively charged in their C-terminal moiety, with positive charge density being larger. Given the sequence of the NTD (Fig. 1), this finding is not surprising. The 10 positively charged residues are all situated in the RGG1 domain, which features only five negatively charged residues. The QGSY-rich domain contains only two negatively charged residues. Likewise, the cation-*π* interaction potential has its *π*-interaction propensity mostly in the N-terminal moiety (the QGSY-rich domain) and its cationic propensity in the C-terminal moiety (RGG1 domain). The visualization suggests that *π*-cation interaction may be more important than electrostatic interaction for the behavior of FUS NTD.

**Figure 8:**
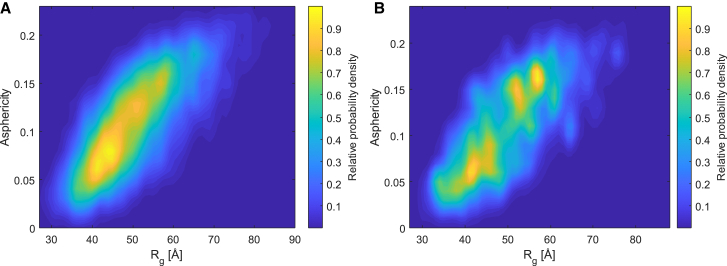
Correlation between asphericity *A* and radius of gyration $R_{g}$ of FUS NTD conformers in (*A*) the unrestrained ensemble and (*B*) the condensed ensemble. Data are represented as smoothed probability density normalized to maximum probability density. To see this figure in color, go online.

The spatial representation in Fig. 7 further indicates that, on average, conformers do not feature spherical symmetry. Such shape anisotropy of random-coil polymers is a well-known phenomenon (70). It can be quantified by the asphericity of the tensor of inertia (37) and has recently been discussed in the context of LLPS of the RGG domain of LAF-1 (30). Ensemble asphericity ⟨A⟩ of the random-flight chain (70) computes to 0.128. Since more extended chains have larger anisotropy, asphericity *A* of individual conformers is correlated to their radius of gyration $R_{g}$ and to their hydrodynamic radius. In Fig. 8, we explore this correlation for the unrestrained and condensed ensemble of FUS NTD. Ensemble asphericities are 0.137 for the unrestrained and 0.135 for the condensed ensemble, close to the expectations for a random coil. The correlation between asphericity and radius of gyration for the condensed ensemble of FUS NTD (Fig. 8 *B*) is surprisingly similar to the one of LAF-1 RGG displayed in the supporting information of (30). We note, however, that in our case the probability density maxima should not be assigned to separate subensembles. Most likely, they arise from statistical noise due to the relatively small number of 2102 conformers in the ensemble, with some having a much lower population than others. For the unrestrained ensemble with 5000 conformers with uniform populations, we observe a rather smooth distribution (Fig. 8 *A*). Altogether, analysis of asphericity supports the view that condensed FUS NTD behaves as a random coil.

**Figure 9:**
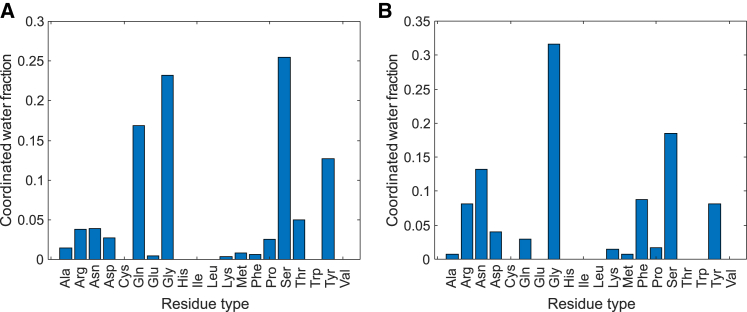
Predicted distribution of hydration water molecules over residue types in (*A*) the condensed-state ensemble of FUS NTD and (*B*) the dispersed-state ensemble of the glycine-rich domain of hnRNP A1. In each case, a sample of 50 conformers was analyzed. Hydration sites were predicted by Accutar (41). The hnRNP A1 ensemble was taken from (42). To see this figure in color, go online.

Finally, we assessed the hydration (71) of FUS NTD conformers from the condensed ensemble using prediction of hydration sites by a neural network algorithm (41). The algorithm predicts that, on average, 773 water molecules hydrate an isolated FUS NTD molecule. Conformation-dependent fluctuations give rise to a standard deviation of 44 coordinated water molecules. The mean number of coordinated water molecules per residue is 3.0 for the QGSY-rich domain and 2.6 for the RGG1 domain. For comparison, for the dispersed ensemble of the RNA-binding protein hnRNP A1 derived from DEER and SAXS restraints (42), the same algorithm predicts per-residue hydration of 1.5 for the folded domain and 2.8 for the glycine-rich domain. Although the latter domain is more compact than FUS NTD, the predicted extent of hydration is similar. In FUS NTD, the most strongly hydrated residue types are, in this order, Ser, Gly, Gln, and Tyr (Fig. 9). Except for Gly, which contributes strongly to hydration by its abundance, all these residue types have been implicated with intermolecular interactions in the condensed state (19,20,72)

## Discussion

**Figure 10:**
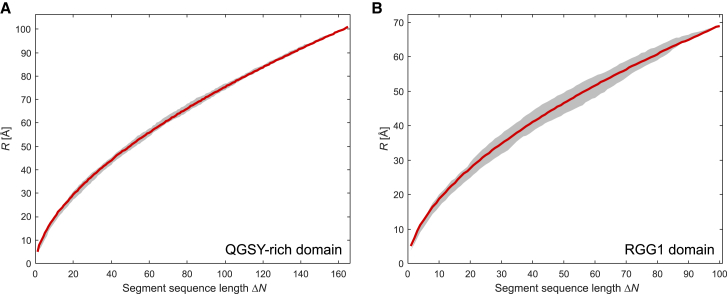
RMS C*α*-to-C*α* distances. RMS C*α*-to-C*α* distances *R* for all sections with sequence separation ΔN (*gray shaded areas*), their averages $\bar{R}\left(\Delta N\right)$ (*solid gray lines*), and random-coil fits according to Eq. 8 (*crimson lines*) in the condensed state for (*A*) the QGSY-rich domain and (*B*) the RGG1 domain. To see this figure in color, go online.

We characterized the conformer distribution of FUS NTD (1–267) in its denatured, dispersed, and condensed state by measuring distance distributions on length scales between about 20 and 80 Å. All experimental datasets were consistent with Gaussian distributions of the label-to-label distance with mean distances ranging from about 31 to 51 Å and standard deviations ranging from about 9 to 16 Å. Taking these distributions as restraints, we derived ensemble models for the three states. All three ensembles are close to random coils in a good solvent. The radius of gyration $R_{g}$ decreases from the denatured state at 3 M urea concentration (58.2 Å) via the dispersed state in a biphasic sample after LLPS (54.0 Å) to the condensed state in the biphasic sample (50.7 Å). The compaction of FUS NTD between the dispersed and condensed state is observed independently for each of the 10 sections (Fig. 2). Indeed, such a decrease in the coil size with increasing concentration is expected for a polymer in a good solvent (73,74), whereas it is at variance with an earlier prediction from coarse-grained molecular dynamics simulations that predicted expansion of the QGSY-rich domain of FUS upon LLPS (72). The asphericity of 0.135 of FUS NTD in the condensed state is close to the value of 0.128 expected for a random coil. Actually, the approximation of the condensed state by a random coil in a good solvent even improves when considering the two domains of FUS NTD individually (Fig. 10). For the QGSY-rich domain, we obtain b=5.13 Å, ν=0.584 and for the RGG1 domain we obtain b=5.05 Å, ν=0.568. Hence, FUS NTD behaves, to a good approximation, as a random-coil block copolymer. The software dScope (75) predicts a slightly higher propensity to LLPS (dScope score) for the QGSY-rich domain (0.873) than for the RGG1 domain (0.756). Notice that the QGSY-rich domain behaves even closer to a random coil in a good solvent than the RGG1 domain.

The condensed state matches an unrestrained ensemble ($R_{g}=51.3$ Å) within experimental precision. This unrestrained ensemble takes into account only residue-specific Ramachandran angle statistics for random coils derived from PDB structures of proteins (76). Specific intramolecular interactions between residues are expected to be averaged in such statistics. Hence, our result suggests that specific intramolecular interactions are negligible for FUS NTD in the condensed state.

Our results further suggest the absence of any significant secondary structure in either of the three states. This is in line with previous conclusions from NMR and coherent anti-Stokes Raman spectroscopy on a QGSY-rich domain construct consisting of residues 1–163 (19,20). For this construct, the overlap concentration was estimated as 285 mg/mL, whereas the macroscopic condensed phase with ≈65% water by volume had a concentration of 477 mg/mL. If we accept bounds for the overlap concentration $C^{*}$ (73)

(9) $$\frac{M}{N_{A}R_{g}^{3}}\geq C^{*}\geq \frac{3M}{4\pi N_{A}R_{g}^{3}}$$

with M=26.3 kDa, the Avogadro constant $N_{A}$ and $R_{g}=51$ Å, chain overlap should occur in our case at concentrations above 78 to 330 mg/mL. From the preparation of bulk phase samples of our construct in earlier work (16), we can estimate the concentration in the condensate as 20 mM, corresponding to 526 mg/mL. This compares to a concentration of 15 mM for FUS 1–214 found by Raman imaging of single droplets (77). Hence, our data suggest that FUS NTD molecules overlap in the condensate, which is again consistent with the behavior of the QGSY-rich domain in isolation as established in (20).

Although the description of FUS NTD as a random-coil polymer in a good solvent is a good approximation, deviations from this approximation are significant, as seen in Figure 2, Figure 5, Figure 6, Figure 10 *B*). Most notably, the RGG1 domain is slightly more compact than the QGSY-rich domain. This effect is observed already in the unrestrained ensemble and can be attributed to the larger glycine content of RGG1. Further differences to random-coil behavior are apparent only in the experimentally restrained ensembles. In particular, section 128–158 is more extended than the other nine measured sections in the denatured and dispersed state. This effect is diminished in the condensed state, where it cannot be safely discerned.

The rather minor change of the conformational ensemble between the dispersed and condensed state and the close approximation of the condensed state by the model of a random-coil block copolymer raise the question of the driving force for LLPS. Regarding the slight compaction of FUS NTD upon LLPS, we note that it is semi-quantitatively predicted within de Gennes’ tricritical theory (73,78,79). This theory is based on virial expansion of monomer interaction, taking into account only the first two virial coefficients. The first coefficient $W_{1}$ is related to excluded volume interaction and the second coefficient $W_{2}$ can be increased by adding sidegroups to the chain and decreased by making the chain more rigid, as remarked upon by de Gennes (78). Higher glycine content thus has two opposing effects on $W_{2}$. No specific sidegroup-sidegroup or sidegroup-backbone interactions need to be invoked to explain the reduction of chain size upon an increase of protein concentration. This phenomenon is generic polymer behavior rather than sequence dependent. We would expect it for any IDR whose residue composition leads to good-solvent conditions. We note that the QGSY-rich domain (19,80) as well as FUS NTD (16) feature a decreasing tendency to LLPS with increasing temperature. This implies that the enthalpic contribution to the driving force for LLPS dominates over the entropic contribution (81).

In the following, we assume phase equilibrium between the dispersed and condensed states. For the QGSY-rich domain, half of the protein molecules have been estimated to exchange between the phases within 1.4 s (20). Protein diffusion inside the droplet is much faster than that (16). Given that in our in vitro experiments liquid droplets are stable for hours, the equilibrium assumption is thus expected to be a good approximation. To separate the contribution of hydrated protein to the free energy change upon LLPS from the one of uncoordinated water, we have analyzed the equilibrium by chemical potentials (see supporting material). For the molar contribution to the free-energy change by the hydrated protein, we find $\Delta \mu_{\text{FUS},\text{LLPS}}=-6.8RT$, where RT is the thermal energy. We estimate the contribution of chain entropy change due to compaction as 0.85RT, almost an order of magnitude smaller and with opposite sign. Hence, the main contribution to $\Delta \mu_{\text{FUS},\text{LLPS}}$ comes from changes in intramolecular and intermolecular interactions, as one also expects from the sensitivity of LLPS to mutations.

For the change of molar chemical potential of noncoordinated water upon LLPS of wild-type FUS NTD, we find an upper bound $\Delta \mu_{H_{2}O,\text{LLPS}}<1.7\cdot 10^{-3}RT$ (see Eq. S7). Given that breaking a single hydrogen bond corresponds to a free-energy difference of 2 kJ/mol in water and 2.6 kJ/mol for the water cage around an argon atom (82), we can conclude that the structure of noncoordinated water in the biomolecular condensate hardly differs from the one in bulk water. LLPS involves a minor increase of the free energy of water in favor of a decrease of free energy of the protein. This finding applies in general, as it directly follows from the moderately lower molar fraction of water and the higher molar fraction of protein in the biomolecular condensate.

In line with previous findings from NMR, molecular dynamics, and sequence analysis (13,19,20,21,72,83,84,85), our results suggest that a multitude of transient, weak interactions between amino acid residues are the driving force for LLPS of FUS NTD. We find no indication for persistent structure, except perhaps for a slight extension of the section 128–158 in the dispersed state. The earlier works have identified hydrogen bonding as the dominating interaction type, with $\pi -sp^{2}$ interactions (86) between Tyr and predominantly Gln also contributing substantially. This picture suggests partial dehydration of the protein upon LLPS, which is consistent with recent findings from Terahertz spectroscopy (47,48).

We note that the presence of folded domains and of the other two RGG domains could modify LLPS behavior for full-length FUS, which is known to undergo LLPS at lower concentrations than the QGSY-rich region on its own (85). A recent publication demonstrated that the protein compacts as it approaches LLPS (87), but the overall conformation of the NTD in this context is not yet known. The fIDPnn disorder prediction shown in Fig. 1 *B* indicates that the last 30 residues of the RGG1 domain may exhibit substantial order in the context of the RNA-recognition motif (285–371), although AlphaFold2 pLDDT is in contrast to this prediction. For the dispersed state of hnRNP A1, we found interaction of the Gly-rich disordered domain with the folded RNA-recognition motifs, suggesting that displacement of the Gly-rich domain from the RNA-recognition motifs by RNA could trigger LLPS (42). Even considering such modifications in LLPS behavior due to the presence of folded domains, our results demonstrate that the basic features of LLPS caused by IDRs of proteins can be understood by established concepts from polymer physics.

## Conclusions

For FUS NTD, consisting of the QGSY-rich domain and the RGG1 domain, we have obtained ensemble models based on distance distribution restraints for the denatured, dispersed, and condensed state. Both in the dispersed and in the condensed state, FUS NTD can be described to a good approximation as a random-coil block copolymer in a good solvent. This explains the slight compaction of FUS NTD upon LLPS that we observed for all 10 sections tested, since random coils in good solvent contract upon an increase of polymer concentration (73,79) as a consequence of de Gennes’ theory (78). The concentration of FUS NTD in the biomolecular condensate exceeds the overlap concentration of the random coil. The QGSY-rich block is slightly more extended and predicted to be slightly more hydrated and have a slightly larger propensity to LLPS than the RGG block. In the condensed state, chain extension is nearly homogeneous along the sequence, in particular for the QGSY-rich domain. In the dispersed state, a section in the range between residues 128 and 158 appears to be slightly more extended. Our results demonstrate that the formation of biomolecular condensates at physiologically relevant protein concentrations is consistent with full disorder and with good solvation of protein domains in both the dispersed and condensed state.

## Author contributions

L.E.H., L.E., F.H.T.A., and G.J. designed the research. M.Y. assisted with the experimental design for EPR. L.E.H. performed and analyzed the EPR and microscopy experiments. L.E.H. and L.E. performed sample preparation and turbidity experiments. L.E.H. carried out ensemble modeling with some software support by G.J. L.E.H. and G.J. analyzed the ensembles. L.E.H. and G.J. wrote the article with input from all other authors.

## Supporting material

Document S1. Figures S1–S13 and Tables S1–S3

Document S2. Article plus supporting material
