# Supplementary material for: Ensemble structure of the N-terminal domain (1–267) of FUS in a biomolecular condensate
Source: Biophys J. 2024 Jan 26;123(5):538–54. doi: 10.1016/j.bpj.2024.01.023 (PMC10938082; doi:10.1016/j.bpj.2024.01.023)
Supplement: Document S1. Figures S1–S13 and Tables S1–S3 [file mmc1.pdf]

Biophysical Journal, Volume *123*

Supplemental information

Ensemble structure of the N-terminal domain (1–267) of FUS in a biomolecular condensate

Laura Esteban-Hofer, Leonidas Emmanouilidis, Maxim Yulikov, Frédéric H.-T. Allain, and Gunnar Jeschke

Supporting Material:

Ensemble Structure of the N-terminal Domain (1-267) of FUS in a Biomolecular Condensate

Laura Esteban-Hofer,[a] Leonidas Emmanouilidis,[b] Maxim Yulikov,[a] Frédéric H.-T. Allain,[b] Gunnar Jeschke*[a]

[a]*ETH Zurich, Department of Chemistry and Applied Biosciences, Vladimir-Prelog-Weg 2, 8093 Zurich, Switzerland* [b]*ETH Zurich, Department of Biology, Hönggerbergring 64, 8093 Zurich, Switzerland*

Supplemental methods

Visualization of electrostatic interaction potential

Semi-quantitative visualization of the electrostatic interaction potential was performed by superimposing electrical fields from point charges of charged residues. Our approach considers the pH value, taken as 7.0 in our visualizations, and the pKa values of amino acid sidegroups for computing effective charges. We localize the charge for Asp at the mean of the OD1 and OD2 atom coordinates, for Glu at the mean of the OE1 and OE2 atom coordinates, for His at the mean of the ND1 and NE1 coordinates, for Lys at the NZ atom coordinate, and for Arg at CZ atom coordinate. At all cubic grid points for which the electron density map is computed, we add the electric fields generated by all point charges, considering charge screening with the Debye length computed from the ionic strength of the buffer, taken here as 150 mM. The mean electric field from all charged residues at the displayed isosurface of the ensemble electron density determines the color. Blue corresponds to positive charge, red to negative charge, and white to neutrality. For good visibility, we set the extrema of the color scale to the largest absolute electric field value encountered at the whole isosurface.

We note that this approach is too crude for computing intermolecular interactions, where a Poisson-Boltzmann approach should be used instead. In our case, visualization provides only an intuitive picture in any case, as the weighted superposition of conformers in their inertia tensor principal axes systems is an artificial construct. Therefore, we prefer the simple approximation for the electric field sketched above and interpret the visualization with due caution.

Visualization of a cation-π interaction potential

Effective charges for Arg and Lys were computed considering their pKa values and the pH value (taken as 7.0). The contribution of Arg was weighted by 0.7088 and the one of Lys by 0.2912, thus taking into account relative interaction propensities found in[1]. Likewise, we assume relative π interaction propensities of -1 for Trp, of -0.5466 for Tyr, and of 0.3821 for Phe. The cation center coordinates were located as for the electrostatic interaction described above, whereas the π center coordinates were located at the centers of the aromatic systems of the respective sidegroups. We assumed decay of the cation-π interaction with the inverse square of the distance. The color scale is between gold (π systems) and blue (cations) with the centre at white. The propensity values corresponding to the extremes of the color scale were taken so as to obtain a similar visualization for cations as in the visualization of the electrostatic interaction potential described above.

We note that this approach can only provide an intuitive picture of the spatial distribution of cation and π interaction propensities with respect to the N and C termini of the protein.

Estimates of protein and water concentration and molar fractions

Biphasic samples were prepared with an initial concentration of 200 μM and 48% of the protein ended up in the condensed phase for wild-type FUS NTD. This puts concentration of the dispersed phase to 104 μM. Water concentration in saline buffer is approximately 55.5 M, which is by a factor of $5.34 \cdot 10^5$ larger than protein concentration. Even considering the hydration layer, we can estimate $x_{\mathrm{H_2O,disp}} \approx 1$ and $x_{\mathrm{FUS,disp}} \approx 1.04 \cdot 10^{-4}/55.5 = 1.9 \cdot 10^{-6}$.

In earlier work, we obtained 40 μL of bulk condensed FUS NTD from 80 μL of 10 mM FUS NTD stock[2]. This suggests an approximate concentration of 20 mM FUS NTD in the condensed phase, similar to the concentration of 15 mM found recently for a construct of FUS consisting of residues 1-214[3]. With the molar mass of 26295 g/mol, this corresponds to 526 mg/mL protein. Assuming a density close to 1 g/mL, we find a water concentration of about 26.9 M, which exceeds protein concentration only by a factor of the order of 1000. In this situation, water in the hydration layer[4] makes up a substantial fraction of total water. The Accutar approach[5] predicted an average of $n_{\mathrm{hydr}} = 773$ hydration water molecules per protein molecule with a standard deviation of 44. This provides a concentration of uncoordinated water of $c_{\mathrm{H_2O,free}} = 26.9\ \mathrm{M} - n_{\mathrm{hydr}} \cdot 20\ \mathrm{mM} = 11.44\ \mathrm{M}$. This value, in turn, leads to a molar fraction of uncoordinated water of $x_{\mathrm{H_2O,cond}} = 0.9983$ and a molar fraction of hydrated FUS NTD of $x_{\mathrm{FUS,cond}} = 1 - x_{\mathrm{H_2O,cond}} = 1.7 \cdot 10^{-3}$, where we neglected molar fractions of other components (salts, HEPES, urea). Note also that water beyond the first hydration shell is affected by the presence of the protein. The discussion in the main text reveals that the latter effect is minor.

Thermodynamics of LLPS

In phase equilibrium, the chemical potential of the protein is the same in both phases and the chemical potential of water is also the same in both phases. The dispersed phase is a highly diluted protein solution that we take as our reference state. For the molar chemical potential of FUS NTD in this phase, we have

$$\mu_{\mathrm{FUS,disp}} \approx \mu^{\circ}_{\mathrm{FUS}} + RT \ln x_{\mathrm{FUS,disp}} \,, \tag{S1}$$

where $\mu^{\circ}_{\mathrm{FUS}}$ is the chemical standard potential of hydrated FUS NTD and $x_{\mathrm{FUS,disp}}$ is the molar fraction of FUS NTD in the dispersed state, which we estimate as $x_{\mathrm{protein}} = 1.9 \cdot 10^{-6}$ (see above). Here, R is the universal gas constant and T the temperature of sample preparation that we take as 295 K.

For the condensed phase, we can write

$$\mu_{\mathrm{FUS,cond}} \approx \mu^{\circ}_{\mathrm{FUS},\infty} + RT \ln x_{\mathrm{FUS,cond}} + \Delta\mu_{\mathrm{FUS,LLPS}} \;, \tag{S2}$$

where $\Delta\mu_{\mathrm{FUS,LLPS}}$ is the change of molar chemical potential of FUS NTD due to change of intramolecular and intermolecular interactions as well as of chain conformation entropy upon transfer from the dispersed to the condensed phase. We estimate $x_{\mathrm{FUS,cond}} \approx 1.7 \cdot 10^{-3}$ (see above). This estimate is based on a concentration of FUS NTD in the biomolecular condensate of 20 mM, similar to the concentration of 15 mM found recently for a construct consisting of residues 1-214[3]. From $\mu_{\mathrm{FUS,disp}} = \mu_{\mathrm{FUS,cond}}$ at phase equilibrium, we then have

$$\Delta\mu_{\mathrm{FUS,LLPS}} \approx RT \ln \frac{x_{\mathrm{FUS,disp}}}{x_{\mathrm{FUS,cond}}} = -6.8RT = -16.5 \text{ kJ/mol} \;. \tag{S3}$$

The negative sign indicates that free energy of the hydrated protein in the condensed state at given concentration is lower than in the dispersed state.

We can make a rough estimate of the contribution of the chain conformation entropy change. To that end, we consider the solved problem of entropic elasticity of a Gaussian chain (θ-solvent conditions). Molar entropy of such a chain as a function of chain size is

$$S(R) = -\frac{3}{2} RT \frac{\vec{R}^2}{Nb^2} + S_0 \;, \tag{S4}$$

where b is the length of a Kuhn segment, N the number of Kuhn segments, and $\vec{R}$ the end-to-end distance vector. We note that $Nb^2 = R_0^2$, where R_0 is the root mean square end-to-end distance of a Gaussian chain with $\nu = 0.5$ in Eq. (10) in the main text. Hence, we substitute

$$S(R) = -\frac{3}{2} RT \frac{\vec{R}^2}{N^{2\nu} b^2} + S_0 \tag{S5}$$

and use $b = 0.51$ Å as well as $\nu = 0.592$ for the dispersed state as our reference state. We computed the ensemble averages for the dispersed and condensed state as population-weighted sums of single-chain contributions, which is in line with the derivation of Eq. (S4). In the difference, S_0 drops out

$$\Delta S(R) = -4.15R - (-3.30R) = -0.85R \;. \tag{S6}$$

The contribution to $\Delta\mu_{\mathrm{FUS,LLPS}}$ is $-T\Delta S(R) = 0.85RT$, which is almost an order of magnitude smaller in magnitude than $\Delta\mu_{\mathrm{FUS,LLPS}}$ and has opposite sign. Although this estimate is rough, it serves to demonstrate that the main contribution to $\Delta\mu_{\mathrm{FUS,LLPS}}$ comes from changes in intramolecular and intermolecular interactions, as one also expects from the sensitivity of LLPS to mutations.

By assuming that hydration of FUS NTD in the condensed states conforms to the prediction of hydration sites for isolated conformers by Accutar[5], we find for the change of molar chemical potential of water upon LLPS of wild-type FUS NTD

$$\Delta\mu_{\mathrm{H_2O,LLPS}} \approx RT \ln \frac{x_{\mathrm{H_2O,disp}}}{x_{\mathrm{H_2O,cond}}} = RT \ln \frac{1}{0.9983} = 1.7 \cdot 10^{-3} RT = 4.1 \text{ J/mol} \;. \tag{S7}$$

This small value is rather an upper bound, since the intermolecular interactions of FUS NTD molecules in the condensed phase will tend to reduce hydration.

Supplemental Figures

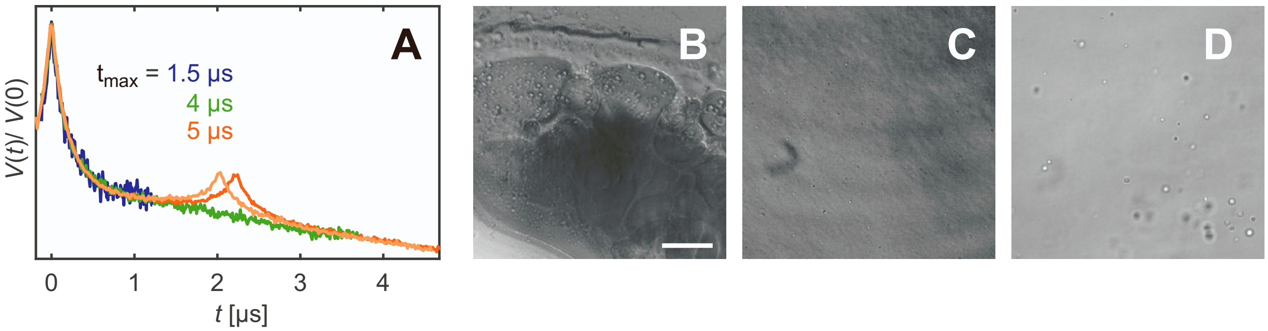

Fig. S1 5-pulse DEER and protein deuteration as potential methods to prolong DEER trace length. (A) The shape of the primary DEER data of biphasic A105C G128C remains unchanged for 4-pulse DEER traces of 1.5 μs (blue) and 4 μs (green). The same dipolar evolution is present in 5-pulse DEER traces of 5 μs with shifted artifact positions by different pulse sequence timing (two shades of orange). Traces were scaled to unit modulation depth for comparison purposes. Protein deuteration leads to unusual phase separation behavior with (B) regions that contain gel-like aggregates and (C) overall smaller (barely visible) droplets than (D) protonated FUS droplets prepared under the same conditions. Scale bar: 20 μm.

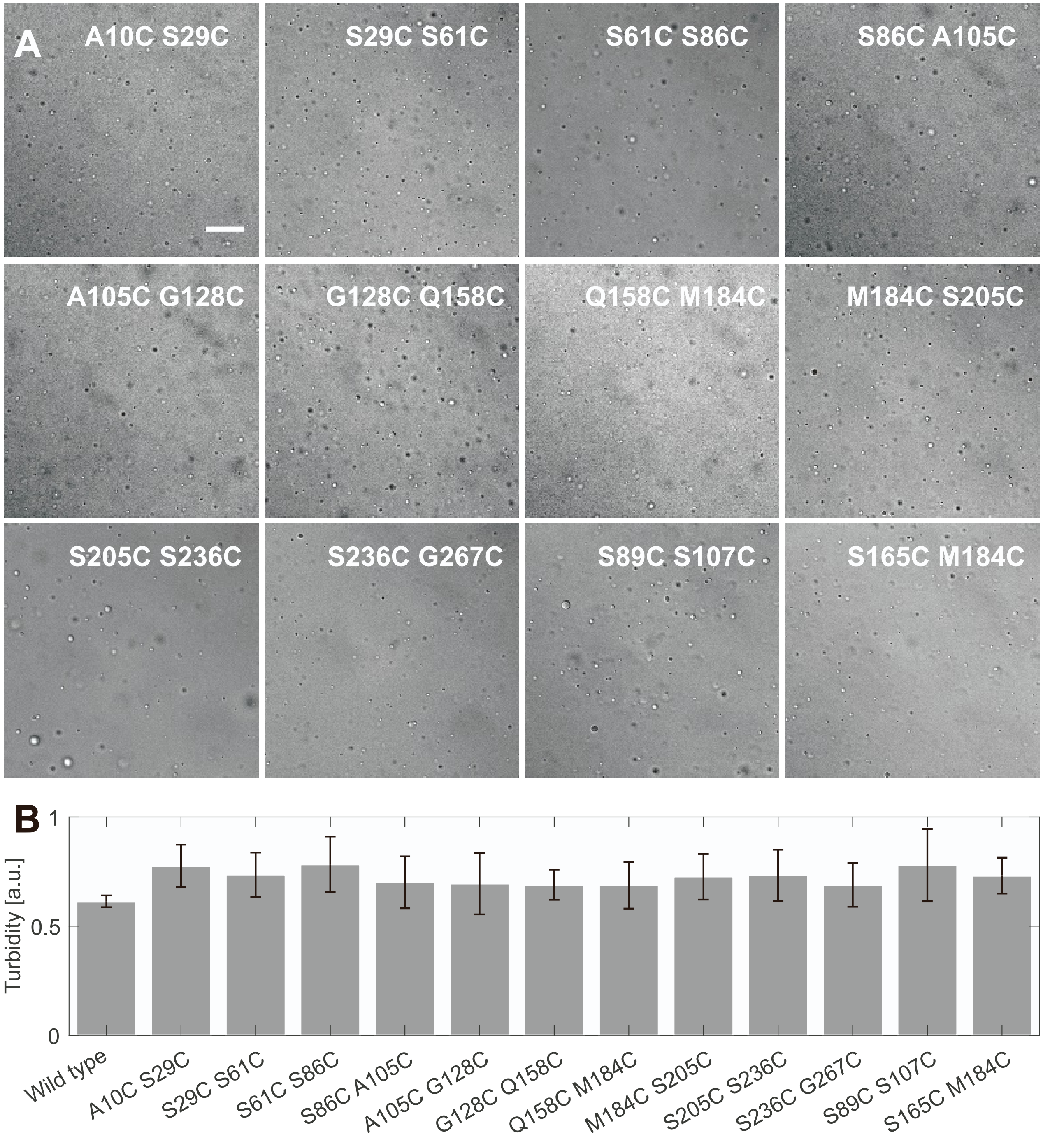

Fig. S2 Characterization of spin-labeled double cysteine mutants. (A) All double cysteine mutants retain the ability to phase separate (scale bar: 20 μm) and (B) have similar turbidity values to the ones of wild-type protein with mean and standard deviation from three independent experiments. The protein concentration employed in all experiments is 50 μM.

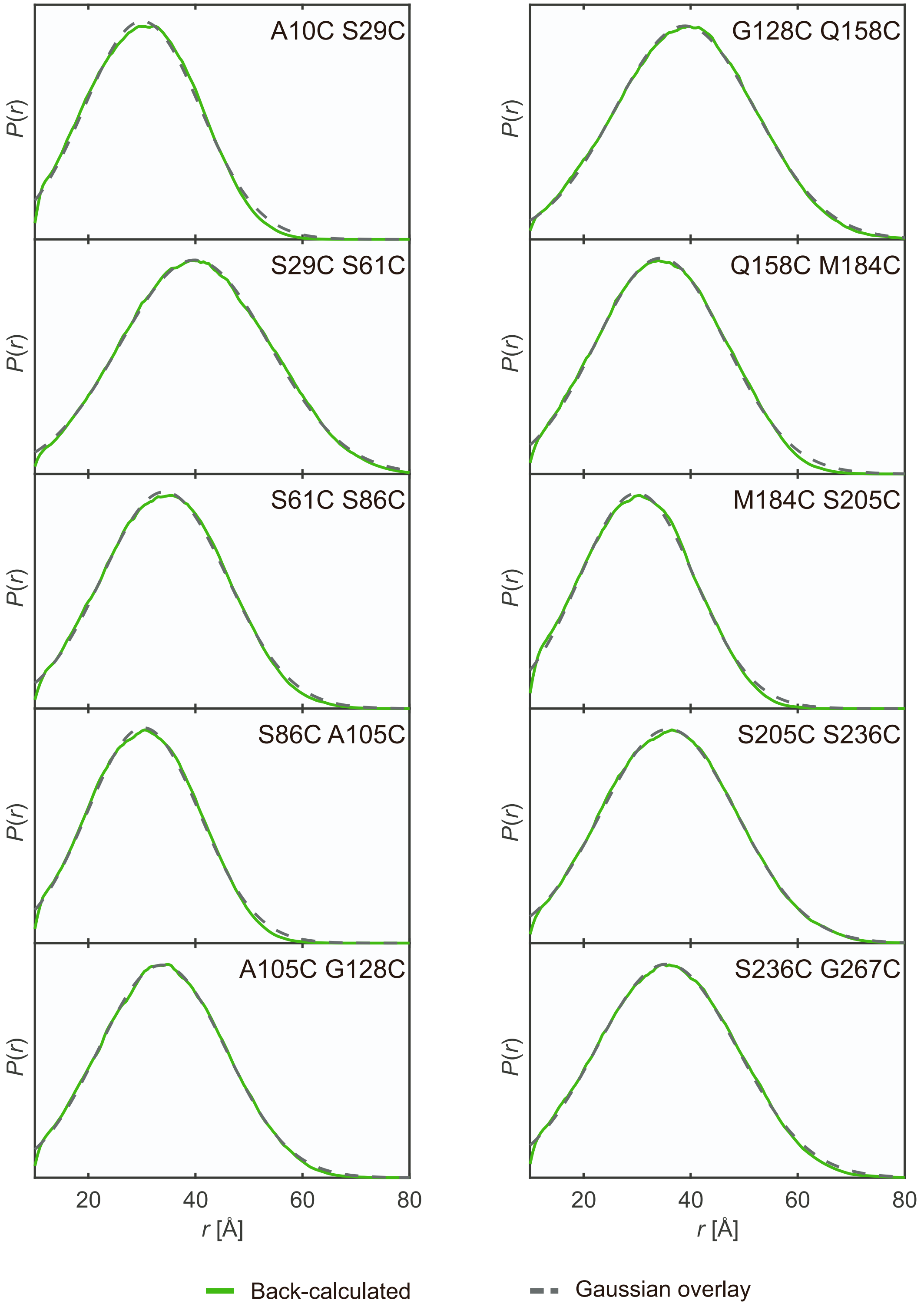

Fig. S3 Back-calculated distance distributions for the unrestrained ensemble. The back-calculated distributions (solid green line) are Gaussian-like and can be overlaid well by Gaussian fits (dashed grey line). Spin-label positions are indicated in the upper right corner of each panel.

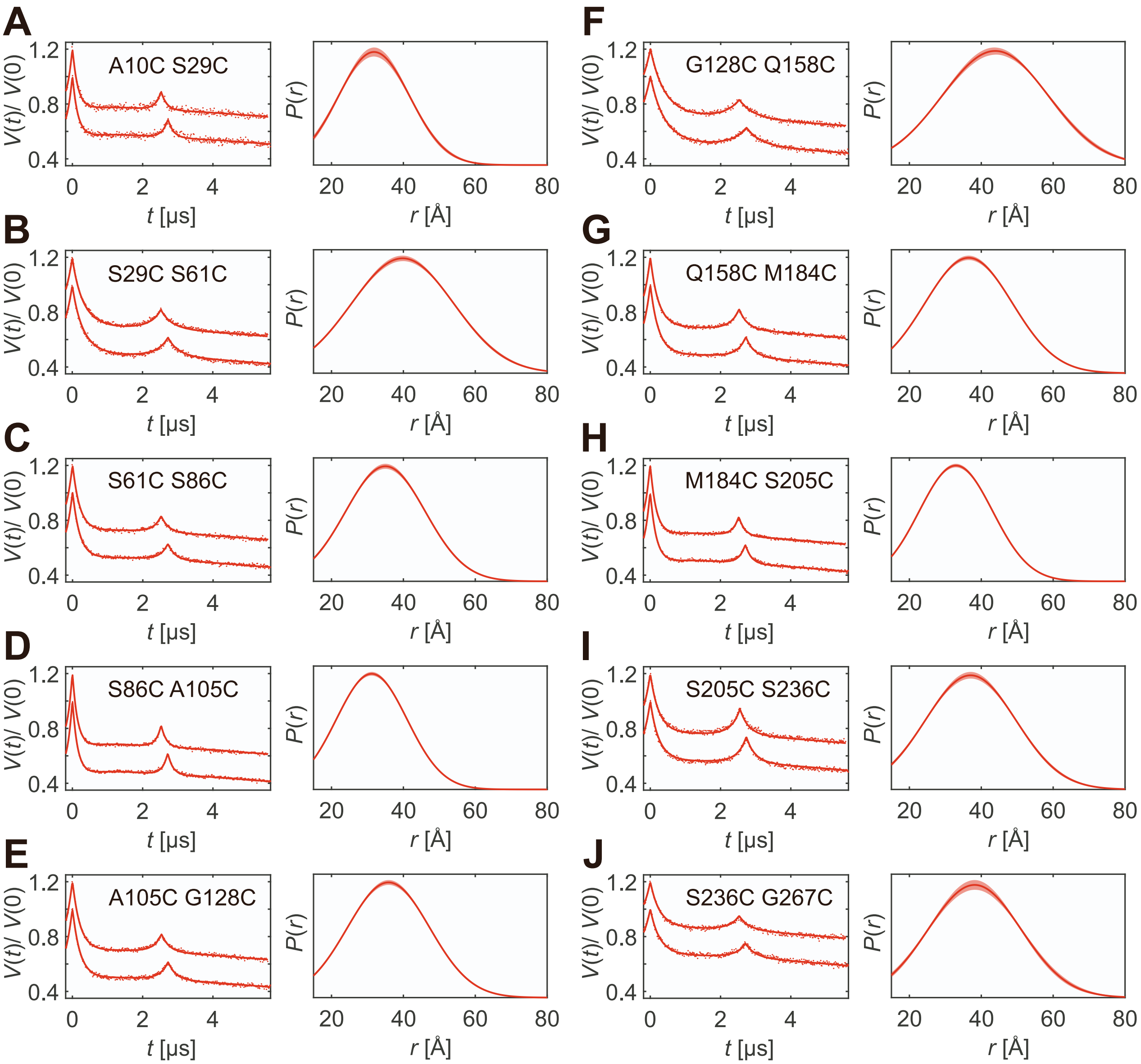

Fig. S4 5-Pulse primary DEER data of monophasic dispersed FUS. Primary DEER data (left) and corresponding distance distribution (right) of (A)-(J) A10C S29C, S29C S61C, S61C S86C, S86C A105C, A105C G128C, G128C Q158C, Q158C M184C, M184C S205C, S205C S236C, and S236C G267C, respectively. Experimental raw data are displayed as dots and Gaussian fits as solid lines with the 95% confidence intervals obtained via 1000 bootstrap samples shown as shaded area. The lower and upper 95% confidence interval of the mean and width of each distance restraint was used to confine the parameter range of the dispersed fraction in the biphasic measurement fits.

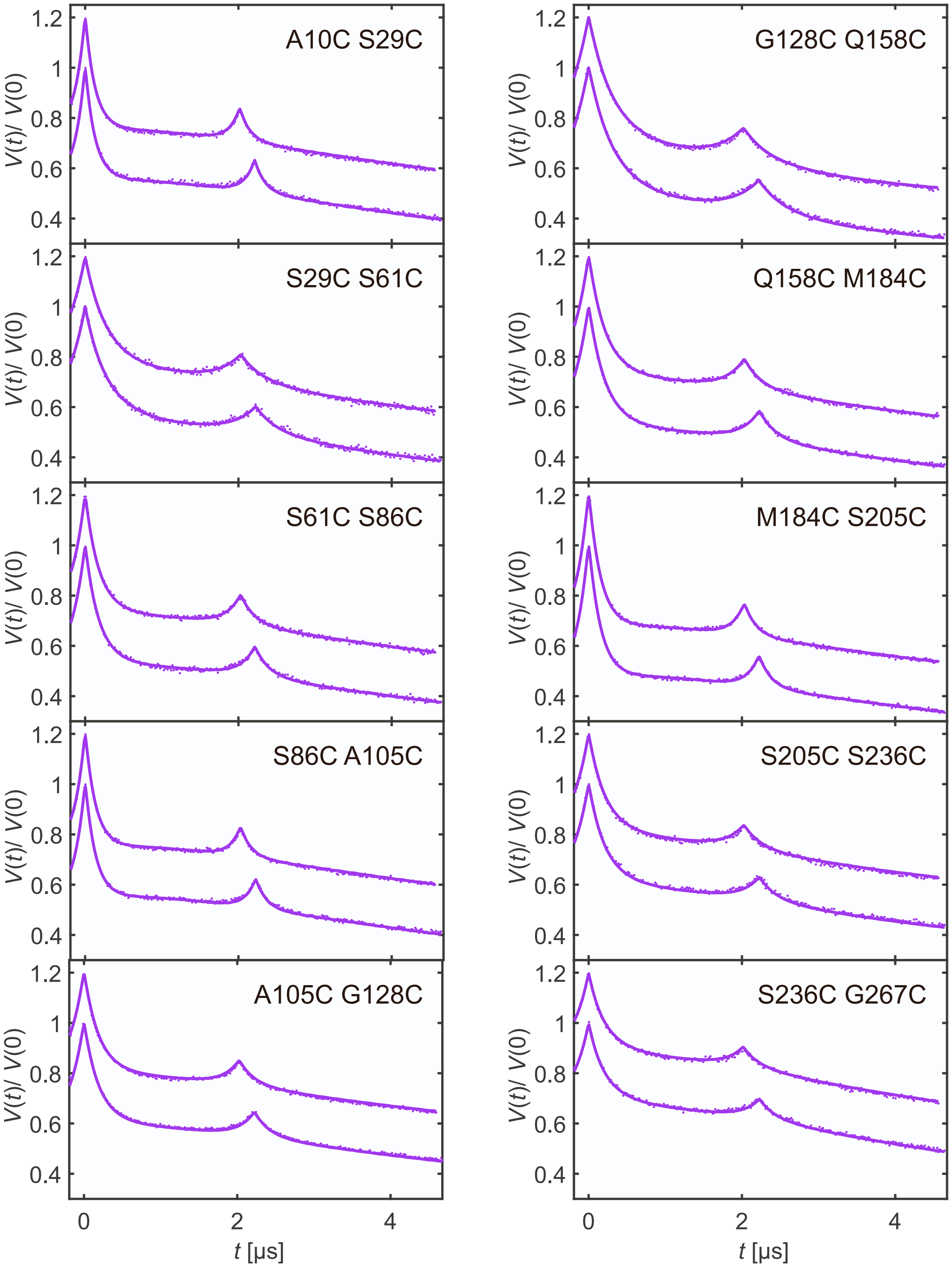

Fig. S5 5-Pulse primary DEER data of biphasic FUS. Two data sets for each mutant were measured with different pulse sequence timings. Spin-label positions are indicated in the upper right corner of each panel. Raw data are shown as violet dots, and the fits as violet lines with the 95% confidence intervals obtained via 1000 bootstrap samples shown as shaded area.

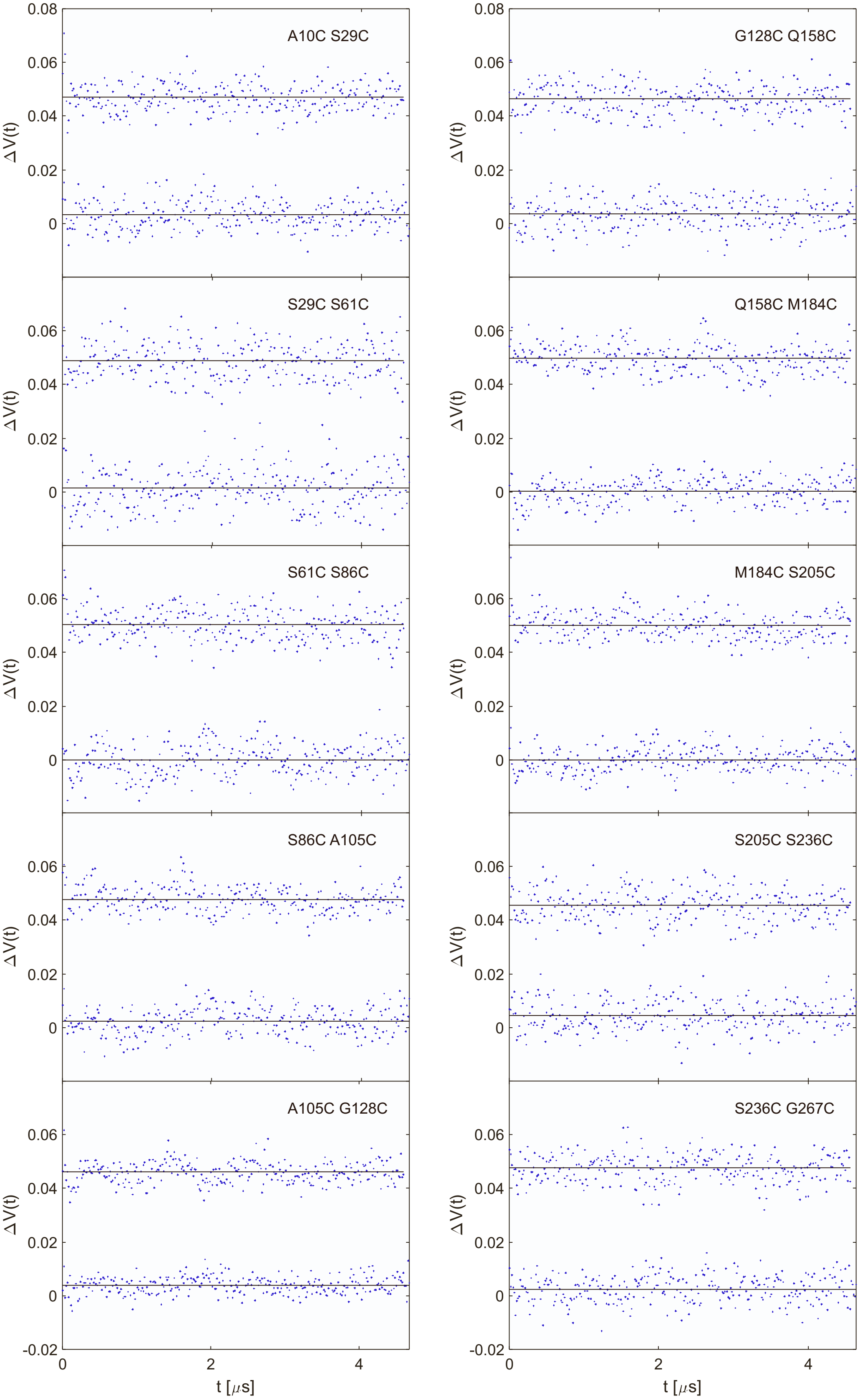

Fig. S6 Fit residuals for 5-pulse DEER data of biphasic FUS. Two data sets for each mutant were measured with different pulse sequence timings. Spin-label positions are indicated in the upper right corner of each panel. Grey horizontal lines denote the mean value of the residual.

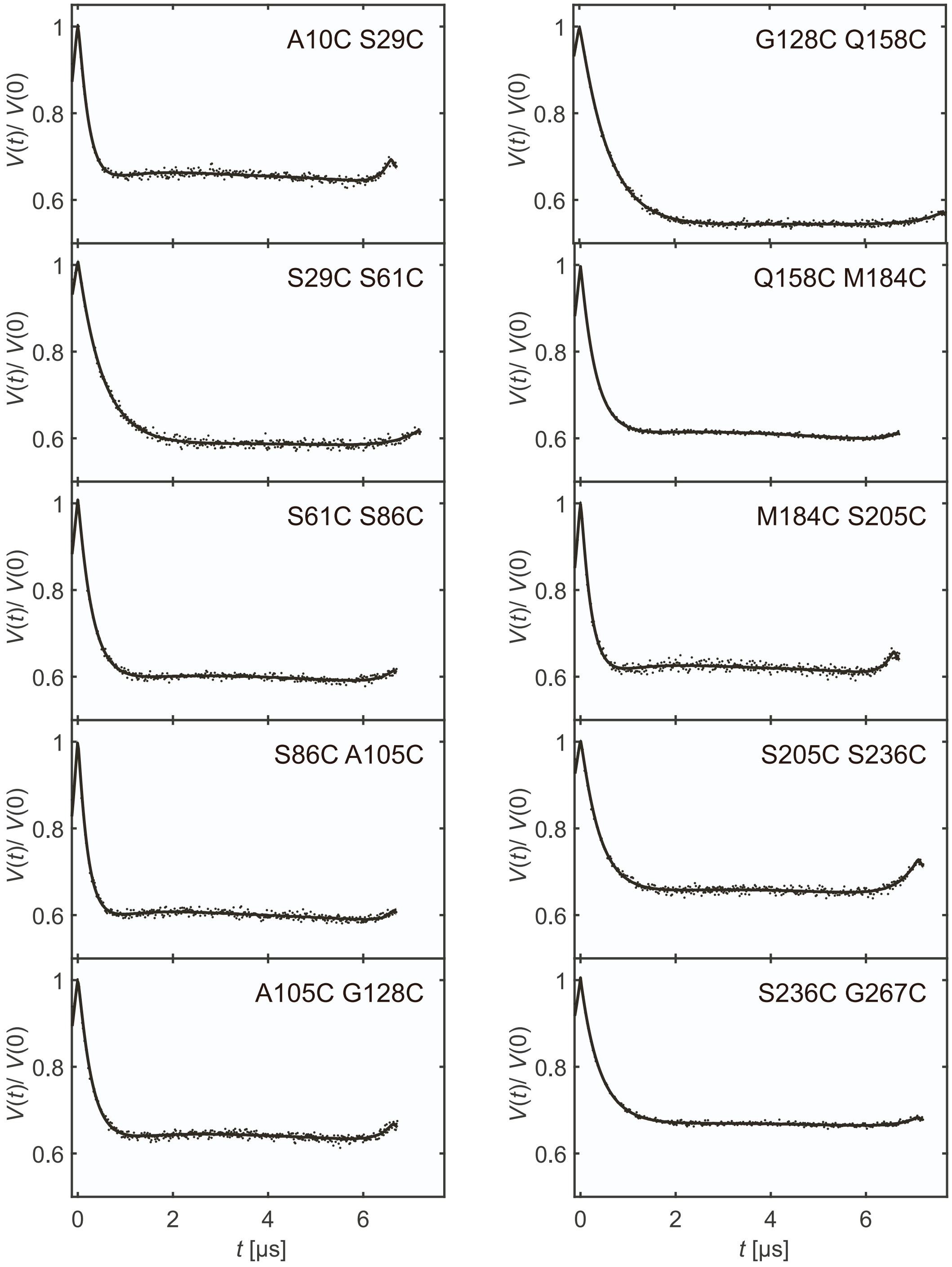

Fig. S7 Primary DEER data of FUS NTD under denaturing conditions. Spin-label positions are indicated in the upper right corner of each panel. Raw data are shown as dots, and Gaussian fits as solid lines with the 95% confidence intervals obtained via 1000 bootstrap samples shown as shaded area.

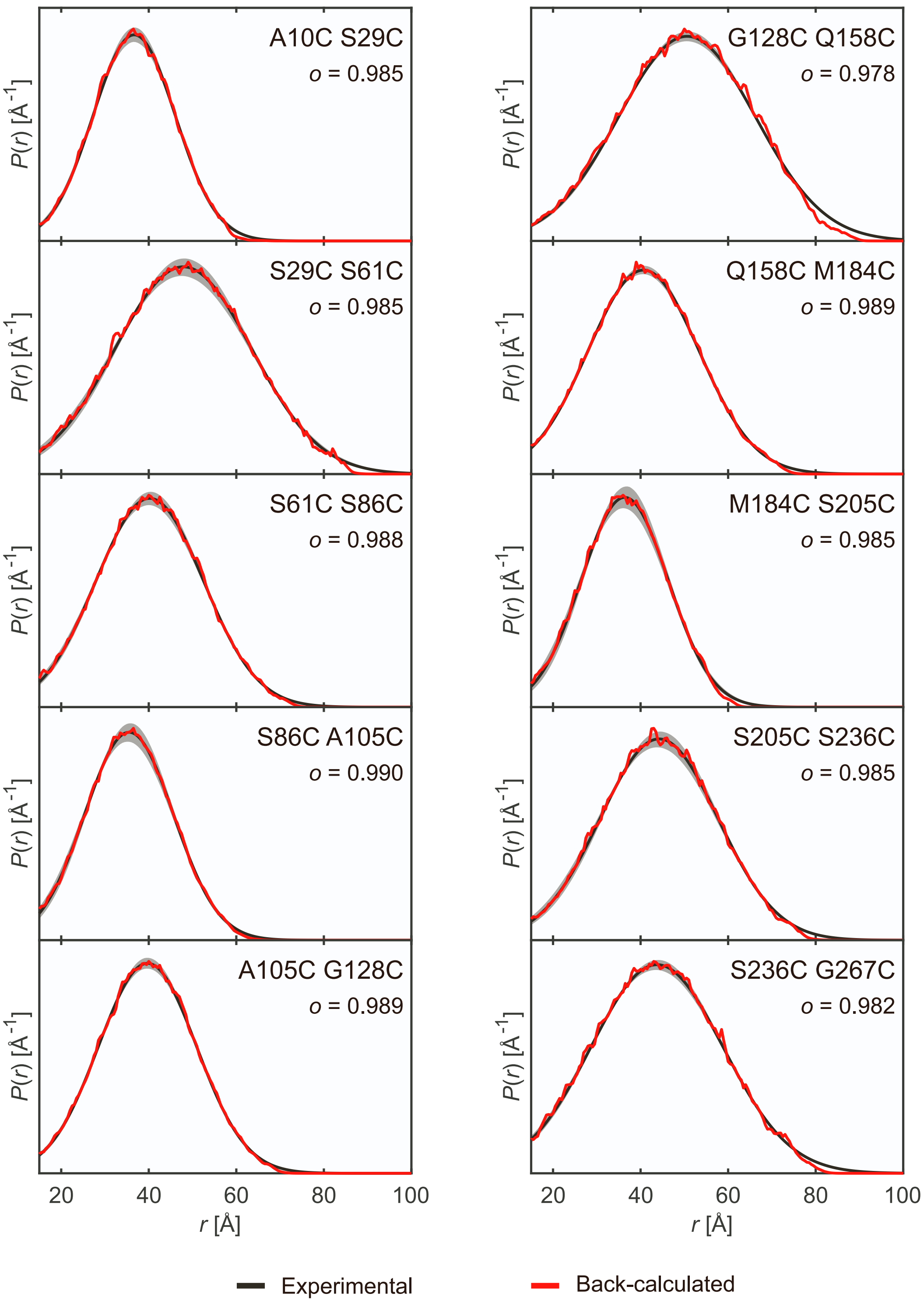

Fig. S8 Restraint fulfilment of the denatured ensemble. Back-calculated distance distributions (red) and experimental restraints (black, with shaded area for the 95% confidence interval obtained by bootstrapping with 1000 samples) are in virtually perfect agreement with each other. Spin-label positions are indicated in the upper right corner of each panel. The overlap o between the experimental and back-calculated distribution is given below.

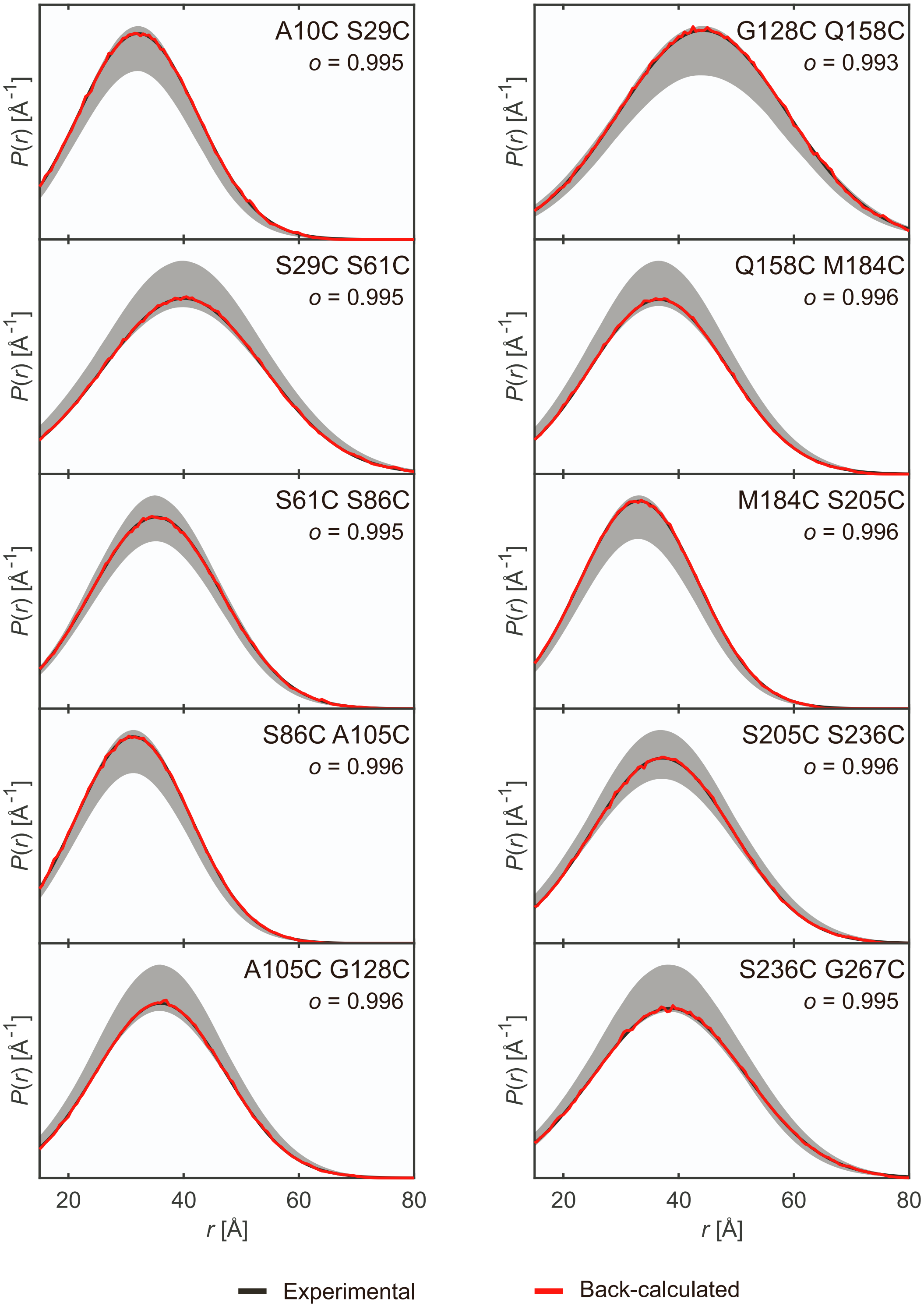

Fig. S9 Restraint fulfilment of the dispersed ensemble. Back-calculated distance distributions (red) and experimental restraints (black, with shaded area for the 95% confidence interval obtained by bootstrapping with 1000 samples) are in perfect agreement with each other. Spin-label positions are indicated in the upper right corner of each panel. The overlap o between the experimental and back-calculated distribution is given below.

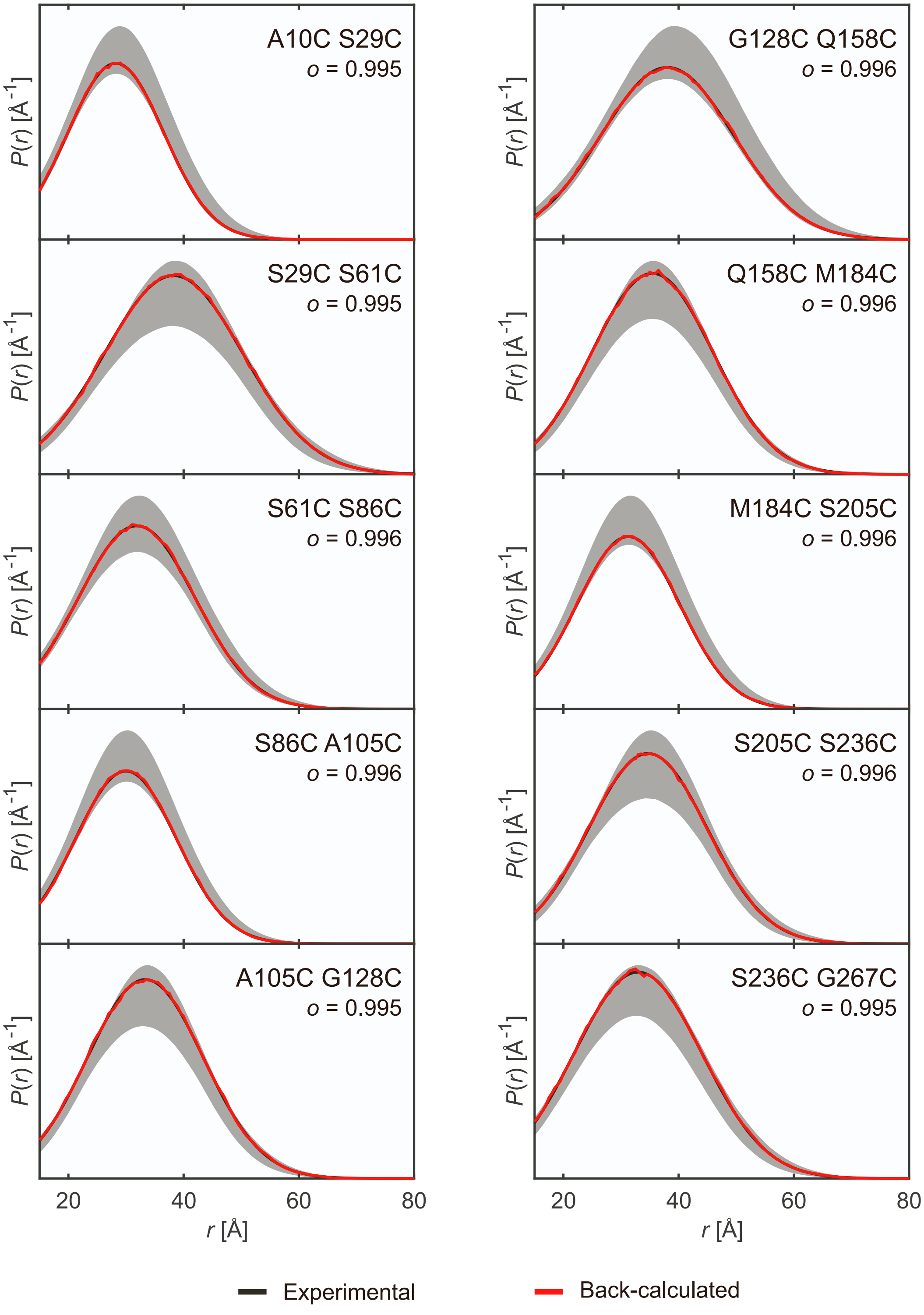

Fig. S10 Restraint fulfilment of the condensed ensemble. Back-calculated distance distributions (red) and experimental restraints (black, with shaded area for the 95% confidence interval obtained by bootstrapping with 1000 samples) are in perfect agreement with each other. Spin-label positions are indicated in the upper right corner of each panel. The overlap o between the experimental and back-calculated distribution is given below.

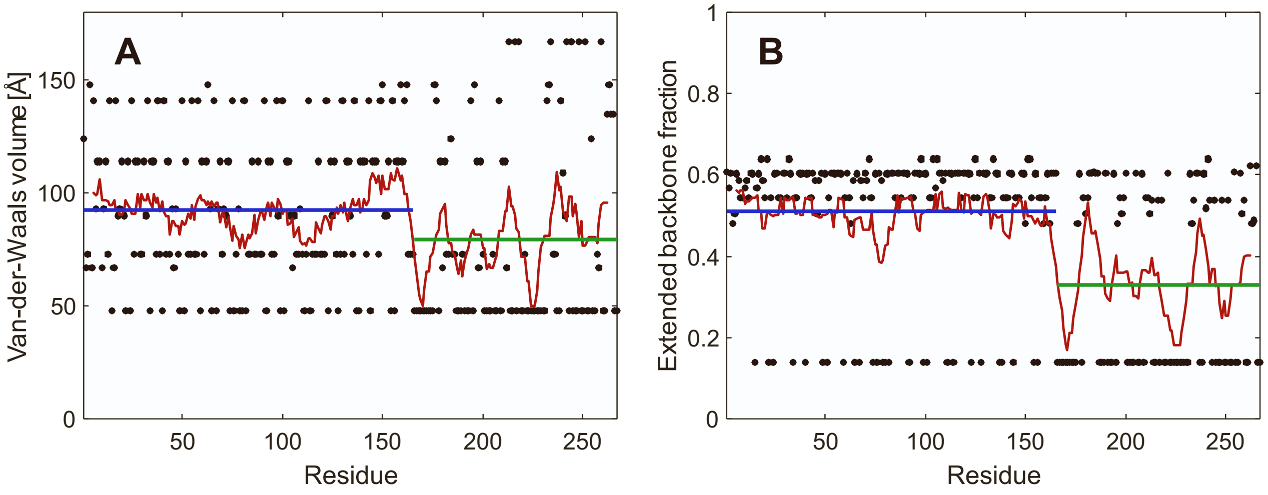

Fig. S11 Van-der-Waals volume of residues (A) and fraction of extended conformations (B) in FUS NTD. Black dots denote individual residues, crimson lines a moving average over a window of 11 residues, blue lines the mean for the QGSY-rich domain (residues 1-165), and green lines the mean for the RGG1 domain (residues 166-267). The fraction of extended conformations is defined as the population in the range $\phi = (-180^\circ, -45^\circ)$, $\psi = (45^\circ, 180^\circ)$ in residue-specific Ramachandran plots.

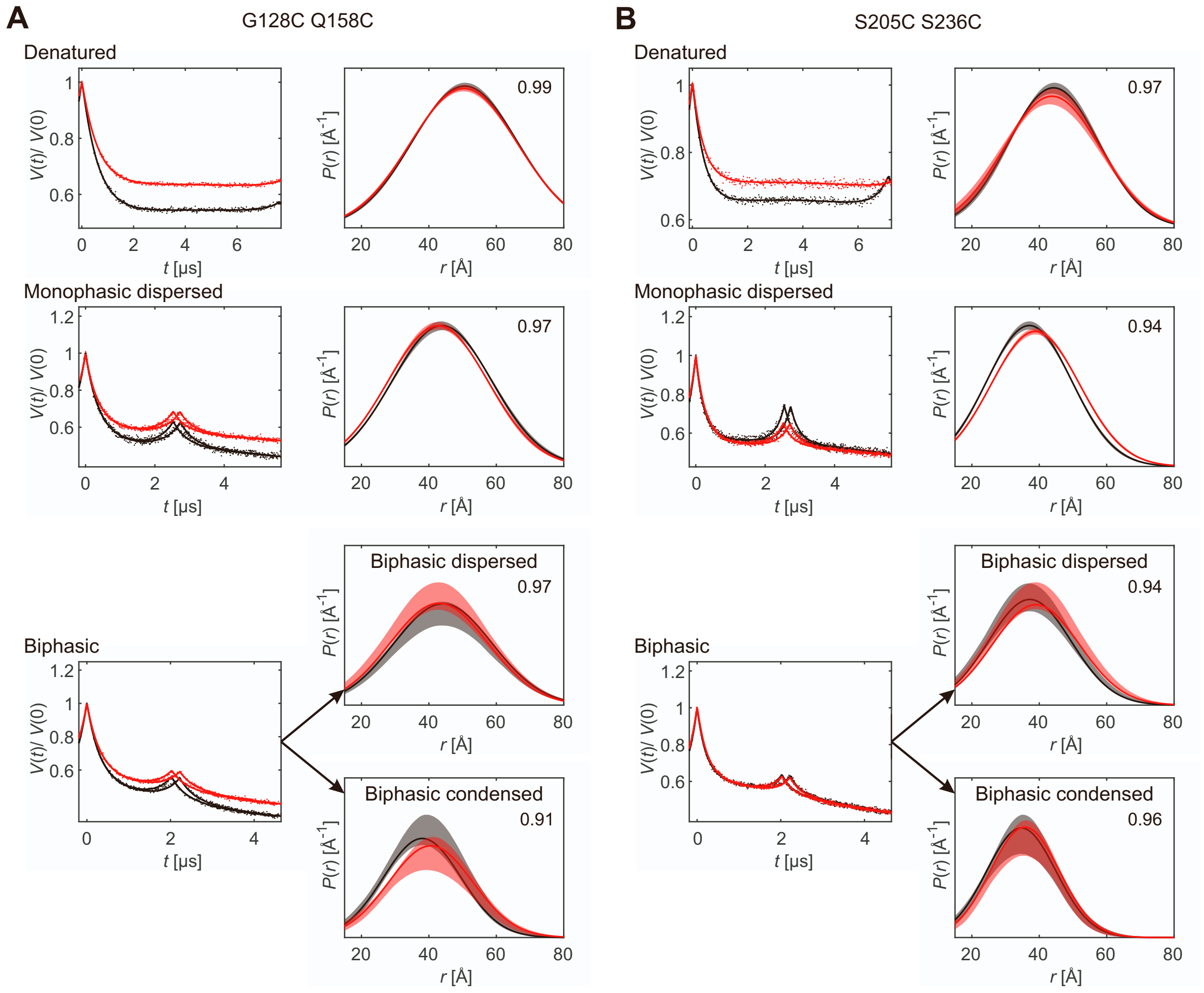

Fig. S12 Reproducibility of DEER measurements for mutants (A) G128C Q158C and (B) S205C S236C. Comparison of original (black) and reproduced (red) primary DEER data (left, raw data are shown as dots, and fits as solid lines) and corresponding distance distributions (right) in the denatured, monophasic dispersed, and biphasic states. Shaded areas correspond to the 95% confidence intervals. The overlap value between the original and reproduced distance distribution is displayed in the upper right corner of each panel. Gaussian fit parameters are reported in Table S3

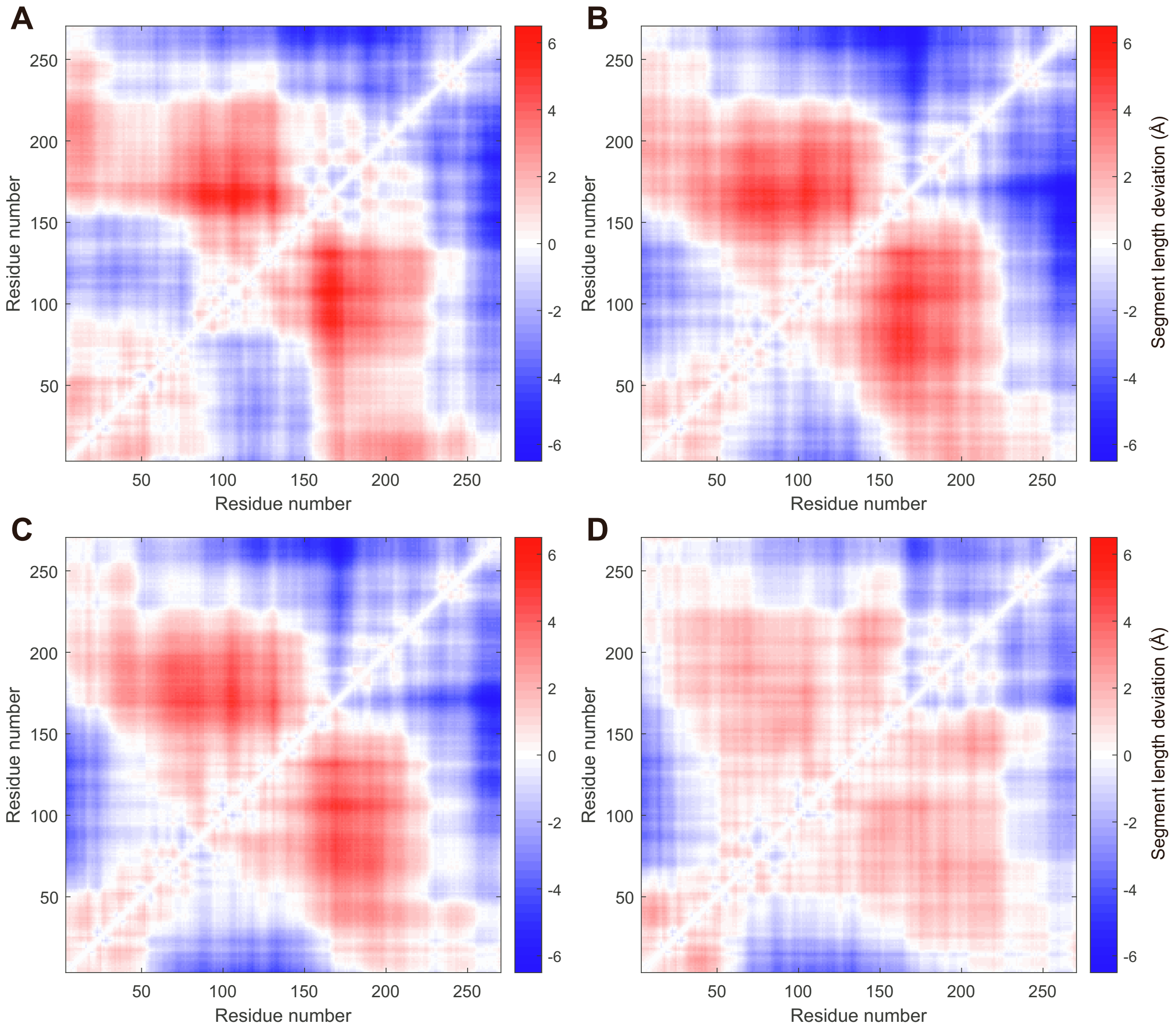

Fig. S13 Section-length deviation ΔR from the average RMS Cα-Cα distance for a given sequence separation ΔN for the dispersed state. The section-length deviations are shown for (A) the main ensemble reported in the main text (except for a slight change in color scale, same as Figure 6C), (B) an ensemble obtained with the same restraints as the main ensemble by reweighting of the unrestrained ensemble, (C) an ensemble obtained by reweighting of the unrestrained ensemble with the restraints for sections 128-158 and 205-236 by restraints obtained in technical repeats, and (D) an ensemble obtained by reweighting of the unrestrained ensemble with the restraint for section 128-158 skipped and all other restraints being the same as for the main ensemble. Each subplot is visualized with the same color axis.

Supplemental Table

Table S1 Parameter range employed for the analysis of the biphasic DEER measurements. The parameter range of the mean $\langle r \rangle_{\mathsf{disp}}$ and full width at half maximum Γ_{disp} of the biphasic dispersed state were constrained to the lower and upper 95% confidence interval (indexed with "low" and "up", respectively) of the monophasic dispersed state measured at 0.6 M urea concentration. We note that $\Gamma = 2\sqrt{2\ln 2}\sigma$ is related to the standard deviation σ only by a constant factor. Hence, restraining and fitting Γ is equivalent to restraining and fitting σ. $T_{0,2}^{(1)}$ and $T_{0,2}^{(2)}$ are the refocusing times of the additional modulated dipolar pathway of the first and second 5-pulse DEER trace, respectively. The parameter η corresponds to the fraction of protein in the dispersed phase and was constrained to $\pm$10% of the fraction determined by DOSY experiments f_{DOSY}.

	Lower	Upper
$\langle r \rangle_{\mathrm{disp}}$ [Å]	$\langle r \rangle_{\mathrm{low}}$	$\langle r \rangle_{\mathrm{up}}$
Γ_{disp} [Å]	Γ_{low}	Γ_{up}
k_{disp} [μs^{-1}]	0	0.09
$\langle r \rangle_{\mathrm{cond}}$ [Å]	10	80
Γ_{cond} [Å]	2	50
k_{cond} [$\mu s^{-d/3}$]	0	1
d_{cond}	2	4
$T_{0,2}^{(1)}$ [μs]	2.20	2.25
$T_{0,2}^{(2)}$ [μs]	2.00	2.05
λ_0	0	1
λ_1	0	1
λ_2	0	1
η	$0.9(1 - f_{\mathrm{DOSY}})$	$1.1(1 - f_{\mathrm{DOSY}})$

Table S2 Gaussian fit parameters (mean distances $\langle r \rangle$ and standard deviations σ) of the DEER measurements on sections of FUS NTD. The lower and upper bounds of the bootstrapped 95% confidence intervals are given in brackets next to the fitted values. The fit parameters of the denatured, biphasic dispersed, and biphasic condensed conditions were employed for ensemble modelling. Data for double mutants A10C S29C and A105C G128C have been taken from[2].

		Dispersed fraction		Condensed fraction	
Mutant	Condition	$\langle r \rangle$ [Å]	σ [Å]	$\langle r \rangle$ [Å]	σ [Å]
A10C S29C	Denatured	36.7 [36.4, 36.9]	9.4 [9.1, 9.8]	-	-
	Monophasic	31.8 [31.5, 32.1]	9.9 [9.6, 10.4]	-	-
	Biphasic	32.1 [31.5, 32.1]	10.4 [9.6, 10.4]	28.2 [28.0, 29.3]	8.3 [8.0, 9.3]
	Bulk	-	-	29.7 [29.7, 29.7]	10.9 [10.9, 10.9]
S29C S61C	Denatured	47.9 [47.3, 48.4]	15.6 [14.9, 16.3]	-	-
	Monophasic	39.8 [39.6, 40.0]	14.2 [13.9, 14.6]	-	-
	Biphasic	40.0 [39.6, 40.0]	13.9 [13.9, 14.6]	38.3 [37.3, 39.0]	12.1 [11.0, 13.5]
S61C S86C	Denatured	40.2 [39.9, 40.6]	12.3 [11.9, 12.7]	-	-
	Monophasic	35.0 [34.8, 35.2]	11.1 [10.9, 11.3]	-	-
	Biphasic	35.2 [34.8, 35.2]	11.3 [10.9, 11.3]	31.9 [31.5, 32.7]	10.1 [9.7, 11.2]
S86C A105C	Denatured	35.5 [35.0, 35.9]	10.1 [9.7, 10.6]	-	-
	Monophasic	31.2 [31.1, 31.3]	9.8 [9.7, 10.0]	-	-
	Biphasic	31.3 [31.1, 31.3]	10.0 [9.7, 10.0]	30.0 [29.8, 30.5]	8.8 [8.5, 9.6]
A105C G128C	Denatured	39.7 [39.5, 39.9]	11.2 [10.9, 11.5]	-	-
	Monophasic	35.9 [35.7, 36.0]	11.5 [11.2, 11.7]	-	-
	Biphasic	36.0 [35.7, 36.0]	11.2 [11.2, 11.7]	33.4 [32.6, 33.9]	10.1 [9.2, 10.9]
	Bulk	-	-	31.6 [31.6, 31.6]	11.7 [11.7, 11.7]
G128C Q158C	Denatured	50.6 [50.3, 50.9]	15.6 [15.3, 16.0]	-	-
	Monophasic	43.9 [43.6, 44.2]	14.8 [14.4, 15.3]	-	-
	Biphasic	44.2 [43.6, 44.2]	14.7 [14.4, 15.3]	38.0 [37.4, 39.8]	11.5 [11.0, 12.6]
Q158C M184C	Denatured	40.7 [40.5, 40.9]	12.6 [12.4, 12.9]	-	-
	Monophasic	36.5 [36.4, 36.6]	12.2 [12.0, 12.4]	-	-
	Biphasic	36.6 [36.4, 36.6]	12.0 [12.0, 12.4]	35.4 [35.0, 35.8]	10.5 [9.7, 11.2]
M184C S205C	Denatured	36.4 [35.8, 36.9]	9.9 [9.4, 10.5]	-	-
	Monophasic	32.9 [32.8, 33.1]	10.2 [10.1, 10.4]	-	-
	Biphasic	33.1 [32.8, 33.1]	10.4 [10.1, 10.4]	31.3 [31.1, 31.9]	8.9 [8.7, 9.7]
S205C S236C	Denatured	44.2 [43.6, 44.8]	13.4 [13.0, 14.0]	-	-
	Monophasic	37.0 [36.8, 37.2]	12.6 [12.2, 13.0]	-	-
	Biphasic	37.2 [36.8, 37.2]	12.2 [12.2, 13.0]	34.7 [34.0, 35.4]	10.3 [9.5, 11.6]
S236C G267C	Denatured	43.8 [43.4, 44.1]	15.1 [14.7, 15.5]	-	-
	Monophasic	38.1 [37.8, 38.5]	12.9 [12.4, 13.5]	-	-
	Biphasic	38.5 [37.8, 38.5]	13.2 [12.4, 13.5]	33.0 [32.0, 33.7]	11.1 [10.3, 12.2]

Table S3 Reproducibility of Gaussian fit parameters (mean distances $\langle r \rangle$ and standard deviations σ) of the DEER measurements on selected sections of FUS NTD. The selected mutants were measured twice; S1 as shown in Table S2 and employed for ensemble modelling, and the reproduced sample S2.

			Dispersed fraction		Condensed fraction	
Mutant	Condition	Sample	$\langle r \rangle$ [Å]	σ [Å]	$\langle r \rangle$ [Å]	σ [Å]
G128C Q158C	Denatured	S1	50.6 [50.3, 50.9]	15.6 [15.3, 16.0]	-	-
		S2	50.4 [50.1, 50.6]	15.9 [15.5, 16.2]	-	-
	Monophasic	S1	43.9 [43.6, 44.2]	14.8 [14.4, 15.3]	-	-
		S2	42.7 [42.4, 43.0]	14.8 [14.4, 15.2]	-	-
	Biphasic	S1	44.2 [43.6, 44.2]	14.7 [14.4, 15.3]	38.0 [37.4, 39.8]	11.5 [11.0, 12.6]
		S2	43.0 [42.4, 43.0]	14.6 [14.4, 15.2]	40.6 [38.8, 41.7]	12.4 [11.4, 14.1]
S205C S236C	Denatured	S1	44.2 [43.6, 44.8]	13.4 [13.0, 14.0]	-	-
		S2	43.8 [42.8, 44.6]	14.3 [13.5, 15.3]	-	-
	Monophasic	S1	37.0 [36.8, 37.2]	12.6 [12.2, 13.0]	-	-
		S2	38.8 [38.7, 39.0]	13.2 [12.9, 13.5]	-	-
	Biphasic	S1	37.2 [36.8, 37.2]	12.2 [12.2, 13.0]	34.7 [34.0, 35.4]	10.3 [9.5, 11.6]
		S2	39.0 [38.7, 39.0]	12.9 [12.9, 13.5]	35.7 [34.8, 36.3]	10.2 [9.2, 11.2]

Supporting References

1 J. P. Gallivan and D. A. Dougherty, *Proc. Natl. Acad. Sci. U.S.A.*, 1999, **96**, 9459–9464.

2 L. Emmanouilidis, L. Esteban-Hofer, F. F. Damberger, T. de Vries, C. K. X. Nguyen, L. F. Ibáñez, S. Mergenthal, E. Klotzsch, M. Yulikov, G. Jeschke and F. H.-T. Allain, *Nat. Chem. Biol.*, 2021, **17**, 608–614.

3 K. Yokosawa, S. Kajimoto, D. Shibata, K. Kuroi, T. Konno and T. Nakabayashi, *J. Phys. Chem. Lett.*, 2022, **13**, 5692–5697.

4 J. J. Virtanen, L. Makowski, T. R. Sosnick and K. F. Freed, *Biophys. J.*, 2010, **99**, 1611–1619.

5 P. Huang, H. Xing, X. Zou, Q. Han, K. Liu, X. Sun, J. Wu and J. Fan, *Front. Mol. Biosci.*, 2021, **8**, 756075.
