# Supplementary material for: Ensemble structure of the N-terminal domain (1–267) of FUS in a biomolecular condensate
Source: Biophys J. 2024 Jan 26;123(5):538–54. doi: 10.1016/j.bpj.2024.01.023 (PMC10938082; doi:10.1016/j.bpj.2024.01.023)
Supplement: Document S2. Article plus supporting material [file mmc2.pdf]

Biophysical Journal
Article

Biophysical Society

Ensemble structure of the N-terminal domain (1–267) of FUS in a biomolecular condensate

Laura Esteban-Hofer,[1] Leonidas Emmanouilidis,[2] Maxim Yulikov,[1] Frédéric H.-T. Allain,[2] and Gunnar Jeschke[1,*]
[1]ETH Zurich, Department of Chemistry and Applied Biosciences, Zurich, Switzerland and [2]ETH Zurich, Department of Biology, Zurich, Switzerland

ABSTRACT Solutions of some proteins phase separate into a condensed state of high protein concentration and a dispersed state of low concentration. Such behavior is observed in living cells for a number of RNA-binding proteins that feature intrinsically disordered domains. It is relevant for cell function via the formation of membraneless organelles and transcriptional condensates. On a basic level, the process can be studied in vitro on protein domains that are necessary and sufficient for liquid-liquid phase separation (LLPS). We have performed distance distribution measurements by electron paramagnetic resonance for 13 sections in an N-terminal domain (NTD) construct of the protein fused in sarcoma (FUS), consisting of the QGSY-rich domain and the RGG1 domain, in the denatured, dispersed, and condensed state. Using 10 distance distribution restraints for ensemble modeling and three such restraints for model validation, we have found that FUS NTD behaves as a random-coil polymer under good-solvent conditions in both the dispersed and condensed state. Conformation distribution in the biomolecular condensate is virtually indistinguishable from the one in an unrestrained ensemble, with the latter one being based on only residue-specific Ramachandran angle distributions. Over its whole length, FUS NTD is slightly more compact in the condensed than in the dispersed state, which is in line with the theory for random coils in good solvent proposed by de Gennes, Daoud, and Jannink. The estimated concentration in the condensate exceeds the overlap concentration resulting from this theory. The QGSY-rich domain is slightly more extended, slightly more hydrated, and has slightly higher propensity for LLPS than the RGG1 domain. Our results support previous suggestions that LLPS of FUS is driven by multiple transient nonspecific hydrogen bonding and $\pi - sp^2$ interactions.

SIGNIFICANCE Liquid-liquid phase separation (LLPS) underlies the formation of membraneless organelles under normal and stress conditions. The protein fused in sarcoma (FUS) is involved in the formation of stress granules, whose aberrant aging is related to amyotrophic lateral sclerosis and frontotemporal dementia. By deriving an ensemble model for the N-terminal domain (NTD), which undergoes LLPS in vitro, we obtain insight into the polymer solvation regime and chain extension of FUS NTD in a biomolecular condensate. Our results rationalize the slight compaction of FUS NTD upon LLPS and contribute to the understanding of the thermodynamics of biomolecular condensates.

INTRODUCTION

Persistent as well as transient membraneless organelles are biomolecular condensates that arise from liquid-liquid phase separation (LLPS) (1–4). Their formation and integrity depend on multi-valent interactions between proteins and RNA (1,5), with charge-charge, charge-sp^2, and π-π interactions playing an important role (6). Often, intrinsically disordered regions (IDRs) of proteins are necessary or sufficient for driving LLPS (6–8). The importance of IDRs and of LLPS for the formation of biomolecular condensates establishes a strong link between polymer physics and biophysics (9). Biomolecular condensates, such as stress granules in cells, are not in a thermodynamic equilibrium state and may thus age. Such aging can lead to fibril formation of prion-like IDRs and to solidification (10), which is related to neurodegenerative diseases (11,12).

LLPS can be recapitulated in vitro through the formation of liquid droplets (13). Such experiments provide access to the basic polymer physics that underlies the formation of biomolecular condensates (14), although they may not necessarily capture the rich structure of inhomogeneous membraneless organelles (11). Although structural characterization of liquid droplets and their components have

Submitted August 29, 2023, and accepted for publication January 22, 2024.
*Correspondence: gjeschke@ethz.ch
Editor: Scott Showalter.
https://doi.org/10.1016/j.bpj.2024.01.023
© 2024 Biophysical Society.
This is an open access article under the CC BY license (http://creativecommons.org/licenses/by/4.0/).

substantially improved our understanding of biomolecular condensates during the past decade, many questions remain open (15). In particular, it is difficult to determine the ensemble structure of proteins in biomolecular condensates, since the major techniques for structure determination, such as X-ray crystallography and cryoelectron microscopy, are not applicable for the condensed phase. NMR spectroscopy is applicable but does not usually provide a sufficient number of restraints. Here, we apply distance distribution measurements in the nanometer range by the electron paramagnetic resonance (EPR) technique double electron-electron resonance (DEER), which has recently been demonstrated to be applicable to liquid droplets (16).

The multifunctional RNA-binding protein fused in sarcoma (FUS) is a component of stress granules and is involved in numerous cellular processes, such as DNA repair and several RNA processing steps, including transcription, splicing, transport, and translation (17,18). FUS comprises several IDRs, including an N-terminal domain (NTD) rich in glutamine, glycine, serine, and tyrosine (QGSY-rich region) and three arginine-glycine-glycine-rich domains (RGG domains). In addition, it contains two globular domains, an RNA-recognition motif, and a zinc finger. The QGSY-rich region of FUS (residues 1–165) can phase separate as a separate entity (19). This process is driven by a combination of hydrophobic, hydrogen bond, and π-interactions (20). The interactions are not confined to a single protein region and involve all major residue types. This protein region remains disordered upon liquid demixing, and there is no indication for the formation of any secondary structure elements (19,20). The phase-separating properties of the QGSY-rich region are further enhanced in the presence of the RGG1 domain (residues 166–267), stemming probably from cation-π interactions between the aromatic residues in the QGSY-rich domain and the arginine residues in the RGG1 domain (21,22). This NTD of the protein is the main driver of liquid-liquid phase separation of FUS (22) and is the focus of this work. The annotated sequence of FUS NTD, comprising the QGSY-rich region and the RGG1 domain, is shown in Fig. 1 *A*. Fig. 1 *B* shows disorder predictions by fIDPnn (23), which performed consistently well in a recent critical assessment of protein intrinsic disorder prediction (24) and by AlphaFold2 (25), whose pLDDT confidence prediction was found to be a competitive predictor of disorder (26). AlphaFold2 predicts the whole NTD to be disordered, whereas fIDPnn reports part of RGG1, in particular near the C terminus of this domain, as weakly ordered.

Based on distance distribution measurements by EPR in agarose-gel-stabilized biphasic samples of FUS (16) and

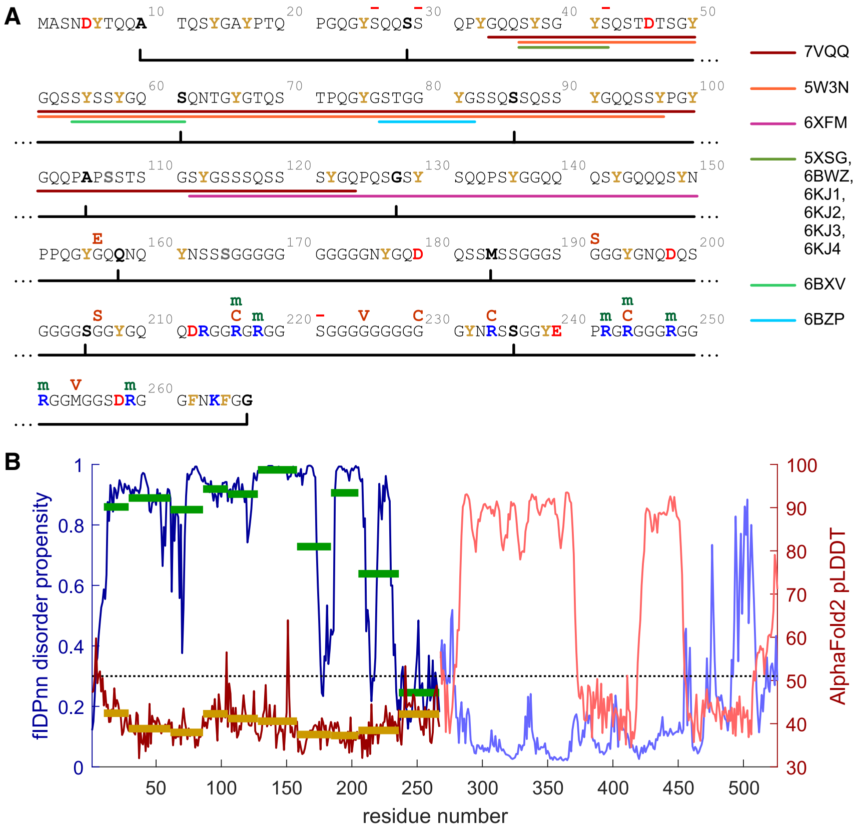

FIGURE 1 (*A*) Annotated sequence of FUS NTD, comprising the QGSY-rich region (residues 1–165) and the RGG1 domain (residues 166–267). Our construct contains three additional N-terminal residues (GSS) remaining from the linker of a GB1-tag used in protein preparation. Residues that are potentially important in multi-valent interactions driving LLPS are typeset in bold and colored. Residues mutated for spin labeling are typeset bold and black (mutations for ensemble structure determination) or gray (mutations for ensemble validation). The black lines with vertical pointers to spin-label sites visualize sections for which distance distributions were measured. Colored lines denote sections for which structures have been deposited in the PDB, with access codes given in the legend on the right. Crimson single-letter codes above the sequence denote established mutations known to cause amyotrophic lateral sclerosis. Red minus signs above the sequence denote phosphorylation sites and green letters “m” denote arginine methylation sites. The information was compiled from UniProt entry P35637. (*B*) Disorder predictions for full-length FUS by fIDPnn (23) (*blue/light blue and left y axis*) and AlphaFold2 (23) (*red/pink and right y axis*). FUS NTD (1–267) is shown in dark colors and the remainder of the protein in light colors. The horizontal dotted line denotes the disorder threshold of fIDPnn. Thick horizontal lines are mean values of the fIDPnn (*green*) and AlphaFold2 prediction (*orange*) for the chain sections for which we measured distance distributions. To see this figure in color, go online.

analysis of the data in terms of Gaussian distributions (27), we here derive ensemble models for FUS NTD dispersed in the protein-poor phase and FUS NTD condensed to a protein-rich phase after LLPS. We compare these ensembles to an ensemble for denatured FUS NTD in a buffer containing 3 M urea and to an unrestrained ensemble generated by an approach (28) that takes into account only residue-specific Ramachandran statistics. The paper is structured as follows. First, we demonstrate that all 10 sections of FUS NTD for which we measured distance distributions are slightly more compact in the condensed state than in the dispersed state, whereas the denatured state is the least compact one. We then show that ensemble models of all three states are approximated well by polymer random coils in a good solvent. By considering the deviation of section root-mean-square (RMS) end-to-end distances from expectations for a random coil (29) we find subtle differences between the ensembles, in particular for the section 128–158, which is more extended in the denatured and dispersed states. We demonstrate that, despite the random-coil character, positive and negative charge density as well as the propensities to act as cationic or aromatic sites in cation-π interactions remain spatially separated. We consider the correlation between the radius of gyration and asphericity (30) in the ensemble model for the condensed state as well as hydration of the QGSY-rich and RGG1 domains. Finally, we discuss our results in the context of previous work on LLPS of the QGSY-rich region of FUS, of polymer physics, and of thermodynamics.

MATERIALS AND METHODS

All reagents were purchased from Merck (Sigma-Aldrich) unless otherwise specified.

Protein expression and purification

Escherichia coli strain BL21 (DE3) was used to express histidine- and GB1-tagged FUS NTD (1–267) inserted into a pET24b vector. Cells were grown at 37°C in M9 media prepared with D_2O for deuterated samples, or in LB-broth medium (BD Difco) for nonisotopically labeled protein. The media were supplemented with kanamycin (PanReac AppliChem) as a selective agent. Once the optical density (OD) at 600 nm reached 0.6, over-expression was induced by addition of 20 μM isopropyl-β-D-1-thiogalactopyranoside (PanReac AppliChem) and subsequently incubated at 20°C for approximately 16 h.

Cell pellets were harvested by centrifugation 10 min at 5000 rpm and 4°C and suspended in lysis buffer (50 mM HEPES, 150 mM NaCl, pH 7.5) followed by sonication. Although the soluble supernatant was discarded, the insoluble pellet was resuspended in resuspension buffer (50 mM HEPES, 0.5 M NaCl, 8 M urea, pH 7.5). The sample was loaded onto a nickel column (Qiagen) and washed first with 15–20 mL of wash buffer A (50 mM HEPES, 0.5 M NaCl, 1 M urea, pH 7.5) and then with 15–20 mL of wash buffer B (50 mM HEPES, 150 mM NaCl, 1 M urea, 20 mM imidazole, pH 7.5). The protein was eluted with 10 mL of elution buffer (50 mM HEPES, 150 mM NaCl, 1 M urea, 250 mM imidazole, pH 7.5). Subsequently, imidazole was removed and the His-tag connected by a Tobacco Etch Virus (TEV) cleavage site was cleaved simultaneously during two dialysis steps (using Spectra/Por dialysis tubing with a molecular weight cutoff of 3 kDa), first against dialysis buffer D1 (50 mM HEPES, 150 mM NaCl, 1 M urea, 5 mM β-mercaptoethanol (PanReac AppliChem)) and then against dialysis buffer D2 (50 mM HEPES, 150 mM NaCl, 6 M urea, pH 7.5). The sample was loaded onto a second nickel column to remove the cleaved tag and any nonspecifically binding molecules. Protein was collected from the flow-through and subsequently concentrated up to 2 mM, unless otherwise specified. Constructs carrying cysteine mutations were concentrated to approximately 100 μM concentration. The purity of the samples was analyzed on a 13% SDS gel. Protein samples were stored at −80°C until further use.

PCR mutagenesis

Point mutations were introduced by a three-step PCR protocol (see Table 1) using primers carrying the desired base change. The PCR reaction contained 5 μL of 10× Pfu buffer (Promega); 4 μL of deoxyribonucleotide triphosphate solution (containing 2.5 mM of each deoxyribonucleotide triphosphate, Invitrogen); 0.5 μL of the forward and backward primer at 100 μM, respectively; 1 μL DNA template at approximately 50 ng/μL; 1 μL of dimethyl sulfoxide (NEB); 1 μL of Pfu polymerase (Promega); and addition of Milli-Q water to a total volume of 50 μL. In some instances, Phusion polymerase (NEB) with 10 μL of 5× Phusion buffer (NEB) was used as an alternative to Pfu polymerase and 10× Pfu buffer.

The methylated, nonmutated parental DNA template was then digested by addition of 1 μL of Dpn I (NEB) to the PCR product and incubation at 37°C for approximately 1–2 h. Subsequently, the Dpn I-treated DNA was amplified in *E. coli* cells (strain TOP10) and isolated using a MiniPrep kit (Qiagen or Macherey-Nagel). For constructs with several mutations, the protocol was applied repeatedly. The incorporation of the correct mutation was verified by Sanger sequencing (Microsynth).

Site-directed spin labeling

The protein solution was diluted to a protein concentration of 30 μM and incubated for approximately 1 h with 5 mM DTT (Dithiothreitol, PanReac AppliChem). The reducing agent was washed out with a desalting column packed with G-25 resin (GE Healthcare). Depending on the sample volume either a PD10, PD MidiTrap, or PD MiniTrap column was used for the desalting steps. The sample was then incubated for approximately 2 h with a fivefold to 10-fold excess of the nitroxide spin label ((1-oxyl-2,2,5,5-tetramethylpyrroline-3-methyl)methanethiosulfonate, Toronto Research Chemicals). Subsequently, free spin label was washed out using a desalting column packed with G-25 resin. The protein was then concentrated in Amicon Ultra-4 Centrifugal Filter Units to a concentration of 900 μM. These steps were performed in dialysis buffer D2 to keep the protein soluble. The labeling efficiency was determined by continuous-wave (CW) EPR spectroscopy.

Iodoacetamido and maleimido spin labels were also tested, but protein precipitated upon incubation with these labels and free spin label was still present in the samples after extensive washing.

TABLE 1 PCR Protocol Employed for FUS Mutagenesis

Step	Temperature	Time
Initial denaturation	95°C	1 min
23 cycles	95°C	20 s
	55–71°C	30 s
	72°C	8 min
Final extension	72°C	5 min
Hold	4°C	–

Sample preparation in agarose gel

General procedure

To form stabilized liquid droplets of FUS, agarose buffer (30 mM HEPES, 200 mM KCl, 0.5% (w/v) agarose (Thermo Fisher), pH 7.3) was boiled to solubilize agarose powder and then cooled at room temperature. Agarose buffer was still liquid at approximately 55°C and was added to an Eppendorf tube containing the protein sample. The mixture was quickly transferred to a preheated sample tube (~55°C). Due to the small volume used, the temperature drops quickly, leading to liquid-droplet formation and agarose gelation. The residual urea concentration was 0.6 M. The same procedure was used for the denatured-state sample, with the exception that, in addition, the agarose gel contains 3 M urea to keep the protein soluble.

Agarose-gel sample preparation for DEER experiments

Spin-labeled protein was diluted 1:10 in dialysis buffer D1 yielding a protein concentration of approximately 90 μM in 50 mM HEPES, 150 mM NaCl, 1.5 M urea, pH = 7.5 buffer. To minimize intermolecular distance contributions, we added wild-type protein for spin dilution purposes. The stock concentration of the wild-type protein was approximately 1.25 M for denatured samples, and 2.5 mM for biphasic samples (both in dialysis buffer D2). Spin-labeled protein and the wild-type protein were premixed in an Eppendorf tube to yield a spin dilution factor of 1:10 for denatured samples and 3:80 for biphasic samples (total volume: 6.3 μL). Boiled agarose buffer (containing 3 M urea for denatured samples) at a temperature of approximately 55°C was added to the protein mixture to yield a final protein concentration of 50 μM for denatured samples and 200 μM for biphasic samples at a total volume of 40 μL. Then 35 μL were then transferred to a preheated sample tube (~55°C, 3 mm OD fused quartz, 0.3 ± 0.1 mm wall thickness, Aachener Quarzglas Technologie Heinrich) and the mixture was allowed to cool down before shock-freezing in pre-cooled isopentane.

Dispersed monophasic samples were prepared with a protein concentration of 5 μM to ensure that the protein does not undergo phase separation. These samples were prepared under the same buffer conditions as biphasic samples. Spin-labeled protein was premixed with dialysis buffer D1 and D2 to a total volume of 6.3 μL (the same volume used for the biphasic sample preparation before the addition of agarose buffer to ensure the same buffer composition). The dialysis buffers D1 and D2 were added at appropriate ratios to achieve a final urea concentration of 0.6 M after addition of all components. Warm agarose buffer (30 mM HEPES, 200 mM KCl, 0.5% (w/v) agarose, pH 7.3) was then added to this mixture to yield 40 μL, and 35 μL of the sample was transferred to a preheated tube. Once the sample temperature dropped to room temperature, the sample was shock-frozen in pre-cooled isopentane.

All samples were prepared with water of natural isotope abundance, except for the denatured-state sample for the four-pulse DEER measurement, which was prepared with D_2O to prolong the phase memory time.

Samples were stored in a liquid-nitrogen container.

Light microscopy

Differential contrast imaging was performed at the ScopeM facility with a Leica DMI6000B microscope and using a 63×1.4 NA oil objective. The 20-μL samples with 50 μM total protein concentration prepared in agarose buffer were loaded onto a glass coverslip (Bellco) and covered with an additional smaller coverslip (Menzel). To minimize evaporation, samples were sealed using nail polish (Electron Microscopy Sciences).

Turbidity measurements

For turbidity measurements, 20-μL liquid-droplet samples (50 μM total protein concentration) in agarose buffer were prepared as described above and pipetted while liquid onto 384-well plates (CORNING 4581). Turbidity at 600 nm was measured using a Synergy 2 (Biotek) microplate reader at room temperature. Agarose buffer was used as blank. All samples were examined in triplicate.

Pulsed EPR measurements

Pulsed EPR measurements were performed on a homebuilt Q-band spectrometer (31) or a Bruker Elexsys E680 spectrometer equipped with an incoherent arbitrary waveform generator (32). A temperature of 50 K was achieved by liquid-helium cooling and was controlled by a He flow cryostat (ER 4118CF, Oxford Instruments) and a temperature control unit (ITC 503, Oxford Instruments). A sample tube loaded with 35 μL of sample was shock-frozen in pre-cooled isopentane and subsequently inserted into a homebuilt Q-band resonator for 3-mm OD sample tubes (31).

An echo-detected field-swept EPR spectrum was acquired using a Hahn-echo sequence. The pulse sequence for the four-pulse DEER experiment was $\pi/2_{obs} - \tau_1 - \pi_{obs} - t_1 - \pi_{pump} - (\tau_1 + \tau_2 - t_1) - \pi_{obs} - \tau_2$. The pump pulse was applied on the spectral maximum and the observer pulses were applied at a frequency offset of 100 MHz. The 4-pulse DEER measurements on the Bruker Elexsys E680 spectrometer were acquired with a pulse delay τ_1 of 200 ns and a dead-time delay of 80 ns. Measurements on the homebuilt Q-band spectrometer were acquired with a pulse delay τ_1 of 400 ns and a dead-time delay of 280 ns. All traces were acquired using 16-ns pulses and eight-step nuclear modulation averaging with an averaging time step of 16 ns.

For the five-pulse version, the experiment was recorded with the sequence $\pi/2_{obs} - (\tau/2 - t_0) - \pi_{pump} - t_0 - \pi_{obs} - t' - \pi_{pump} - (\tau - t' + \delta) - \pi_{obs} - (\tau/2 + \delta)$ with $\delta = 120$ ns to separate the stimulated echo from the refocused echo. The 5-pulse DEER data were recorded with HS{1,6} pump pulses of 150 MHz in width and conventional 32-ns observer pulses at a frequency offset of 70 MHz. Two traces with shifted artifact were recorded alternately. For the first trace, t_0 was set to 300 ns. For the second trace, t_0 and the initial value of the delay t' before the moving pump pulse were increased by a multiple of the time increment, i.e., 192 ns. For details on the nuclear modulation averaging, see the supplementary information in (33).

DEER data analysis

All DEER data were analyzed using a Matlab-based version of DeerLab (release 0.9.2, available at github.com/JeschkeLab/DeerLab-Matlab) (34) by fitting a single Gaussian distribution as validated earlier (27) and below.

We used a multi-pathway kernel $K(t,r)$, which allows fitting of a distance distribution to four-pulse and five-pulse primary DEER data $V(t)$, including any additional contributions that might arise from other pathways. This kernel has the form

$$K(t,r) = [\lambda_0 + \lambda_1 K_0(t,r) + \lambda_2 K_0(t - T_{0,2}, r)]B(t), \quad (1)$$

where K_0 is the elementary dipolar kernel, λ_0 accounts for the contribution of unmodulated dipolar pathways, λ_1 and λ_2 describe the amplitudes of the modulated dipolar pathways, and $T_{0,2}$ is the refocusing time of the additional modulated dipolar pathway. The background function $B(t)$ is a product of stretched exponential functions as specified below. Although K_0 is fixed, λ_0, λ_1, λ_2, $T_{0,2}$, and the parameters that specify $B(t)$ are fit parameters. For all fitted signals, distributions, and parameters, bootstrapped confidence intervals were estimated from 1000 bootstrap samples drawn from a Gaussian distribution based on the noise in the measurements. The primary DEER data and output files of data analysis are found in database Zenodo: zenodo.8214049.

Analysis of the denatured state and the dispersed monophasic state

The DEER data were modeled according to the kernel above with an exponential background function ($d = 3$). Distance distributions were fitted by a single Gaussian distribution.

Analysis of the biphasic state

Due to the presence of the two different species in the biphasic sample, we modeled the measured primary signal as a linear combination of two dipolar signals $V_{\mathrm{disp}}(t)$ corresponding to spins in the dispersed fraction and $V_{\mathrm{cond}}(t)$ to those in the condensed phase weighted by the fractions f_{disp} and $1 - f_{\mathrm{disp}}$. Using the appropriate kernel functions $K_{\mathrm{disp}}(t,r)$ and $K_{\mathrm{cond}}(t, r)$, which differ by their background function, this model can be expressed as

$$V(t) = f_{\mathrm{disp}} \int K_{\mathrm{disp}}(t,r) P_{\mathrm{disp}}(r) \mathrm{d}r + \left(1 - f_{\mathrm{disp}}\right) \int K_{\mathrm{cond}}(t,r) P_{\mathrm{cond}}(r) \mathrm{d}r. \quad (2)$$

The two components of the signal arise from different distance distributions originating from the dispersed $P_{\mathrm{disp}}(r)$ and condensed $P_{\mathrm{cond}}(r)$ fractions, which were modeled as two Gaussian distributions of the form

$$P(r) = \frac{1}{\sigma\sqrt{2\pi}} \mathrm{e}^{-\frac{(r-\langle r\rangle)^2}{2\sigma^2}} \quad (3)$$

where $\langle r\rangle$ is the mean distance and σ the standard deviation.

Due to the difference in spin concentration inside and outside of the liquid droplets, they also exhibit significantly different background functions: a slowly decaying one

$$B_{\mathrm{disp}}(t) = \mathrm{e}^{-k_{\mathrm{disp}}\left(\lambda_1|t| + \lambda_2\left|t - T_{0,2}\right|\right)} \quad (4)$$

for the dispersed fraction with a very constrained decay rate k_{disp} due to the low local spin concentration, and a rapidly decaying function

$$B_{\mathrm{cond}}(t) = \mathrm{e}^{-k_{\mathrm{cond}}\left(\lambda_1|t|^{d_{\mathrm{cond}}/3} + \lambda_2\left|t - T_{0,2}\right|^{d_{\mathrm{cond}}/3}\right)}, \quad (5)$$

for the condensed fraction. For the dispersed phase we employed an exponential background function ($d_{\mathrm{disp}} = 3$) as the basis function for the multi-pathway background, whereas for the condensed phase we employed a stretched exponential function as the basis function.

We performed a global analysis (35) of two five-pulse DEER signals, whose secondary pathway is shifted in time with respect to each other. All model parameters (f_{disp}, λ_0, λ_1, λ_2, d_{cond}, k_{disp}, k_{cond}, $\langle r\rangle_{\mathrm{disp}}$, $\langle r\rangle_{\mathrm{cond}}$, Γ_{disp}, and Γ_{cond}) were fitted globally to both datasets, except for the refocusing time $T_{0,2}$, which was fitted locally to each dataset. We constrained f_{disp} to $\pm 10\%$ of the fraction of proteins in the dispersed phase as determined by Diffusion Order SpectroscopY (DOSY) NMR experiments and restricted the parameter range of $P_{\mathrm{disp}}(r)$ to the upper and lower 95% confidence interval of the mean and full width at half maximum of the dispersed monophasic distance distribution. Global optimization was employed by means of a multi-start algorithm to ensure that the solution is a global solution.

Ensemble generation and analysis

Ensembles for unrestrained, denatured, dispersed, and biphasic FUS NTD were generated with the software package MMMx (commit f1e3459 from 6 December 2021, downloadable at GitHub: https://github.com/gjeschke/MMMx) (29) with dependencies on SCWRL4 (36). The raw ensemble generation, ensemble fit calculations, and ensemble analysis were performed on the ETH Euler cluster. Protein ensembles were analyzed and visualized with MMMx (commit ddff3f8 from 5 May 2023).

Restrained and unrestrained ensembles were generated using the Flex module and the FUS NTD sequence, including the residual N-terminal sequence GGS left over from TEV cleavage. Flex is a Monte-Carlo conformer generator that samples from residue-specific Ramachandran angle statistics and can consider distance distribution restraints already during backbone building by rejecting conformers as soon as their probability to conform to the restraints drops below a threshold value; 5000 conformers were generated for each ensemble in several runs. In the restrained version, distance distributions were specified by mean distances and standard deviations of the Gaussian fits. The restraint files used for the ensemble generation and all ensemble models can be found in database Zenodo: zenodo.8214049. The restrained raw ensembles were contracted in an ensemble-fitting step using the Gaussian restraints. Ensemble fitting maximizes overlap of the experimental distance distributions with distance distributions simulated the ensemble. In simulation of the distance distributions, spin-label conformation distribution is accounted for by a rotamer library approach. Conformers with populations (weights) smaller than 1% of the largest population are discarded in ensemble fitting. Ensemble fitting of the full raw ensembles converged for the denatured and dispersed ensembles with final sizes of 181 and 551 conformers, respectively. Ensemble fitting of the condensed ensemble was performed on five batches of 1000 conformers each. The contracted ensembles were then combined and an additional ensemble-fitting step was performed with the reduced ensemble to yield a final representative ensemble of 2102 conformers.

The unrestrained ensemble and the representative restrained ensembles were analyzed using the EnsembleAnalysis module of MMMx. The additional GGS residues at the N terminus were left out in plots. Conformers within each ensemble were superimposed by aligning the principal axes systems of their inertia tensors. In the new frame, the x axis corresponds to the minimum moment of inertia and the z axis to the maximum moment of inertia. The N terminus has a smaller x and z coordinate than the C terminus. With the three principal values I_x, I_y, and I_z of the inertia tensor, asphericity A of a conformer is computed as (37,38)

$$A = 1 - 3\frac{I_2}{I_1^2}, \quad (6)$$

where $I_1 = I_x + I_y + I_z$ and $I_2 = I_x I_y + I_x I_z + I_y I_z$. For computing mean asphericity $\langle A\rangle$ of the ensemble, I_2 is replaced by $\langle I_2\rangle$ and I_1^2 is replaced by $\langle I_1^2\rangle$.

Ensemble visualization is based on population-weighted electron density maps of the inertia-frame superimposed conformers. Electron density maps of individual conformers were computed by implementation of an earlier approach (39) in MMMx. The approaches for coloring of isosurfaces of the density maps for electrostatic interaction and cation-π interaction are described in the supporting material. Residue weightings for cation-π interaction are based on (40).

Analysis of hydration

Hydration of FUS NTD conformers was assessed using the Accutar Open Access platform as described in (41). We first sorted the conformers in the ensemble describing the condensed state by similarity according to a distance RMS deviation criterion. Then we submitted PDB files for the first 25 and last 25 conformers to the Accutar Bio Server. Coordinates of the oxygen atoms of the generated water molecules were extracted from the output by the auxiliary MMMx function rd_accutar.m. We identified the coordinated residue in the input PDB file as the residue harboring the protein atom with the shortest distance to the water oxygen atom. Data were analyzed in terms of the average number of water molecules, the standard deviation of this number, and the mean number of water molecules coordinated to each residue type using a Matlab script. We found these numbers to be converged after analyzing 50 conformers at the level required for our discussion.

RESULTS

FUS NTD compacts over its whole length upon LLPS

Distance distributions in the nanometer range can provide restraints for ensemble modeling of IDRs of proteins (28).

In recent work, we modeled the conformational ensemble of the glycine-rich domain (133 residues) of the RNA-binding protein heterogeneous nuclear ribonucleoprotein (hnRNP) A1 based on 19 distance distribution restraints and demonstrated by jackknife resampling that the model was sufficiently restrained (27). In later work, we found this model to be in good agreement with a small-angle x-ray scattering (SAXS) curve and in reasonable agreement with paramagnetic relaxation enhancement NMR data (42). The case of FUS in biomolecular condensates is more challenging for three reasons. First, the presence of a zinc finger in full-length FUS interferes with spin labeling. Therefore, we focus on the NTD comprising residues 1–267. However, this deprives us of a folded domain and thus of reference spin-labeling sites that could be used for the relative localization of the IDRs ("beacon" sites). Second, the distance range of DEER measurements depends critically on phase memory time of electron spins, which is therefore prolonged often by using deuterated solvent and sometimes by using deuterated protein (43). With deuterated FUS NTD, we found substantially different phase separation behavior than with natural isotope abundance protein (Fig. S1 in supporting material). Neither is it advisable at our current understanding to deuterate the solvent, as this has been found to affect phase transition temperatures of polymer hydrogels (44) as well as small-angle neutron scattering of protein gels (45). It has been pointed out that substitution of water protons by deuterons modifies the balance between intramolecular and hydration interactions (46). Since Terahertz spectroscopy has revealed that hydration interactions change upon LLPS (47,48), we refrain from solvent deuteration in the current study. We have recently established methodology (16) based on the five-pulse DEER experiment (49), which provides access to distances up to about 60 Å in FUS liquid droplets for natural isotope composition. However, this is still limiting for a construct with 267 residues. Third, paramagnetic relaxation enhancement measurements in the condensate by NMR spectroscopy do not provide spatial information (20) and SAXS does not provide information on the conformational distribution of individual protein molecules in a condensate. In principle, protein deuteration could provide contrast in small-angle neutron scattering, but such a strategy is not advisable, as discussed above. Hence, we are deprived of the advantages (50) of integrating distance distribution restraints with data from other techniques for ensemble structure determination.

In this situation, we opted for mapping the conformational ensemble by a set of distance distributions with mean distances near the middle of the accessible distance range (30–40 Å). This entails sequence separations between labels of about 20–30 residues (28). Restrictions on the choice of labeling sites further complicate the situation. The NTD of FUS contains no native cysteines, requiring the introduction of cysteines in engineered positions for site-directed spin labeling. The accessibility of a residue and the potential disruption of the protein's structure or function need to be considered when introducing mutations with the purpose of site-directed spin labeling. The accessibility of a site in structured protein domains for labeling is typically probed by a rotamer analysis using the protein's structure (51). In our case, residues in IDRs are expected to be accessible. Our main concern here is potential disruption of the phase separation behavior by the labels. Therefore, we excluded sites if the physicochemical properties of the amino acid differed strongly from the ones of cysteine, the candidate involved a mutation site previously identified in patients (52), the mutation involved amino acids or motifs relevant for phase separation (21), or the residues are involved in RNA binding (53). Avoiding sites within the NTD that can form fibrillar assemblies (54–58) (Fig. 1) was not always possible. There is no indication that structured states are formed upon phase separation of FUS NTD (16,20), and, hence, mutations on the fibril cores should not influence LLPS.

With these considerations in mind, we selected the mutation sites A10, S29, S61, S86, A105, G128, Q158, M184, S205, S236, and G267, which cover the entire NTD. These mutation sites divide the NTD into 10 sections that are flanked by two engineered cysteines, allowing us to characterize the domain by intramolecular distance measurements by EPR. Six sections cover the QGSY-rich region, three the RGG1 domain, and one section is localized in the boundary between both domains. Three additional double cysteine mutants were generated for ensemble validation: S89C S107C, S165C M184C, and M184C S236C. We spin labeled all constructs with a methanethiosulfonate spin label ((1-oxyl-2,2,5,5-tetramethylpyrroline-3-methyl)methanethiosulfonate), which is the size of a large native amino acid side chain with a hydrophobicity in between that of methionine and tryptophan, suggesting a comparatively small perturbation on the system (59). Importantly, spin-labeled cysteine double mutants retain the ability to phase separate (Fig. S2 *A*) with similar turbidity values to the wild-type protein (Fig. S2 *B*).

In previous work, we established by ensemble simulations that distance distributions for spin-labeled peptide random coils are well approximated by Gaussians (27). In experiments for two of the double mutants of FUS, we found that Gaussian distributions fitted experimental DEER data well for the dispersed monophasic state, the biphasic state, and bulk condensed FUS NTD (16). We checked that distance distributions back-calculated from an unrestrained ensemble of FUS NTD are also very well approximated by Gaussians (Fig. S3). By analyzing DEER data for biphasic FUS NTD samples in terms of Gaussian distributions for the dispersed fraction (red) and condensed fraction (blue), we find that all 10 sections are more compact in the biomolecular condensate (Fig. 2). This confirms and extends our earlier finding (16) of compaction of FUS NTD upon LLPS. Note that starting values for the fit parameters for

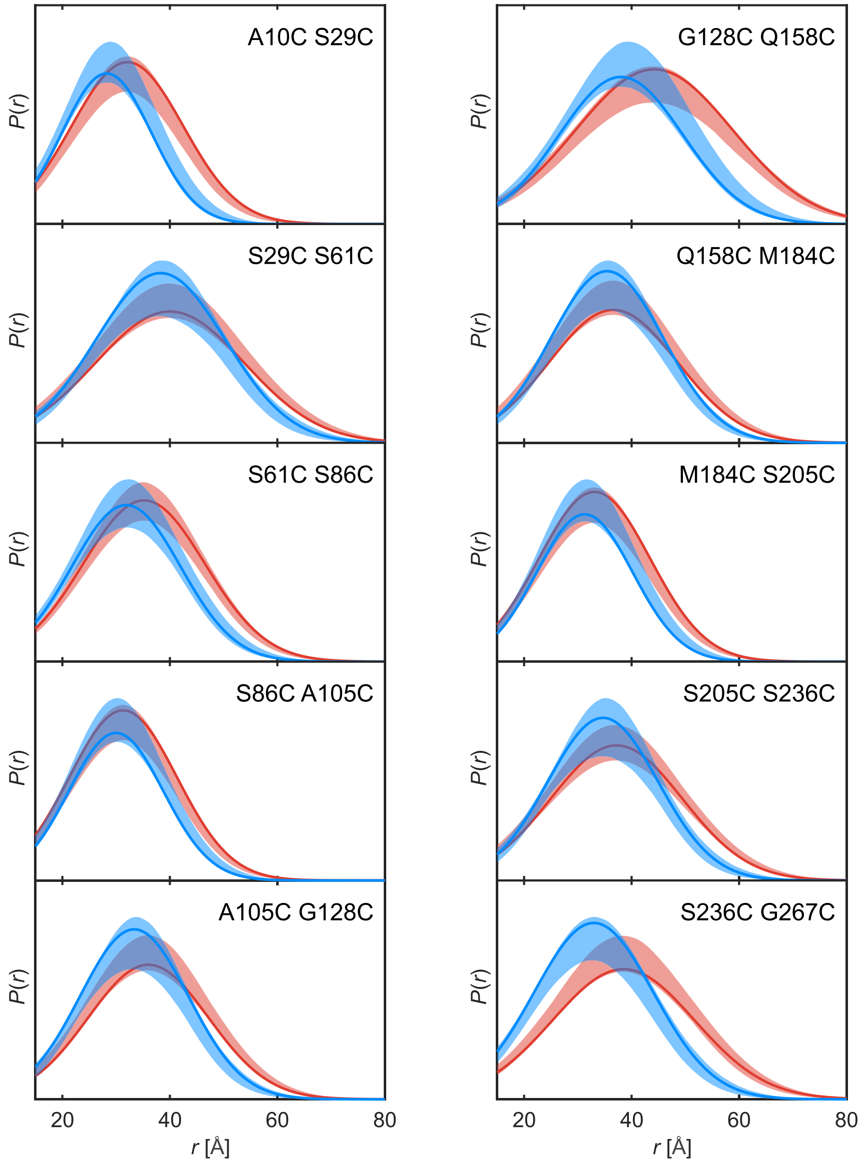

FIGURE 2 Distance distribution restraints of the NTD in the biphasic state. Spin-label positions are indicated in the upper right corner of each panel. The dispersed fraction is shown in red, the condensed fraction in blue, and the 95% confidence intervals obtained via 1000 bootstrap samples as shaded areas. The primary DEER data are visualized in Fig. S7 and the fit residuals in Fig. S8. To see this figure in color, go online.

the dispersed state (Table S1) were derived from measurements on monophasic dispersed samples (Fig. S4). The primary data for the monophasic dispersed (Fig. S4) and biphasic (Fig. S5) samples are fitted very well by the Gaussian distributions, as is apparent also from the fit residuals for the biphasic sample (Fig. S6). For comparison, we measured DEER data for all double mutants for FUS NTD in its denatured state (3 M urea), which are also well fitted by Gaussian distributions (Fig. S7 for primary data and Fig. S8 for corresponding distance distributions). Parameters of the Gaussian fits for the denatured state and for the dispersed and condensed fraction in the biphasic state are shown in Table S2. Not unexpectedly, the mean distance is longer for all sections in the denatured state than in the dispersed state. Hence, FUS NTD extends upon adding 3 M urea, whereas it compacts upon undergoing LLPS. We cannot safely exclude different urea concentrations in the dispersed and condensed phase as a reason for the compaction, but we will see below that compaction is in line with expectations from polymer physics without invoking an effect due to urea.

Dispersed and condensed states behave as random coils under good-solvent conditions

Based on the restraints in Table S2, we modeled three ensembles of FUS NTD that we call the denatured, dispersed, and condensed ensemble in the following. They were deposited in the Protein Ensemble Database with accession numbers PED00495, PED00494, and PED00493, respectively. Given the very similar restraints for the dispersed state obtained in a dilute monophasic sample and the

biphasic sample, we computed only one dispersed-state ensemble based on the restraints from the biphasic sample. In a first step, we generated raw ensembles using residue-specific Ramachandran angle distributions for sampling and the Gaussian restraints for rejecting conformers that were in disagreement with the experimental data (28). In a second step, we reweighted the ensembles by fitting populations (29,50). This step provides smaller ensembles, as we reject conformers with populations that are by more than a factor of 100 smaller than the largest conformer population. Distance distributions back-calculated from the such obtained ensembles are in virtually perfect agreement with the Gaussian distributions (Figs. S8–S10), as quantified by their overlap

$$o = \sum_i \min\{P_{sim}(r_i), P_{exp}(r_i)\}, \tag{7}$$

where $\mathbf{P}_{sim}(\mathbf{r})$ and $\mathbf{P}_{exp}(\mathbf{r})$ are the simulated and experimental distance distributions, given as normalized vectors. The overlap can range from 0 for disjoint to 1 for identical distributions. We find values between 0.979 and 0.996, much closer to unity than for the weakly structured glycine-rich IDR of hnRNP A1 (27,42). For the denatured ensemble with only 181 conformers, the overlap deficiency $1 - o$ of up to 0.022 is most likely dominated by sampling statistics. For the dispersed and condensed ensemble, overlap deficiency is within the combined uncertainties from the experiment and from assuming a Gaussian distribution.

For ensemble validation, we measured data for the three additional double mutants (S89C S107C, S165C M184C, and M184C S236C) in the denatured and biphasic states (Fig. 3). Overlap deficiency of the corresponding ensembles is larger than for the distributions used in ensemble fitting and ranges up to 0.18 (M184C S236C condensed state). The deviations are highly significant only for S89C S107C and S165C M184C in the denatured state and for M184C S236C in the condensed state. For the broader distribution M184C S236C, overlap deficiency is dominated by sampling statistics for the denatured ensemble (181 conformers) and dispersed ensemble (561 conformers). Back-calculated distributions S89C S107C and S165C M184C are rather close to the uncertainty bands for the dispersed and condensed ensembles. The ensemble model of the denatured state underestimates the extension with respect to the unrestrained ensemble for S89C S107C and S165C M184C. This indicates that adding more experimental data could still improve the model. The situation is less clear-cut for M184C S236C in the condensed fraction of the biphasic sample. We cannot safely exclude that the experimental uncertainty estimate is too optimistic, as the primary DEER data is rather short for detecting distances up to 80 Å (43). For this reason, we did not attempt to measure further double mutants with a sequence separation of more than 50 between spin labels.

We compared the restraint sets for the three ensemble models among themselves and to the unrestrained ensemble by plotting the mean values $\langle r \rangle$ and standard deviations σ of the 10 measured sections versus sequence separation ΔN (Fig. 4). Within this range of sequence separations, the dependence of both parameters on ΔN is nearly linear. We thus added linear fits as a guide to the eye. The only section with a highly significant deviation from this linear dependence is G128C Q158C in the denatured and dispersed cases. This section is marked in Fig. 4 by a purple vertical line. A comparison reveals that only the denatured state deviates substantially from the unrestrained ensemble. In the dispersed state, FUS NTD is slightly more extended than in the unrestrained ensemble, with widths σ of the distance distributions being very similar to the unrestrained case except for section G128C Q158C. The condensed state exhibits mean distances whose difference to the ones in the unrestrained ensemble is hardly significant. The widths σ, although they have rather large error bars, are systematically lower than for the unrestrained ensemble. Altogether, the

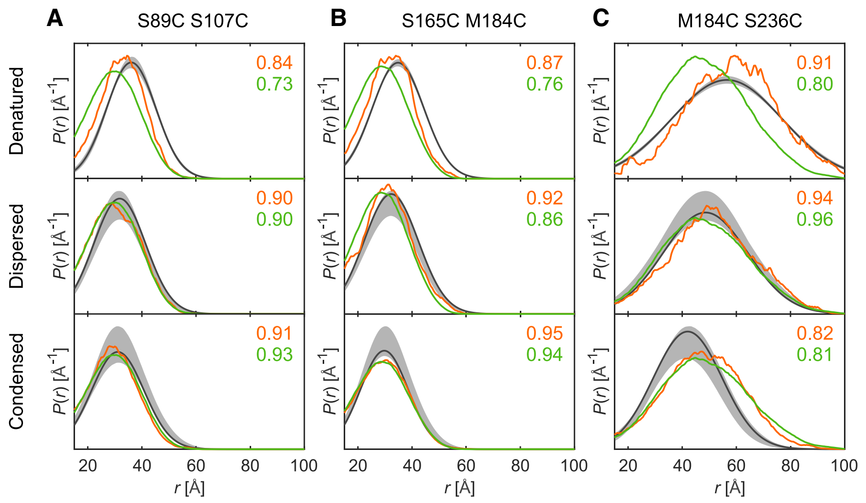

FIGURE 3 Ensemble validation by comparison of experimental and predicted distance distributions. Experimental distance distributions (*gray*) of (*A*) S89C S107C, (*B*) S165C M184C, and (*C*) M184C S236C in the denatured (*top row*), dispersed (*middle row*), and condensed (*bottom row*) states compared to predicted distance distributions of the corresponding ensemble (*orange*) and the unrestrained ensemble (*green*). Overlap values between experimental and predicted distributions are color coded and displayed in the upper right corner of each plot. Shaded gray areas denote 95% confidence intervals obtained via 1000 bootstrap samples. To see this figure in color, go online.

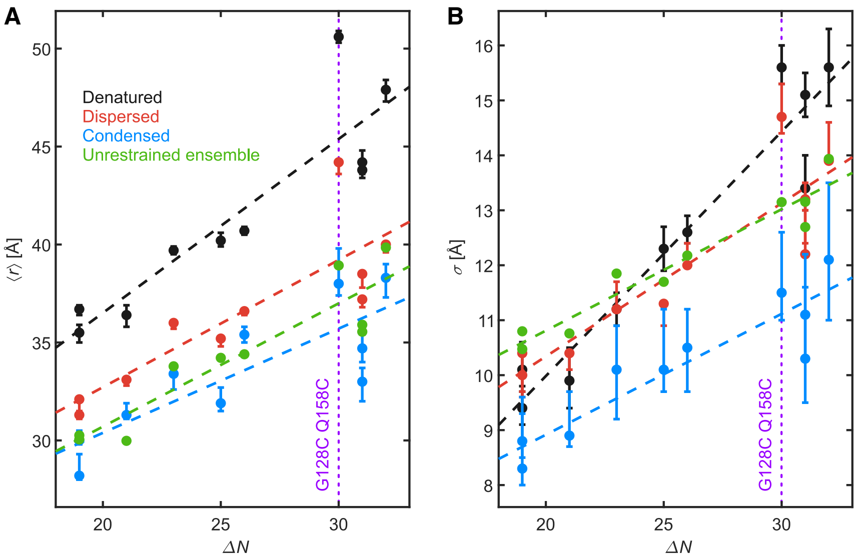

FIGURE 4 Overview of the Gaussian fit parameters of all ensembles. (*A*) Mean distances $\langle r \rangle$ and (*B*) standard deviations σ of experimental Gaussian distance distributions for site pairs in FUS NTD in the denatured (*black*), biphasic dispersed (*red*), and biphasic condensed state (*blue*) compared to the back-calculated values of the unrestrained ensemble (*green*) as a function of the sequence separation ΔN between the labeled residues. Error bars correspond to the 95% confidence intervals. Linear fits (*dashed lines*) are a guide to the eye. The conspicuous section G128C Q158C is marked by the purple vertical line. Note that more than one section can have the same sequence length ΔN. To see this figure in color, go online.

comparison indicates that all ensembles are rather close to random coils, except for section G128C Q158C in the denatured and dispersed state. Unrestrained protein ensembles are known to fit well to a random-coil model (60). Note that the section G128 Q158 harbors the only known missense mutation within the QGSY-rich domain that causes amyotrophic lateral sclerosis (G156E) (61). This mutation was also shown to increase the propensity of aggregation of FUS in vitro and in cell culture (62). In addition, this extended section overlaps with the fibril structure found for an FUS construct composed of the C-terminal half of the low-complexity domain (residues 111–214) (57).

Parts of FUS NTD slightly contract or expand compared to a random coil

To obtain further insight into this finding, we rely on the fact that the RMS end-to-end distance $R = \sqrt{\langle R^2_{\mathrm{end-end}} \rangle}$ of each section of a random coil exhibits the same scaling behavior with section sequence length ΔN. There exist $N+1-\Delta N$ sections with sequence separation ΔN, whereas in FUS NTD ΔN is running from 1 to $N = 267$. Hence, we have R values for 35,778 distinct chain sections. For a random coil, we expect that R for all these sections conforms to the same scaling law with respect to ΔN,

$$R = b \cdot \Delta N^{\nu} \qquad (8)$$

where $\nu = 0.5$ corresponds to the Gaussian coil encountered in a θ solvent, $\nu = 1/3$ to the poor-solvent limit, and $\nu = 3/5$ to the good-solvent limit in Flory theory of real polymer chains. For the good-solvent limit, more elaborate field theory predicts a value of $\nu = 0.588 \pm 0.001$ (63). As there exist $N+1-\Delta N$ sections with sequence separation ΔN, an ensemble model provides distributions $R(\Delta N)$. We have demonstrated before that such distributions can reveal deviations from random-coil behavior (27). The average $\overline{R}(\Delta N)$ over all sections with the same ΔN is also expected to scale with ΔN according to Eq. 8. Deviation from random-coil behavior can be recognized in this dependence (27).

For all FUS NTD ensembles, we find narrow distributions of R at given sequence separation ΔN and rather good agreement of the scaling of $\overline{R}$ with ΔN with random-coil behavior (Fig. 5). For small ΔN, the chains are slightly more extended than expected and for large ΔN they are slightly less extended. We tentatively assign this behavior to the RGG1 domain being slightly more compact (see below) and the QGSY-rich domain contributing more shorter sections due to being longer. Parameters b and ν of the random-coil models for all ensembles are reported in Table 2 together with ensemble size, radius of gyration R_g, and the geometric mean $\overline{o} = \left(\prod_{m=1}^{M} o_m\right)^{1/M}$ of the overlaps of all experimental distance distributions for a given ensemble with the distance distributions back-calculated for the same label pairs from the unrestrained ensemble. The empirical repeat unit length $b = 5.1$ Å is the same for all ensembles. It is longer than $R_0 = 1.98 \pm 0.37$ Å found by a simpler model of rigid sections with flexible linkers (60). Our value is similar to $b = 5.5$ Å found in the context of interpreting Förster resonance energy transfer (FRET) experiments on intrinsically disordered proteins (64). The scaling exponent of 0.583 for the unrestrained ensemble is very close to the theoretical value of 0.588 for a random coil in a good solvent. The same applies to the dispersed and condensed ensemble, whereas the denatured ensemble features, on average, more extended chains than expected for a random

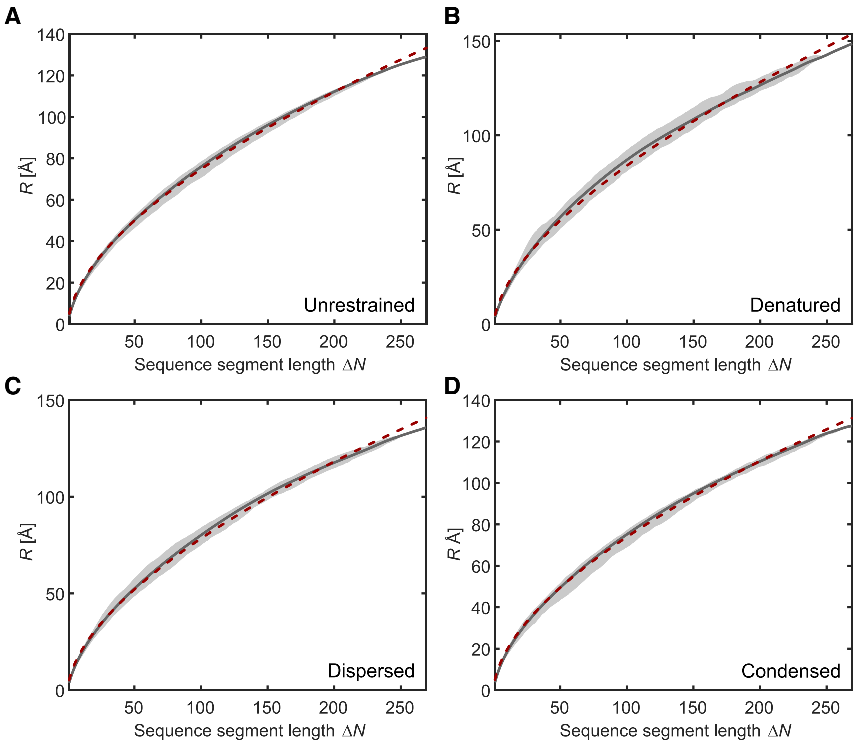

FIGURE 5 Distribution of RMS Cα-to-Cα distances R (*gray shaded areas*) for all sections with sequence separation ΔN, their averages $\overline{R}(\Delta N)$ (*solid gray lines*), and random-coil fits according to Eq. 8 (*crimson lines*) for (*A*) the unrestrained ensemble, (*B*) the denatured ensemble, (*C*) the dispersed ensemble, and (*D*) the condensed ensemble. To see this figure in color, go online.

coil. Values of ν slightly larger than 0.6 have also been found for denatured proteins in a Förster resonance energy transfer study, albeit at larger concentrations of urea (64). Our finding of good-solvent conditions for FUS NTD in the dispersed state is in contrast to earlier observations for proteins with Q/N/S-rich sequences that undergo LLPS (9), such as polyglutamine (65) and the yeast prion protein Sup35 (66) as well as for polar oligomers $(GG)_15$ and $(GS)_8$ (67), which collapse in aqueous solution in their dispersed state. Altogether, the random-coil model appears to be a good approximation for all four ensembles of FUS NTD. However, the small deviations from this model may be significant.

The C-terminal section of the QGSY-rich domain appears to be extended

To obtain more insight into the small deviations from random-coil behavior, we subtract from the section RMS end-to-end distance R the average $\overline{R}$ over all sections with the same sequence length ΔN. We compute the differences $\Delta R_{i,j} = R_{i,j} - \overline{R}(\Delta N)$ for all chain sections from a residue i ($i = 1 \ldots 267$) to a residue j($j = 1 \ldots 267$). Fig. 6 presents all these differences in a two-dimensional plot. Each point corresponds to one pair of residues. Spin-label site pairs used for restraining the ensemble are denoted by green points above the diagonal, whereas validation pairs are denoted by orange points. Red shades denote sections that are more extended than the average, whereas blue shades denote sections that are more compact. In Fig. 5, the ΔR correspond to the vertical difference of a point in the gray area from the solid gray line.

For the unrestrained ensemble (Fig. 6 *A*), we find that the QGSY-rich domain (residues 1–165) is more extended and the RGG1 domain (residues 166–267) more compact than average. Generation of the unrestrained ensemble (28) does not consider any intramolecular interactions beyond clash avoidance and those interactions that contribute to residue-specific Ramachandran plots. Hence, this effect can only be explained by bulk of sidechains and Ramachandran preferences. Indeed, sidechains are, on average, less bulky in the RGG1 domain than in the QGSY-rich domain (Fig. S11 *A*) and the population of extended conformations in residue-specific Ramachandran plots is larger in the QGSY-rich domain (Fig. S11 *B*). Both features are caused mainly by the larger fraction of Gly residues in RGG1.

The experimentally informed ensembles exhibit additional patterns, most strongly for the dispersed state (Fig. 6 *C*),

TABLE 2 FUS NTD Ensemble Characteristics

	Size	b (Å)	ν	R_g (Å)	$\overline{o}_{\mathrm{ref}}$
Unrestrained	5000	5.1	0.583	51.3	–
Denatured	181	5.1	0.610	58.2	0.77
Dispersed	551	5.1	0.592	54.0	0.93
Condensed	2102	5.1	0.581	50.7	0.91

Values for the ensemble size, chain dimension, radius of gyration, and mean overlap with the back-calculated distance distributions of the unrestrained ensemble $\overline{o}_{\mathrm{ref}}$.

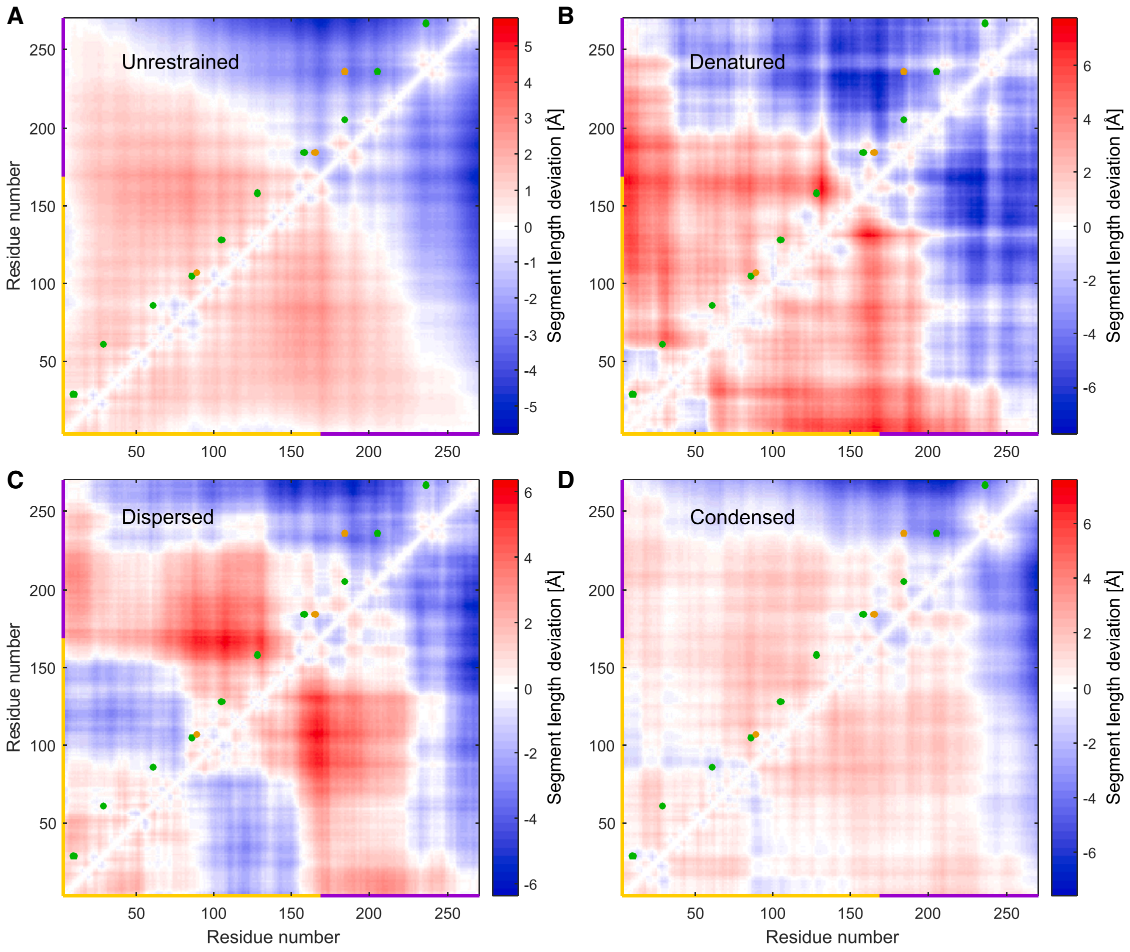

FIGURE 6 Section-length deviation ΔR from the average RMS Cα-Cα distance for a given sequence separation ΔN. The section-length deviations are shown for (*A*) the unrestrained, (*B*) the denatured, (*C*) the dispersed, and (*D*) the condensed ensemble. Blue regions correspond to more compact and red regions to more extended sections, with the corresponding color bars shown on the right. Green points above the diagonal denote pairs of labeling sites used in the generation of the experimental ensembles, whereas orange points denote site pairs used for ensemble validation. The QGSY-rich domain is denoted yellow on the axis, whereas the RGG1 domain is indicated in purple. To see this figure in color, go online.

followed by the denatured state (Fig. 6 *B*) and the condensed state (Fig. 6 *D*). As this feature may depend on measurements of a single double mutant, we performed a technical repeat (see Fig. S12; Table S3 in the supporting material) and repeated ensemble fitting for the dispersed state by using the unrestrained raw ensemble and 1) the original distance distribution restraint, 2) the restraint obtained with the technical repeat, and 3) not restrained on section 128–158. The results are compared in Fig. S13 to the result shown in Fig. 6 *C*. We find that the pattern of extension and compaction is the same in all ensembles, although the extent of extension of section 128–158 is substantially reduced when the corresponding restraint is skipped.

The dependence of the slight extension of section 128–158 on data obtained with just one spin-labeled double mutant raises the concern of a spin-labeling artifact. Molecular dynamics simulations on amyloid-β42 suggested a bias to the conformation ensemble due to insertion of a spin label at the N terminus (68). Kinetics of fibrilization of tau peptides was found to be affected by spin labels at some sites (69). Given that the mean distance between sites 128 and 158 is much larger than the combined size of the two labels and that the two other sections containing one of the two labeling sites, 105–128 and 158–184, have inconspicuous chain extensions, we consider it rather unlikely that the effect is due to the spin labeling. Further work is needed to verify special behavior of section 128–158 and understand its cause.

Properties of the QGSY-rich and RGG1 domains differ despite the random-coil behavior

The differences in the ΔR matrix in the biphasic state between the dispersed fraction (Fig. 6 *C*) and the condensed fraction (Fig. 6 *D*) indicate that some intramolecular interactions in FUS NTD are replaced by intermolecular interactions during LLPS. To shed light on this issue, we attempted visualization of interaction potentials at ensemble

level (Fig. 7). Note that such visualization is necessarily artificial to some extent, because individual conformers interact with each other rather than superimposed ensembles. Indeed, it is not obvious how the conformers should be superimposed. We aimed for a compact spatial representation that keeps N termini and C termini of individual conformers close to each other. To that end we transformed each conformer into the principal axes system of its inertia tensor, selecting the x and z axes as the axes with the smallest and largest moment of inertia, respectively. We chose the center of mass as frame origin. Directions of the x and z axes were selected such that the N terminus had negative x and z coordinates. For each conformer, we computed an electron density map (39) and constructed an ensemble map by population-weighted summation. Isosurface plots of such maps with the y axis upright are shown in Fig. 7.

This representation reveals that, on average, conformers in the ensemble are negatively charged in their N-terminal moiety and positively charged in their C-terminal moiety, with positive charge density being larger. Given the sequence of the NTD (Fig. 1), this finding is not surprising. The 10 positively charged residues are all situated in the RGG1 domain, which features only five negatively charged residues. The QGSY-rich domain contains only two negatively charged residues. Likewise, the cation-π interaction potential has its π-interaction propensity mostly in the N-terminal moiety (the QGSY-rich domain) and its cationic propensity in the C-terminal moiety (RGG1 domain). The visualization suggests that π-cation interaction may be more important than electrostatic interaction for the behavior of FUS NTD.

The spatial representation in Fig. 7 further indicates that, on average, conformers do not feature spherical symmetry. Such shape anisotropy of random-coil polymers is a well-known phenomenon (70). It can be quantified by the asphericity of the tensor of inertia (37) and has recently been discussed in the context of LLPS of the RGG domain of LAF-1 (30). Ensemble asphericity $\langle A \rangle$ of the random-flight chain (70) computes to 0.128. Since more extended chains have larger anisotropy, asphericity A of individual conformers is correlated to their radius of gyration R_g and to their hydrodynamic radius. In Fig. 8, we explore this correlation for the unrestrained and condensed ensemble of FUS NTD. Ensemble asphericities are 0.137 for the unrestrained and 0.135 for the condensed ensemble, close to the expectations for a random coil. The correlation between asphericity and radius of gyration for the condensed ensemble of FUS NTD (Fig. 8 *B*) is surprisingly similar to the one of LAF-1 RGG displayed in the supporting information of (30). We note, however, that in our case the probability density maxima should not be assigned to separate subensembles. Most likely, they arise from statistical noise due to the relatively small number of 2102 conformers in the ensemble, with some having a much lower population than others. For the unrestrained ensemble with 5000 conformers with uniform populations, we observe a rather smooth distribution (Fig. 8 *A*). Altogether, analysis of asphericity supports the view that condensed FUS NTD behaves as a random coil.

Finally, we assessed the hydration (71) of FUS NTD conformers from the condensed ensemble using prediction of hydration sites by a neural network algorithm (41). The algorithm predicts that, on average, 773 water molecules hydrate an isolated FUS NTD molecule. Conformation-dependent fluctuations give rise to a standard deviation of 44 coordinated water molecules. The mean number of coordinated water molecules per residue is 3.0 for the QGSY-rich domain and 2.6 for the RGG1 domain. For comparison, for the dispersed ensemble of the RNA-binding protein hnRNP A1 derived from DEER and SAXS restraints (42), the same algorithm predicts per-residue hydration of 1.5 for the folded domain and 2.8 for the glycine-rich domain. Although the latter domain is more compact than FUS NTD, the predicted extent of hydration is similar. In FUS NTD, the most strongly hydrated residue types are, in this order, Ser, Gly, Gln, and Tyr (Fig. 9). Except for Gly, which contributes strongly to hydration by its abundance, all these residue types have been implicated with intermolecular interactions in the condensed state (19,20,72)

DISCUSSION

We characterized the conformer distribution of FUS NTD (1–267) in its denatured, dispersed, and condensed state

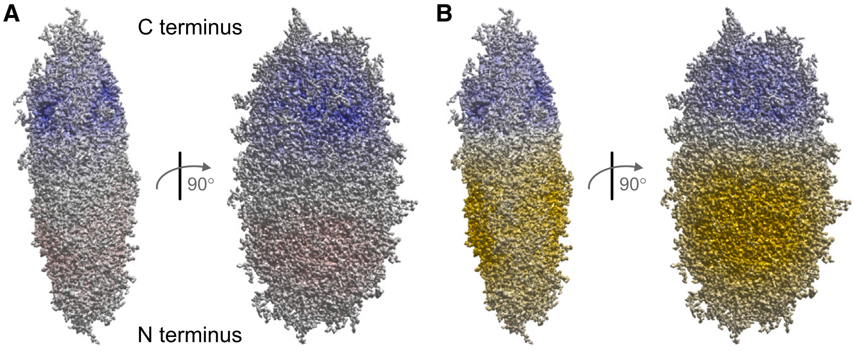

FIGURE 7 Visualization of (*A*) electrostatic and (*B*) cation-π interaction potentials of FUS NTD in the condensed state. The spatial extension of the ensemble is visualized by transforming all conformers to the principal axes frame of their inertia tensor (*x axis upright*) and computing a population-weighted pseudo-electron density of the ensemble. Electrostatic potential is visualized from blue (positive) to red (negative) by adding contributions of individual charged residues screened only by the ionic strength of the buffer (150 mM NaCl). Cation-π potential is visualized by adding contributions of individual cations (*blue*) and aromatic residues (*gold*) and assuming decay by the inverse square of the distance. For clarity, full color saturation is assigned to the maximum surface potential encountered. To see this figure in color, go online.

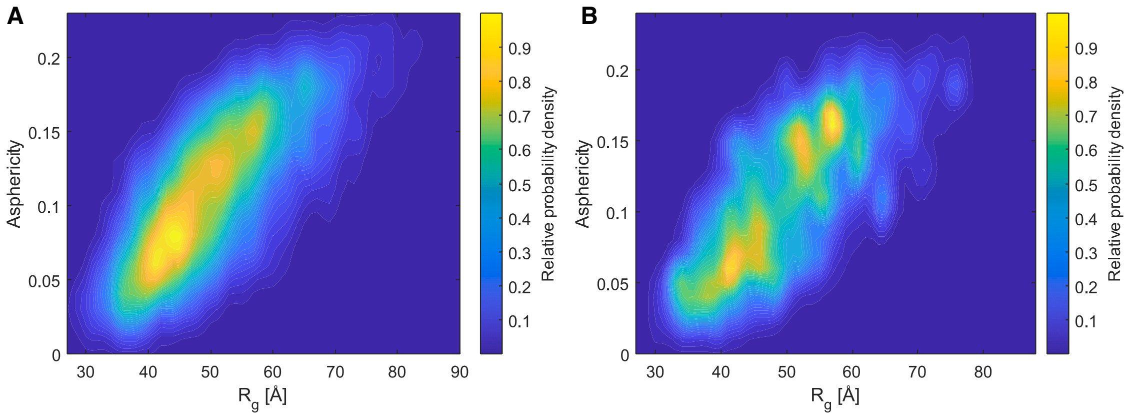

FIGURE 8 Correlation between asphericity A and radius of gyration R_g of FUS NTD conformers in (*A*) the unrestrained ensemble and (*B*) the condensed ensemble. Data are represented as smoothed probability density normalized to maximum probability density. To see this figure in color, go online.

by measuring distance distributions on length scales between about 20 and 80 Å. All experimental datasets were consistent with Gaussian distributions of the label-to-label distance with mean distances ranging from about 31 to 51 Å and standard deviations ranging from about 9 to 16 Å. Taking these distributions as restraints, we derived ensemble models for the three states. All three ensembles are close to random coils in a good solvent. The radius of gyration R_g decreases from the denatured state at 3 M urea concentration (58.2 Å) via the dispersed state in a biphasic sample after LLPS (54.0 Å) to the condensed state in the biphasic sample (50.7 Å). The compaction of FUS NTD between the dispersed and condensed state is observed independently for each of the 10 sections (Fig. 2). Indeed, such a decrease in the coil size with increasing concentration is expected for a polymer in a good solvent (73,74), whereas it is at variance with an earlier prediction from coarse-grained molecular dynamics simulations that predicted expansion of the QGSY-rich domain of FUS upon LLPS (72). The asphericity of 0.135 of FUS NTD in the condensed state is close to the value of 0.128 expected for a random coil. Actually, the approximation of the condensed state by a random coil in a good solvent even improves when considering the two domains of FUS NTD individually (Fig. 10). For the QGSY-rich domain, we obtain $b = 5.13$ Å, $\nu = 0.584$ and for the RGG1 domain we obtain $b = 5.05$ Å, $\nu = 0.568$. Hence, FUS NTD behaves, to a good approximation, as a random-coil block copolymer. The software dScope (75) predicts a slightly higher propensity to LLPS (dScope score) for the QGSY-rich domain (0.873) than for the RGG1 domain (0.756). Notice that the QGSY-rich domain behaves even closer to a random coil in a good solvent than the RGG1 domain.

The condensed state matches an unrestrained ensemble ($R_g = 51.3$ Å) within experimental precision. This unrestrained ensemble takes into account only residue-specific Ramachandran angle statistics for random coils derived from PDB structures of proteins (76). Specific intramolecular interactions between residues are expected to be averaged in such statistics. Hence, our result suggests that specific intramolecular interactions are negligible for FUS NTD in the condensed state.

Our results further suggest the absence of any significant secondary structure in either of the three states. This is in line with previous conclusions from NMR and coherent anti-Stokes Raman spectroscopy on a QGSY-rich domain construct consisting of residues 1–163 (19,20). For this construct, the overlap concentration was estimated as 285 mg/mL, whereas the macroscopic condensed phase with $\approx$65% water by volume had a concentration of

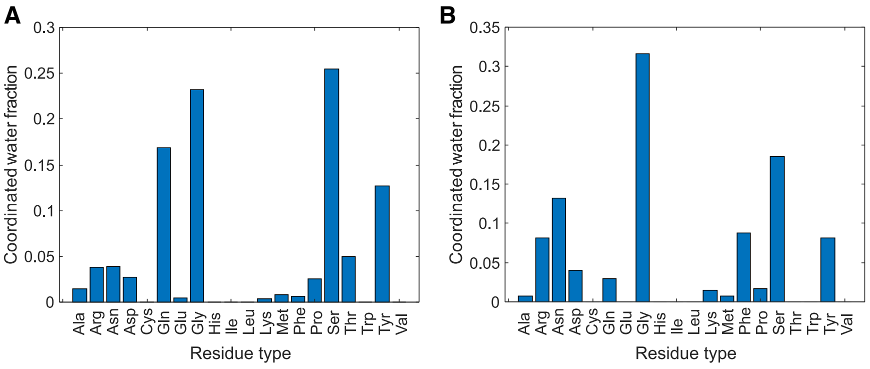

FIGURE 9 Predicted distribution of hydration water molecules over residue types in (*A*) the condensed-state ensemble of FUS NTD and (*B*) the dispersed-state ensemble of the glycine-rich domain of hnRNP A1. In each case, a sample of 50 conformers was analyzed. Hydration sites were predicted by Accutar (41). The hnRNP A1 ensemble was taken from (42). To see this figure in color, go online.

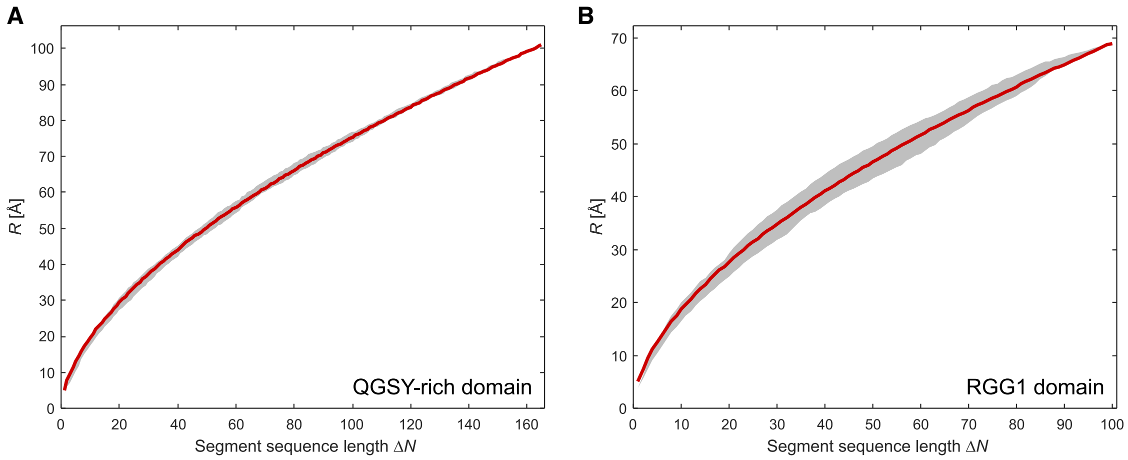

FIGURE 10 RMS Cα-to-Cα distances. RMS Cα-to-Cα distances R for all sections with sequence separation ΔN (*gray shaded areas*), their averages $\overline{R}(\Delta N)$ (*solid gray lines*), and random-coil fits according to Eq. 8 (*crimson lines*) in the condensed state for (*A*) the QGSY-rich domain and (*B*) the RGG1 domain. To see this figure in color, go online.

477 mg/mL. If we accept bounds for the overlap concentration C^* (73)

$$\frac{M}{N_A R_g^3} \geq C^* \geq \frac{3M}{4\pi N_A R_g^3} \tag{9}$$

with $M = 26.3$ kDa, the Avogadro constant N_A and $R_g = 51$ Å, chain overlap should occur in our case at concentrations above 78 to 330 mg/mL. From the preparation of bulk phase samples of our construct in earlier work (16), we can estimate the concentration in the condensate as 20 mM, corresponding to 526 mg/mL. This compares to a concentration of 15 mM for FUS 1–214 found by Raman imaging of single droplets (77). Hence, our data suggest that FUS NTD molecules overlap in the condensate, which is again consistent with the behavior of the QGSY-rich domain in isolation as established in (20).

Although the description of FUS NTD as a random-coil polymer in a good solvent is a good approximation, deviations from this approximation are significant, as seen in Figs. 2, 5, 6, and 10 *B*). Most notably, the RGG1 domain is slightly more compact than the QGSY-rich domain. This effect is observed already in the unrestrained ensemble and can be attributed to the larger glycine content of RGG1. Further differences to random-coil behavior are apparent only in the experimentally restrained ensembles. In particular, section 128–158 is more extended than the other nine measured sections in the denatured and dispersed state. This effect is diminished in the condensed state, where it cannot be safely discerned.

The rather minor change of the conformational ensemble between the dispersed and condensed state and the close approximation of the condensed state by the model of a random-coil block copolymer raise the question of the driving force for LLPS. Regarding the slight compaction of FUS NTD upon LLPS, we note that it is semi-quantitatively predicted within de Gennes' tricritical theory (73,78,79). This theory is based on virial expansion of monomer interaction, taking into account only the first two virial coefficients. The first coefficient W_1 is related to excluded volume interaction and the second coefficient W_2 can be increased by adding sidegroups to the chain and decreased by making the chain more rigid, as remarked upon by de Gennes (78). Higher glycine content thus has two opposing effects on W_2. No specific sidegroup-sidegroup or sidegroup-backbone interactions need to be invoked to explain the reduction of chain size upon an increase of protein concentration. This phenomenon is generic polymer behavior rather than sequence dependent. We would expect it for any IDR whose residue composition leads to good-solvent conditions. We note that the QGSY-rich domain (19,80) as well as FUS NTD (16) feature a decreasing tendency to LLPS with increasing temperature. This implies that the enthalpic contribution to the driving force for LLPS dominates over the entropic contribution (81).

In the following, we assume phase equilibrium between the dispersed and condensed states. For the QGSY-rich domain, half of the protein molecules have been estimated to exchange between the phases within 1.4 s (20). Protein diffusion inside the droplet is much faster than that (16). Given that in our in vitro experiments liquid droplets are stable for hours, the equilibrium assumption is thus expected to be a good approximation. To separate the contribution of hydrated protein to the free energy change upon LLPS from the one of uncoordinated water, we have analyzed the equilibrium by chemical potentials (see supporting material). For the molar contribution to the free-energy change by the hydrated protein, we find $\Delta\mu_{\mathrm{FUS,LLPS}} = -6.8RT$, where RT is the thermal energy. We estimate the contribution of chain entropy change due to compaction as $0.85RT$, almost an order of magnitude smaller and with

opposite sign. Hence, the main contribution to $\Delta\mu_{\mathrm{FUS,LLPS}}$ comes from changes in intramolecular and intermolecular interactions, as one also expects from the sensitivity of LLPS to mutations.

For the change of molar chemical potential of noncoordinated water upon LLPS of wild-type FUS NTD, we find an upper bound $\Delta\mu_{\mathrm{H_2O,LLPS}} < 1.7 \cdot 10^{-3} RT$ (see Eq. S7). Given that breaking a single hydrogen bond corresponds to a free-energy difference of 2 kJ/mol in water and 2.6 kJ/mol for the water cage around an argon atom (82), we can conclude that the structure of noncoordinated water in the biomolecular condensate hardly differs from the one in bulk water. LLPS involves a minor increase of the free energy of water in favor of a decrease of free energy of the protein. This finding applies in general, as it directly follows from the moderately lower molar fraction of water and the higher molar fraction of protein in the biomolecular condensate.

In line with previous findings from NMR, molecular dynamics, and sequence analysis (13,19–21,72,83–85), our results suggest that a multitude of transient, weak interactions between amino acid residues are the driving force for LLPS of FUS NTD. We find no indication for persistent structure, except perhaps for a slight extension of the section 128–158 in the dispersed state. The earlier works have identified hydrogen bonding as the dominating interaction type, with $\pi - sp^2$ interactions (86) between Tyr and predominantly Gln also contributing substantially. This picture suggests partial dehydration of the protein upon LLPS, which is consistent with recent findings from Terahertz spectroscopy (47,48).

We note that the presence of folded domains and of the other two RGG domains could modify LLPS behavior for full-length FUS, which is known to undergo LLPS at lower concentrations than the QGSY-rich region on its own (85). A recent publication demonstrated that the protein compacts as it approaches LLPS (87), but the overall conformation of the NTD in this context is not yet known. The fIDPnn disorder prediction shown in Fig. 1 *B* indicates that the last 30 residues of the RGG1 domain may exhibit substantial order in the context of the RNA-recognition motif (285–371), although AlphaFold2 pLDDT is in contrast to this prediction. For the dispersed state of hnRNP A1, we found interaction of the Gly-rich disordered domain with the folded RNA-recognition motifs, suggesting that displacement of the Gly-rich domain from the RNA-recognition motifs by RNA could trigger LLPS (42). Even considering such modifications in LLPS behavior due to the presence of folded domains, our results demonstrate that the basic features of LLPS caused by IDRs of proteins can be understood by established concepts from polymer physics.

CONCLUSIONS

For FUS NTD, consisting of the QGSY-rich domain and the RGG1 domain, we have obtained ensemble models based on distance distribution restraints for the denatured, dispersed, and condensed state. Both in the dispersed and in the condensed state, FUS NTD can be described to a good approximation as a random-coil block copolymer in a good solvent. This explains the slight compaction of FUS NTD upon LLPS that we observed for all 10 sections tested, since random coils in good solvent contract upon an increase of polymer concentration (73,79) as a consequence of de Gennes' theory (78). The concentration of FUS NTD in the biomolecular condensate exceeds the overlap concentration of the random coil. The QGSY-rich block is slightly more extended and predicted to be slightly more hydrated and have a slightly larger propensity to LLPS than the RGG block. In the condensed state, chain extension is nearly homogeneous along the sequence, in particular for the QGSY-rich domain. In the dispersed state, a section in the range between residues 128 and 158 appears to be slightly more extended. Our results demonstrate that the formation of biomolecular condensates at physiologically relevant protein concentrations is consistent with full disorder and with good solvation of protein domains in both the dispersed and condensed state.

SUPPORTING MATERIAL

Supporting material can be found online at https://doi.org/10.1016/j.bpj.2024.01.023.

AUTHOR CONTRIBUTIONS

L.E.H., L.E., F.H.T.A., and G.J. designed the research. M.Y. assisted with the experimental design for EPR. L.E.H. performed and analyzed the EPR and microscopy experiments. L.E.H. and L.E. performed sample preparation and turbidity experiments. L.E.H. carried out ensemble modeling with some software support by G.J. L.E.H. and G.J. analyzed the ensembles. L.E.H. and G.J. wrote the article with input from all other authors.

ACKNOWLEDGMENTS

We thank Irina Ritsch, Daniel Nettels, and Ben Schuler for helpful discussions and an anonymous reviewer for extensive helpful comments on presentation. L.E.H. thanks Justine Kusch-Wieser for introduction into microscopy. This research was supported by the Swiss National Science Foundation with Sinergia grant no. CR-SII5_170976.

DECLARATION OF INTERESTS

The authors declare no competing interests.

REFERENCES

1. Brangwynne, C. P., C. R. Eckmann, ..., A. A. Hyman. 2009. Germline P granules are liquid droplets that localize by controlled dissolution/condensation. *Science.* 324:1729–1732.
2. Li, P., S. Banjade, ..., M. K. Rosen. 2012. Phase transitions in the assembly of multivalent signalling proteins. *Nature.* 483:336–340.

3. Courchaine, E. M., A. Lu, and K. M. Neugebauer. 2016. Droplet organelles? *EMBO J.* 35:1603–1612.
4. Hirose, T., K. Ninomiya, …, T. Yamazaki. 2023. A guide to membraneless organelles and their various roles in gene regulation. *Nat. Rev. Mol. Cell Biol.* 24:288–304.
5. Uversky, V. N. 2017. Intrinsically disordered proteins in overcrowded milieu: Membrane-less organelles, phase separation, and intrinsic disorder. *Curr. Opin. Struct. Biol.* 44:18–30.
6. Borcherds, W., A. Bremer, …, T. Mittag. 2021. How do intrinsically disordered protein regions encode a driving force for liquid-liquid phase separation? *Curr. Opin. Struct. Biol.* 67:41–50.
7. Elbaum-Garfinkle, S., Y. Kim, …, C. P. Brangwynne. 2015. The disordered P granule protein LAF-1 drives phase separation into droplets with tunable viscosity and dynamics. *Proc. Natl. Acad. Sci. USA.* 112:7189–7194.
8. Nott, T. J., E. Petsalaki, …, A. J. Baldwin. 2015. Phase transition of a disordered nuage protein generates environmentally responsive membraneless organelles. *Mol. Cell.* 57:936–947.
9. Brangwynne, C., P. Tompa, and R. Pappu. 2015. Polymer physics of intracellular phase transitions. *Nat. Phys.* 11:899–904.
10. Ranganathan, S., and E. Shakhnovich. 2022. The physics of liquid-to-solid transitions in multi-domain protein condensates. *Biophys. J.* 121:2751–2766.
11. Shin, Y., and C. P. Brangwynne. 2017. Liquid phase condensation in cell physiology and disease. *Science.* 357, eaaf4382.
12. Duggan, M., B. Torkzaban, …, J. Gordon. 2020. Age-related neurodegenerative diseases. *J. Cell. Physiol.* 235:3131–3141.
13. Alberti, S., S. Saha, …, A. A. Hyman. 2018. A User's Guide for Phase Separation Assays with Purified Proteins. *J. Mol. Biol.* 430:4806–4820.
14. Alberti, S., A. Gladfelter, and T. Mittag. 2019. Considerations and challenges in studying liquid-liquid phase separation and biomolecular condensates. *Cell.* 176:419–434.
15. Peran, I., and T. Mittag. 2020. Molecular structure in biomolecular condensates. *Curr. Opin. Struct. Biol.* 60:17–26.
16. Emmanouilidis, L., L. Esteban-Hofer, …, F. H.-T. Allain. 2021. NMR and EPR reveal a compaction of the RNA-binding protein FUS upon droplet formation. *Nat. Chem. Biol.* 17:608–614.
17. Lagier-Tourenne, C., M. Polymenidou, and D. W. Cleveland. 2010. TDP-43 and FUS/TLS: Emerging roles in RNA processing and neurodegeneration. *Hum. Mol. Genet.* 19:46–64.
18. Sama, R. R. K., C. L. Ward, and D. A. Bosco. 2014. Functions of FUS/TLS from DNA repair to stress response: implications for ALS. *ASN neuro.* 6, 1759091414544472.
19. Burke, K. A., A. M. Janke, …, N. L. Fawzi. 2015. Residue-by-residue view of in vitro FUS granules that bind the C-terminal domain of RNA polymerase II. *Mol. Cell.* 60:231–241.
20. Murthy, A. C., G. L. Dignon, …, N. L. Fawzi. 2019. Molecular interactions underlying liquid-liquid phase separation of the FUS low-complexity domain. *Nat. Struct. Mol. Biol.* 26:637–648.
21. Wang, J., J.-M. Choi, …, A. A. Hyman. 2018. A molecular grammar governing the driving forces for phase separation of prion-like RNA binding proteins. *Cell.* 174:688–699.e16.
22. Kang, J., L. Lim, …, J. Song. 2019. A unified mechanism for LLPS of ALS/FTLD-causing FUS as well as its modulation by ATP and oligonucleic acids. *PLoS Biol.* 17, e3000327.
23. Hu, G., A. Katuwawala, …, L. Kurgan. 2021. flDPnn: Accurate intrinsic disorder prediction with putative propensities of disorder functions. *Nat. Commun.* 12:4438.
24. Necci, M., D. Piovesan, …, D. Curators. 2021. Critical assessment of protein intrinsic disorder prediction. *Nat. Methods.* 18:472–481.
25. Jumper, J., R. Evans, …, D. Hassabis. 2021. Highly accurate protein structure prediction with AlphaFold. *Nature.* 596:583–589.
26. Tunyasuvunakool, K., J. Adler, …, D. Hassabis. 2021. Highly accurate protein structure prediction for the human proteome. *Nature.* 596:590–596.
27. Ritsch, I., L. Esteban-Hofer, …, G. Jeschke. 2021. Characterization of weak protein domain structure by spin-label distance distributions. *Front. Mol. Biosci.* 8, 636599.
28. Jeschke, G. 2016. Ensemble models of proteins and protein domains based on distance distribution restraints: Ensembles by Distance Distribution Restraints. *Proteins.* 84:544–560.
29. Jeschke, G., and L. Esteban-Hofer. 2022. Integrative ensemble modeling of proteins and their complexes with distance distribution restraints. *Methods Enzymol.* 666:145–169.
30. Zhang, M., B. Xue, …, J. Li. 2023. Sequence tendency for the interaction between low-complexity intrinsically disordered proteins. *JACS Au.* 3:93–104.
31. Polyhach, Y., E. Bordignon, …, G. Jeschke. 2012. High sensitivity and versatility of the DEER experiment on nitroxide radical pairs at Q-band frequencies. *Phys. Chem. Chem. Phys.* 14:10762–10773.
32. Doll, A., S. Pribitzer, …, G. Jeschke. 2013. Adiabatic and fast passage ultra-wideband inversion in pulsed EPR. *J. Magn. Reson.* 230:27–39.
33. Breitgoff, F. D., J. Soetbeer, …, Y. O. Polyhach. 2017. Artefact suppression in 5-pulse double electron electron resonance for distance distribution measurements. *Phys. Chem. Chem. Phys.* 19:15766–15779.
34. Fábregas Ibáñez, L., G. Jeschke, and S. Stoll. 2020. DeerLab: a comprehensive software package for analyzing dipolar electron paramagnetic resonance spectroscopy data. *Magnes. Res.* 1:209–224.
35. Brandon, S., A. H. Beth, and E. J. Hustedt. 2012. The global analysis of DEER data. *J. Magn. Reson.* 218:93–104.
36. Krivov, G. G., M. V. Shapovalov, and R. L. Dunbrack. 2009. Improved prediction of protein side-chain conformations with SCWRL4. *Proteins.* 77:778–795.
37. Rudnick, J., and G. Gaspari. 1986. The asphericity of random walks. *J. Phys. Math. Gen.* 19:L191–L193.
38. Kanchi, S., G. Suresh, …, P. K. Maiti. 2015. Molecular dynamics study of the structure, flexibility, and hydrophilicity of PETIM dendrimers: A comparison with PAMAM dendrimers. *J. Phys. Chem. B.* 119:12990–13001.
39. Briones, R., C. Blau, …, C. Aponte-Santamaría. 2019. GROmaρs: A GROMACS-based toolset to analyze density maps derived from molecular dynamics simulations. *Biophys. J.* 116:4–11.
40. Gallivan, J. P., and D. A. Dougherty. 1999. Cation-pi interactions in structural biology. *Proc. Natl. Acad. Sci. USA.* 96:9459–9464.
41. Huang, P., H. Xing, …, J. Fan. 2021. Accurate prediction of hydration sites of proteins using energy model with atom embedding. *Front. Mol. Biosci.* 8, 756075.
42. Ritsch, I., E. Lehmann, …, G. Jeschke. 2022. Phase separation of heterogeneous nuclear ribonucleoprotein A1 upon specific RNA-binding observed by magnetic resonance. *Angew. Chem., Int. Ed. Engl.* 61, e202204311.
43. Schiemann, O., C. A. Heubach, …, G. Jeschke. 2021. Benchmark test and guidelines for DEER/PELDOR experiments on nitroxide-labeled biomolecules. *J. Am. Chem. Soc.* 143:17875–17890.
44. Shirota, H., and K. Horie. 2004. Deuterium substitution and fluorescence studies on polymer hydrogels and complexes. *Macromol. Symp.* 207:79–94.
45. Banc, A., C. Charbonneau, …, L. Ramos. 2016. Small angle neutron scattering contrast variation reveals heterogeneities of interactions in protein gels. *Soft Matter.* 12:5340–5352.
46. Price, D. L., L. Fu, …, M.-L. Saboungi. 2013. Hydrogen/deuterium isotope effects in water and aqueous solutions of organic molecules and proteins. *Chem. Phys.* 424:62–69.
47. Ahlers, J., E. M. Adams, …, M. Havenith. 2021. The key role of solvent in condensation: Mapping water in liquid-liquid phase-separated FUS. *Biophys. J.* 120:1266–1275.
48. Pezzotti, S., B. König, …, M. Havenith. 2023. Liquid-liquid phase separation? Ask the water. *J. Phys. Chem. Lett.* 14:1556–1563.

49. Borbat, P. P., E. R. Georgieva, and J. H. Freed. 2013. Improved sensitivity for long-distance measurements in biomolecules: Five-pulse double electron-electron resonance. *J. Phys. Chem. Lett.* 4:170–175.
50. Jeschke, G. 2022. Integration of nanometer-range label-to-label distances and their distributions into modelling approaches. *Biomolecules.* 12:1369.
51. Polyhach, Y., E. Bordignon, and G. Jeschke. 2011. Rotamer libraries of spin labelled cysteines for protein studies. *Phys. Chem. Chem. Phys.* 13:2356–2366.
52. Deng, H., K. Gao, and J. Jankovic. 2014. The role of FUS gene variants in neurodegenerative diseases. *Nat. Rev. Neurol.* 10:337–348.
53. Ozdilek, B. A., V. F. Thompson, …, J. C. Schwartz. 2017. Intrinsically disordered RGG/RG domains mediate degenerate specificity in RNA binding. *Nucleic Acids Res.* 45:7984–7996.
54. Murray, D. T., M. Kato, …, R. Tycko. 2017. Structure of FUS protein fibrils and its relevance to self-assembly and phase separation of low-complexity domains. *Cell.* 171:615–627.e16.
55. Hughes, M. P., M. R. Sawaya, …, D. S. Eisenberg. 2018. Atomic structures of low-complexity protein segments reveal kinked β sheets that assemble networks. *Science.* 359:698–701.
56. Luo, F., X. Gui, …, C. Liu. 2018. Atomic structures of FUS LC domain segments reveal bases for reversible amyloid fibril formation. *Nat. Struct. Mol. Biol.* 25:341–346.
57. Lee, M., U. Ghosh, …, R. Tycko. 2020. Molecular structure and interactions within amyloid-like fibrils formed by a low-complexity protein sequence from FUS. *Nat. Commun.* 11:5735.
58. Kar, M., A. E. Posey, …, R. V. Pappu. 2021. Glycine-rich peptides from FUS have an intrinsic ability to self-assemble into fibers and networked fibrils: Published as part of the *Biochemistry* virtual special issue "Protein Condensates". *Biochemistry.* 60:3213–3222.
59. Yu, Y. G., T. E. Thorgeirsson, and Y. K. Shin. 1994. Topology of an amphiphilic mitochondrial signal sequence in the membrane-inserted state: a spin labeling study. *Biochemistry.* 33:14221–14226.
60. Fitzkee, N. C., and G. D. Rose. 2004. Reassessing random-coil statistics in unfolded proteins. *Proc. Natl. Acad. Sci. USA.* 101:12497–12502.
61. Ticozzi, N., V. Silani, …, J. E. Landers. 2009. Analysis of FUS gene mutation in familial amyotrophic lateral sclerosis within an Italian cohort. *Neurology.* 73:1180–1185.
62. Nomura, T., S. Watanabe, …, Y. Furukawa. 2014. Intranuclear aggregation of mutant FUS/TLS as a molecular pathomechanism of amyotrophic lateral sclerosis. *J. Biol. Chem.* 289:1192–1202.
63. Le Guillou, J. C., and J. Zinn-Justin. 1977. Critical exponents for the n-vector model in three dimensions from field theory. *Phys. Rev. Lett.* 39:95–98.
64. Zheng, W., G. H. Zerze, …, R. B. Best. 2018. Inferring properties of disordered chains from FRET transfer efficiencies. *J. Chem. Phys.* 148, 123329.
65. Crick, S. L., M. Jayaraman, …, R. V. Pappu. 2006. Fluorescence correlation spectroscopy shows that monomeric polyglutamine molecules form collapsed structures in aqueous solutions. *Proc. Natl. Acad. Sci. USA.* 103:16764–16769.
66. Mukhopadhyay, S., R. Krishnan, …, A. A. Deniz. 2007. A natively unfolded yeast prion monomer adopts an ensemble of collapsed and rapidly fluctuating structures. *Proc. Natl. Acad. Sci. USA.* 104:2649–2654.
67. Tran, H. T., A. Mao, and R. V. Pappu. 2008. Role of backbone-solvent interactions in determining conformational equilibria of intrinsically disordered proteins. *J. Am. Chem. Soc.* 130:7380–7392.
68. Sasmal, S., J. Lincoff, and T. Head-Gordon. 2017. Effect of a paramagnetic spin label on the intrinsically disordered peptide ensemble of amyloid-β. *Biophys. J.* 113:1002–1011.
69. Pavlova, A., C.-Y. Cheng, …, S. Han. 2016. Protein structural and surface water rearrangement constitute major events in the earliest aggregation stages of tau. *Proc. Natl. Acad. Sci. USA.* 113:E127–E136.
70. Šolc, K. 1971. Shape of a random-flight chain. *J. Chem. Phys.* 55:335–344.
71. Virtanen, J. J., L. Makowski, …, K. F. Freed. 2010. Modeling the hydration layer around proteins: HyPred. *Biophys. J.* 99:1611–1619.
72. Dignon, G. L., W. Zheng, …, J. Mittal. 2018. Sequence determinants of protein phase behavior from a coarse-grained model. *PLoS Comput. Biol.* 14, e1005941.
73. Cotton, J. P., M. Nierlich, …, C. Picot. 1976. Experimental determination of the temperature–concentration diagram of flexible polymer solutions by neutron scattering. *J. Chem. Phys.* 65:1101–1108.
74. Ying, Q., and B. Chu. 1987. Overlap concentration of macromolecules in solution. *Macromolecules.* 20:362–366.
75. Ye, B., W. Shen, …, Z. Zhao. 2023. FAIRE-MS reveals mitotic retention of transcriptional regulators on a proteome-wide scale. *Faseb. J.* 37, e22724.
76. Hovmöller, S., T. Zhou, and T. Ohlson. 2002. Conformations of amino acids in proteins. *Acta Crystallogr. D Biol. Crystallogr.* 58:768–776.
77. Yokosawa, K., S. Kajimoto, …, T. Nakabayashi. 2022. Concentration quantification of the low-complexity domain of fused in sarcoma inside a single droplet and effects of solution parameters. *J. Phys. Chem. Lett.* 13:5692–5697.
78. De Gennes, P. G. 1975. Collapse of a polymer chain in poor solvents. *J. Phyique. Lett.* 36:55–57.
79. Daoud, M., and G. Jannink. 1976. Temperature-concentration diagram of polymer solutions. *J. Phys. (Paris).* 37:973–979.
80. Monahan, Z., V. H. Ryan, …, N. L. Fawzi. 2017. Phosphorylation of the FUS low-complexity domain disrupts phase separation, aggregation, and toxicity. *EMBO J.* 36:2951–2967.
81. Lin, Y.-H., J. D. Forman-Kay, and H. S. Chan. 2018. Theories for sequence-dependent phase behaviors of biomolecular condensates. *Biochemistry.* 57:2499–2508.
82. Silverstein, K. A. T., A. D. J. Haymet, and K. A. Dill. 2000. The strength of hydrogen bonds in liquid water and around nonpolar solutes. *J. Am. Chem. Soc.* 122:8037–8041.
83. Dignon, G. L., R. B. Best, and J. Mittal. 2020. Biomolecular phase separation: From molecular driving forces to macroscopic properties. *Annu. Rev. Phys. Chem.* 71:53–75.
84. Zheng, W., G. L. Dignon, …, J. Mittal. 2020. Molecular details of protein condensates probed by microsecond long atomistic simulations. *J. Phys. Chem. B.* 124:11671–11679.
85. Murthy, A. C., W. S. Tang, …, N. L. Fawzi. 2021. Molecular interactions contributing to FUS SYGQ LC-RGG phase separation and co-partitioning with RNA polymerase II heptads. *Nat. Struct. Mol. Biol.* 28:923–935.
86. Vernon, R. M., P. A. Chong, …, J. D. Forman-Kay. 2018. Pi-Pi contacts are an overlooked protein feature relevant to phase separation. *Elife.* 7, e31486.
87. Sahin, C., A. Motso, …, M. Landreh. 2023. Mass spectrometry of RNA-binding proteins during liquid–liquid phase separation reveals distinct assembly mechanisms and droplet architectures. *J. Am. Chem. Soc.* 145:10659–10668.

Biophysical Journal, Volume *123*

Supplemental information

Ensemble structure of the N-terminal domain (1–267) of FUS in a biomolecular condensate

Laura Esteban-Hofer, Leonidas Emmanouilidis, Maxim Yulikov, Frédéric H.-T. Allain, and Gunnar Jeschke

Supporting Material:

Ensemble Structure of the N-terminal Domain (1-267) of FUS in a Biomolecular Condensate

Laura Esteban-Hofer,[a] Leonidas Emmanouilidis,[b] Maxim Yulikov,[a] Frédéric H.-T. Allain,[b] Gunnar Jeschke*[a]

[a]*ETH Zurich, Department of Chemistry and Applied Biosciences, Vladimir-Prelog-Weg 2, 8093 Zurich, Switzerland* [b]*ETH Zurich, Department of Biology, Hönggerbergring 64, 8093 Zurich, Switzerland*

Supplemental methods

Visualization of electrostatic interaction potential

Semi-quantitative visualization of the electrostatic interaction potential was performed by superimposing electrical fields from point charges of charged residues. Our approach considers the pH value, taken as 7.0 in our visualizations, and the pKa values of amino acid sidegroups for computing effective charges. We localize the charge for Asp at the mean of the OD1 and OD2 atom coordinates, for Glu at the mean of the OE1 and OE2 atom coordinates, for His at the mean of the ND1 and NE1 coordinates, for Lys at the NZ atom coordinate, and for Arg at CZ atom coordinate. At all cubic grid points for which the electron density map is computed, we add the electric fields generated by all point charges, considering charge screening with the Debye length computed from the ionic strength of the buffer, taken here as 150 mM. The mean electric field from all charged residues at the displayed isosurface of the ensemble electron density determines the color. Blue corresponds to positive charge, red to negative charge, and white to neutrality. For good visibility, we set the extrema of the color scale to the largest absolute electric field value encountered at the whole isosurface.

We note that this approach is too crude for computing intermolecular interactions, where a Poisson-Boltzmann approach should be used instead. In our case, visualization provides only an intuitive picture in any case, as the weighted superposition of conformers in their inertia tensor principal axes systems is an artificial construct. Therefore, we prefer the simple approximation for the electric field sketched above and interpret the visualization with due caution.

Visualization of a cation-π interaction potential

Effective charges for Arg and Lys were computed considering their pKa values and the pH value (taken as 7.0). The contribution of Arg was weighted by 0.7088 and the one of Lys by 0.2912, thus taking into account relative interaction propensities found in[1]. Likewise, we assume relative π interaction propensities of -1 for Trp, of -0.5466 for Tyr, and of 0.3821 for Phe. The cation center coordinates were located as for the electrostatic interaction described above, whereas the π center coordinates were located at the centers of the aromatic systems of the respective sidegroups. We assumed decay of the cation-π interaction with the inverse square of the distance. The color scale is between gold (π systems) and blue (cations) with the centre at white. The propensity values corresponding to the extremes of the color scale were taken so as to obtain a similar visualization for cations as in the visualization of the electrostatic interaction potential described above.

We note that this approach can only provide an intuitive picture of the spatial distribution of cation and π interaction propensities with respect to the N and C termini of the protein.

Estimates of protein and water concentration and molar fractions

Biphasic samples were prepared with an initial concentration of 200 μM and 48% of the protein ended up in the condensed phase for wild-type FUS NTD. This puts concentration of the dispersed phase to 104 μM. Water concentration in saline buffer is approximately 55.5 M, which is by a factor of $5.34 \cdot 10^5$ larger than protein concentration. Even considering the hydration layer, we can estimate $x_{\mathrm{H_2O,disp}} \approx 1$ and $x_{\mathrm{FUS,disp}} \approx 1.04 \cdot 10^{-4}/55.5 = 1.9 \cdot 10^{-6}$.

In earlier work, we obtained 40 μL of bulk condensed FUS NTD from 80 μL of 10 mM FUS NTD stock[2]. This suggests an approximate concentration of 20 mM FUS NTD in the condensed phase, similar to the concentration of 15 mM found recently for a construct of FUS consisting of residues 1-214[3]. With the molar mass of 26295 g/mol, this corresponds to 526 mg/mL protein. Assuming a density close to 1 g/mL, we find a water concentration of about 26.9 M, which exceeds protein concentration only by a factor of the order of 1000. In this situation, water in the hydration layer[4] makes up a substantial fraction of total water. The Accutar approach[5] predicted an average of $n_{\mathrm{hydr}} = 773$ hydration water molecules per protein molecule with a standard deviation of 44. This provides a concentration of uncoordinated water of $c_{\mathrm{H_2O,free}} = 26.9\ \mathrm{M} - n_{\mathrm{hydr}} \cdot 20\ \mathrm{mM} = 11.44$ M. This value, in turn, leads to a molar fraction of uncoordinated water of $x_{\mathrm{H_2O,cond}} = 0.9983$ and a molar fraction of hydrated FUS NTD of $x_{\mathrm{FUS,cond}} = 1 - x_{\mathrm{H_2O,cond}} = 1.7 \cdot 10^{-3}$, where we neglected molar fractions of other components (salts, HEPES, urea). Note also that water beyond the first hydration shell is affected by the presence of the protein. The discussion in the main text reveals that the latter effect is minor.

Thermodynamics of LLPS

In phase equilibrium, the chemical potential of the protein is the same in both phases and the chemical potential of water is also the same in both phases. The dispersed phase is a highly diluted protein solution that we take as our reference state. For the molar chemical potential of FUS NTD in this phase, we have

$$\mu_{\mathrm{FUS,disp}} \approx \mu^{\circ}_{\mathrm{FUS}} + RT \ln x_{\mathrm{FUS,disp}} , \tag{S1}$$

where $\mu^{\circ}_{\mathrm{FUS}}$ is the chemical standard potential of hydrated FUS NTD and $x_{\mathrm{FUS,disp}}$ is the molar fraction of FUS NTD in the dispersed state, which we estimate as $x_{\mathrm{protein}} = 1.9 \cdot 10^{-6}$ (see above). Here, R is the universal gas constant and T the temperature of sample preparation that we take as 295 K.

For the condensed phase, we can write

$$\mu_{\mathrm{FUS,cond}} \approx \mu^{\circ}_{\mathrm{FUS},\infty} + RT \ln x_{\mathrm{FUS,cond}} + \Delta\mu_{\mathrm{FUS,LLPS}} \ , \tag{S2}$$

where $\Delta\mu_{\mathrm{FUS,LLPS}}$ is the change of molar chemical potential of FUS NTD due to change of intramolecular and intermolecular interactions as well as of chain conformation entropy upon transfer from the dispersed to the condensed phase. We estimate $x_{\mathrm{FUS,cond}} \approx 1.7 \cdot 10^{-3}$ (see above). This estimate is based on a concentration of FUS NTD in the biomolecular condensate of 20 mM, similar to the concentration of 15 mM found recently for a construct consisting of residues 1-214[3]. From $\mu_{\mathrm{FUS,disp}} = \mu_{\mathrm{FUS,cond}}$ at phase equilibrium, we then have

$$\Delta\mu_{\mathrm{FUS,LLPS}} \approx RT \ln \frac{x_{\mathrm{FUS,disp}}}{x_{\mathrm{FUS,cond}}} = -6.8RT = -16.5 \text{ kJ/mol} \ . \tag{S3}$$

The negative sign indicates that free energy of the hydrated protein in the condensed state at given concentration is lower than in the dispersed state.

We can make a rough estimate of the contribution of the chain conformation entropy change. To that end, we consider the solved problem of entropic elasticity of a Gaussian chain (θ-solvent conditions). Molar entropy of such a chain as a function of chain size is

$$S(R) = -\frac{3}{2} RT \frac{\vec{R}^2}{Nb^2} + S_0 \ , \tag{S4}$$

where b is the length of a Kuhn segment, N the number of Kuhn segments, and $\vec{R}$ the end-to-end distance vector. We note that $Nb^2 = R_0^2$, where R_0 is the root mean square end-to-end distance of a Gaussian chain with $\nu = 0.5$ in Eq. (10) in the main text. Hence, we substitute

$$S(R) = -\frac{3}{2} RT \frac{\vec{R}^2}{N^{2\nu} b^2} + S_0 \tag{S5}$$

and use $b = 0.51$ Å as well as $\nu = 0.592$ for the dispersed state as our reference state. We computed the ensemble averages for the dispersed and condensed state as population-weighted sums of single-chain contributions, which is in line with the derivation of Eq. (S4). In the difference, S_0 drops out

$$\Delta S(R) = -4.15R - (-3.30R) = -0.85R \ . \tag{S6}$$

The contribution to $\Delta\mu_{\mathrm{FUS,LLPS}}$ is $-T\Delta S(R) = 0.85RT$, which is almost an order of magnitude smaller in magnitude than $\Delta\mu_{\mathrm{FUS,LLPS}}$ and has opposite sign. Although this estimate is rough, it serves to demonstrate that the main contribution to $\Delta\mu_{\mathrm{FUS,LLPS}}$ comes from changes in intramolecular and intermolecular interactions, as one also expects from the sensitivity of LLPS to mutations.

By assuming that hydration of FUS NTD in the condensed states conforms to the prediction of hydration sites for isolated conformers by Accutar[5], we find for the change of molar chemical potential of water upon LLPS of wild-type FUS NTD

$$\Delta\mu_{\mathrm{H_2O,LLPS}} \approx RT \ln \frac{x_{\mathrm{H_2O,disp}}}{x_{\mathrm{H_2O,cond}}} = RT \ln \frac{1}{0.9983} = 1.7 \cdot 10^{-3} RT = 4.1 \text{ J/mol} \ . \tag{S7}$$

This small value is rather an upper bound, since the intermolecular interactions of FUS NTD molecules in the condensed phase will tend to reduce hydration.

Supplemental Figures

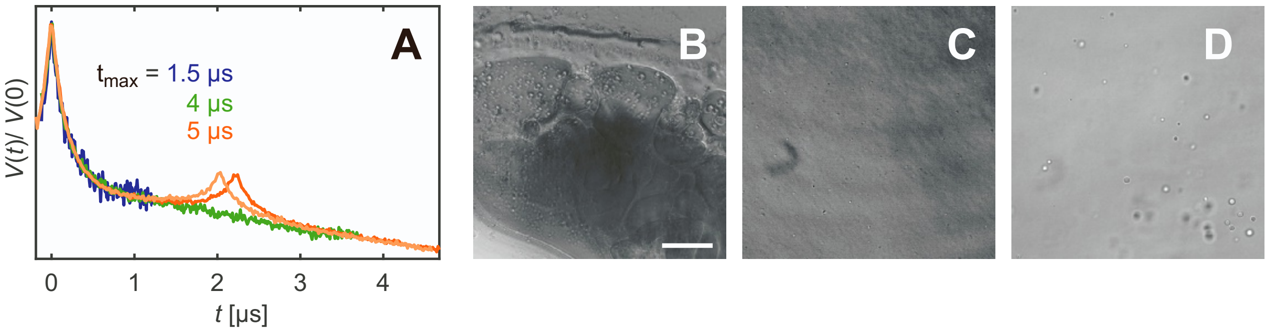

Fig. S1 5-pulse DEER and protein deuteration as potential methods to prolong DEER trace length. (A) The shape of the primary DEER data of biphasic A105C G128C remains unchanged for 4-pulse DEER traces of 1.5 µs (blue) and 4 µs (green). The same dipolar evolution is present in 5-pulse DEER traces of 5 µs with shifted artifact positions by different pulse sequence timing (two shades of orange). Traces were scaled to unit modulation depth for comparison purposes. Protein deuteration leads to unusual phase separation behavior with (B) regions that contain gel-like aggregates and (C) overall smaller (barely visible) droplets than (D) protonated FUS droplets prepared under the same conditions. Scale bar: 20 µm.

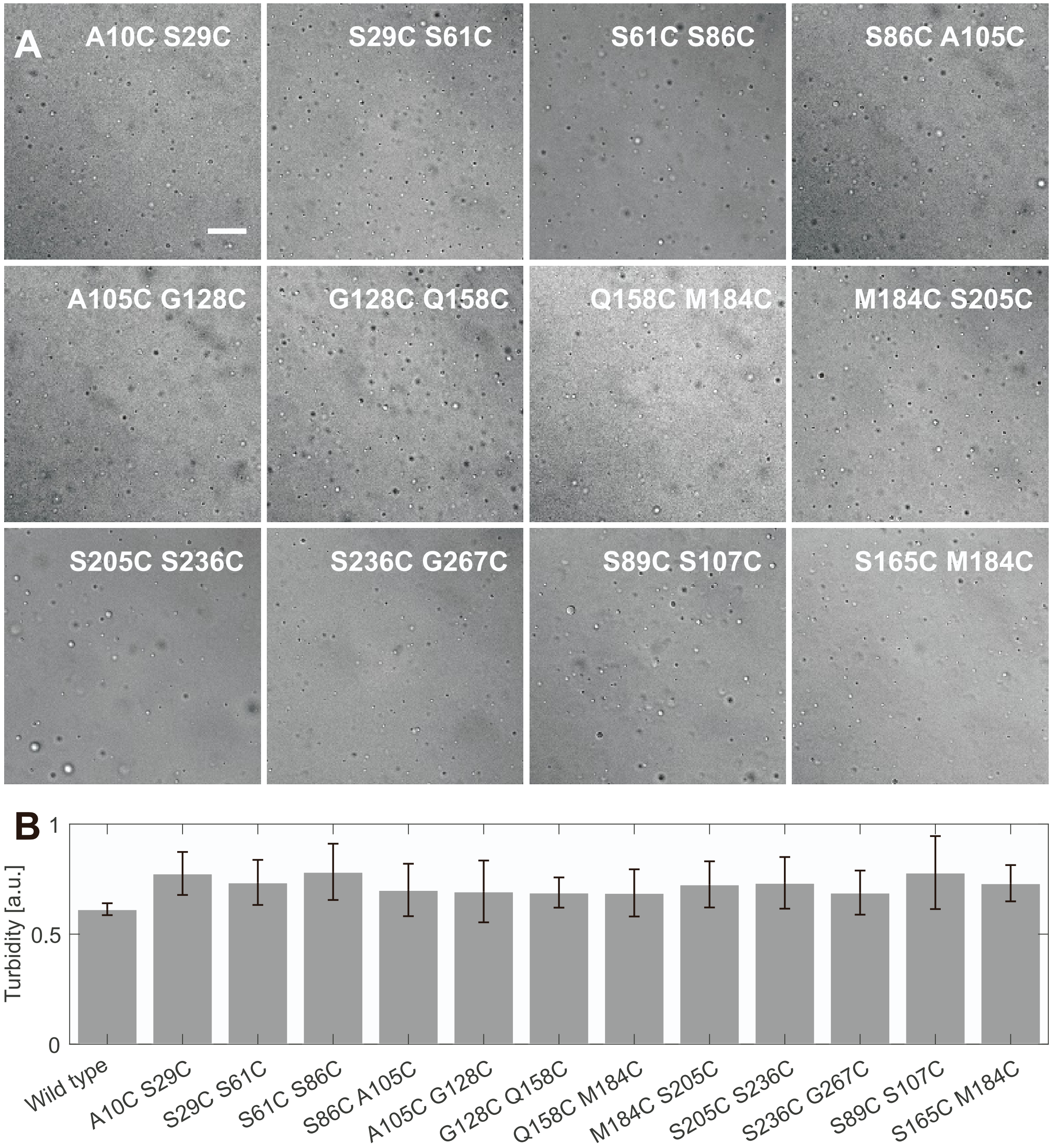

Fig. S2 Characterization of spin-labeled double cysteine mutants. (A) All double cysteine mutants retain the ability to phase separate (scale bar: 20 μm) and (B) have similar turbidity values to the ones of wild-type protein with mean and standard deviation from three independent experiments. The protein concentration employed in all experiments is 50 μM.

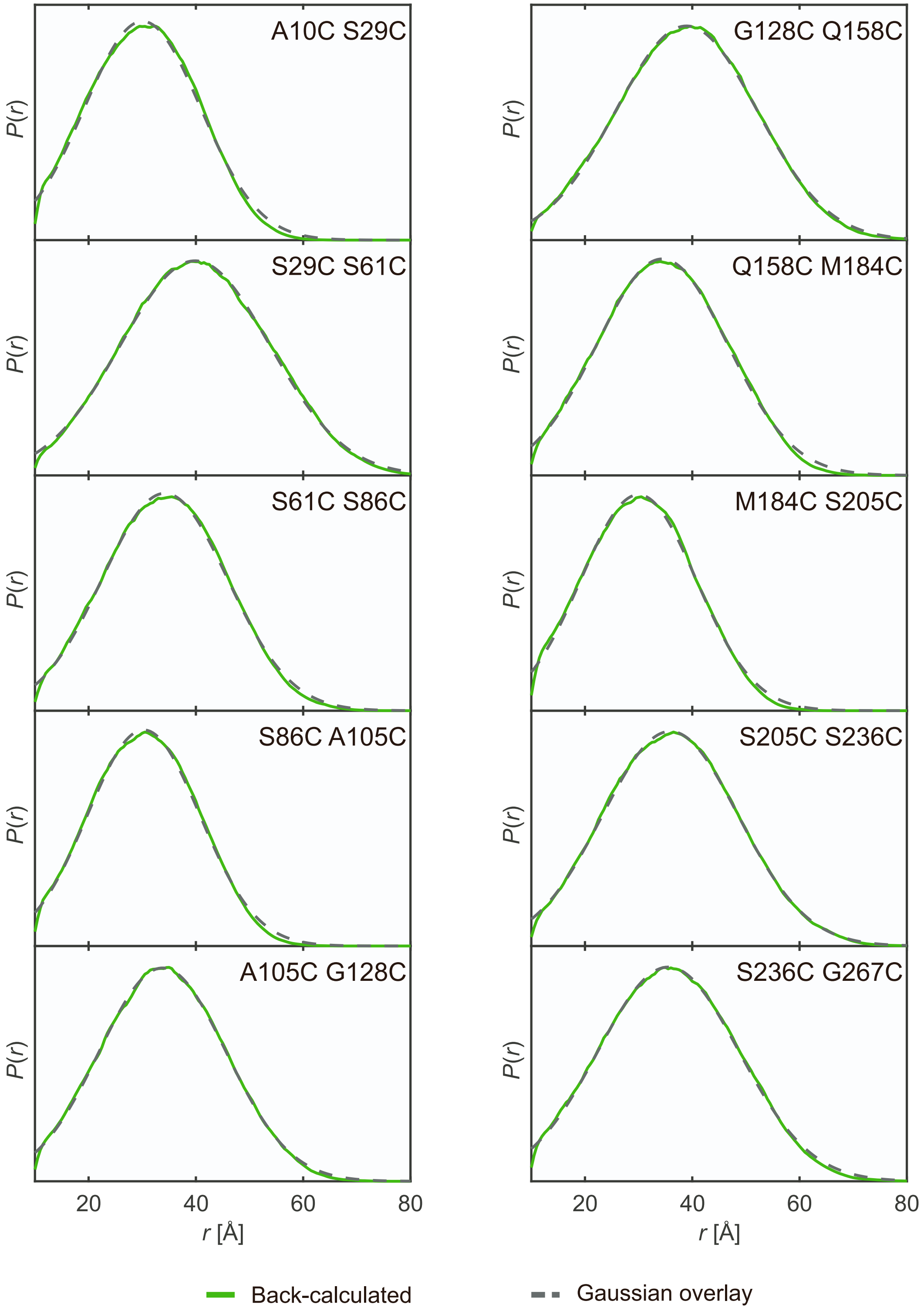

Fig. S3 Back-calculated distance distributions for the unrestrained ensemble. The back-calculated distributions (solid green line) are Gaussian-like and can be overlaid well by Gaussian fits (dashed grey line). Spin-label positions are indicated in the upper right corner of each panel.

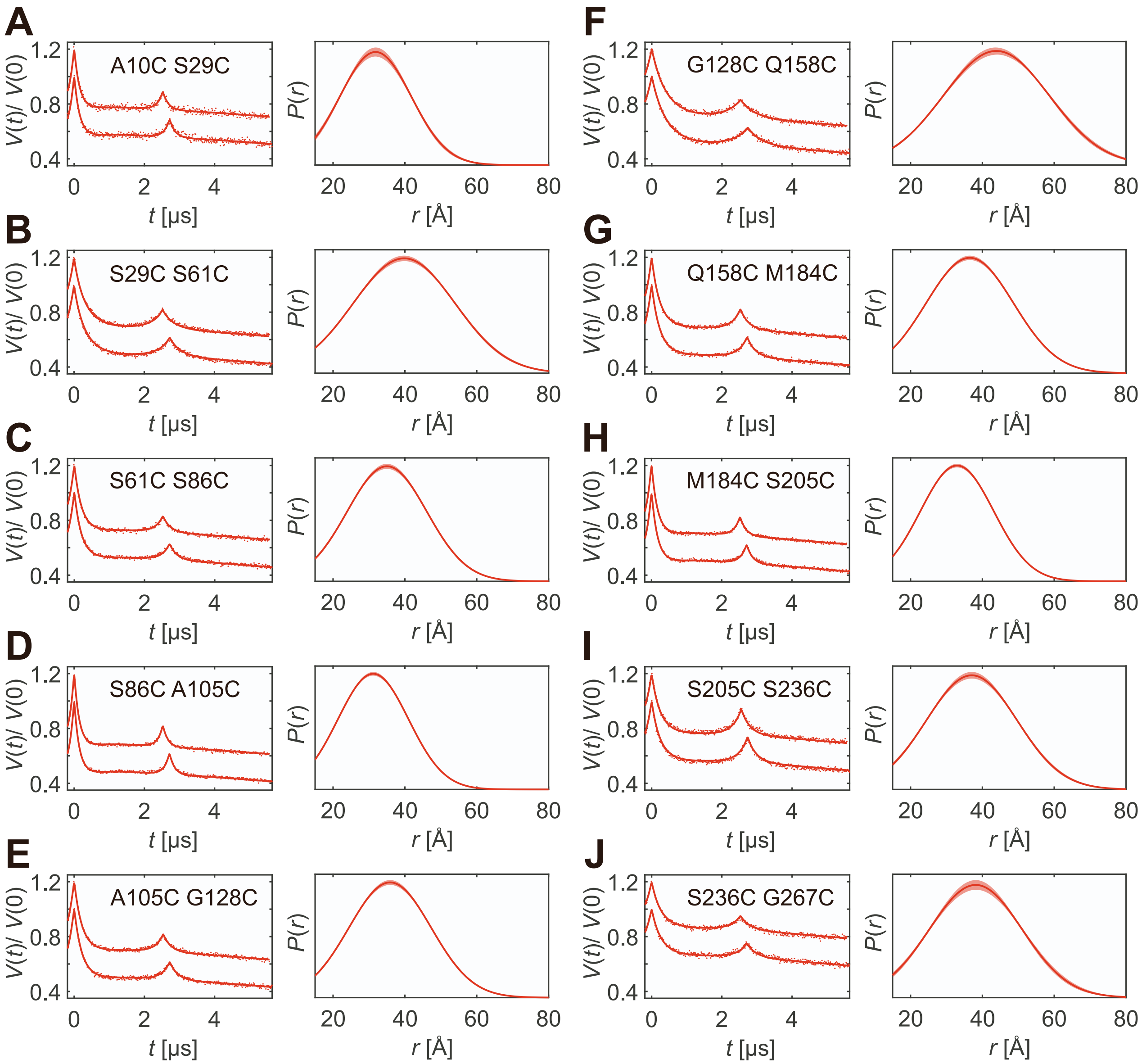

Fig. S4 5-Pulse primary DEER data of monophasic dispersed FUS. Primary DEER data (left) and corresponding distance distribution (right) of (A)-(J) A10C S29C, S29C S61C, S61C S86C, S86C A105C, A105C G128C, G128C Q158C, Q158C M184C, M184C S205C, S205C S236C, and S236C G267C, respectively. Experimental raw data are displayed as dots and Gaussian fits as solid lines with the 95% confidence intervals obtained via 1000 bootstrap samples shown as shaded area. The lower and upper 95% confidence interval of the mean and width of each distance restraint was used to confine the parameter range of the dispersed fraction in the biphasic measurement fits.

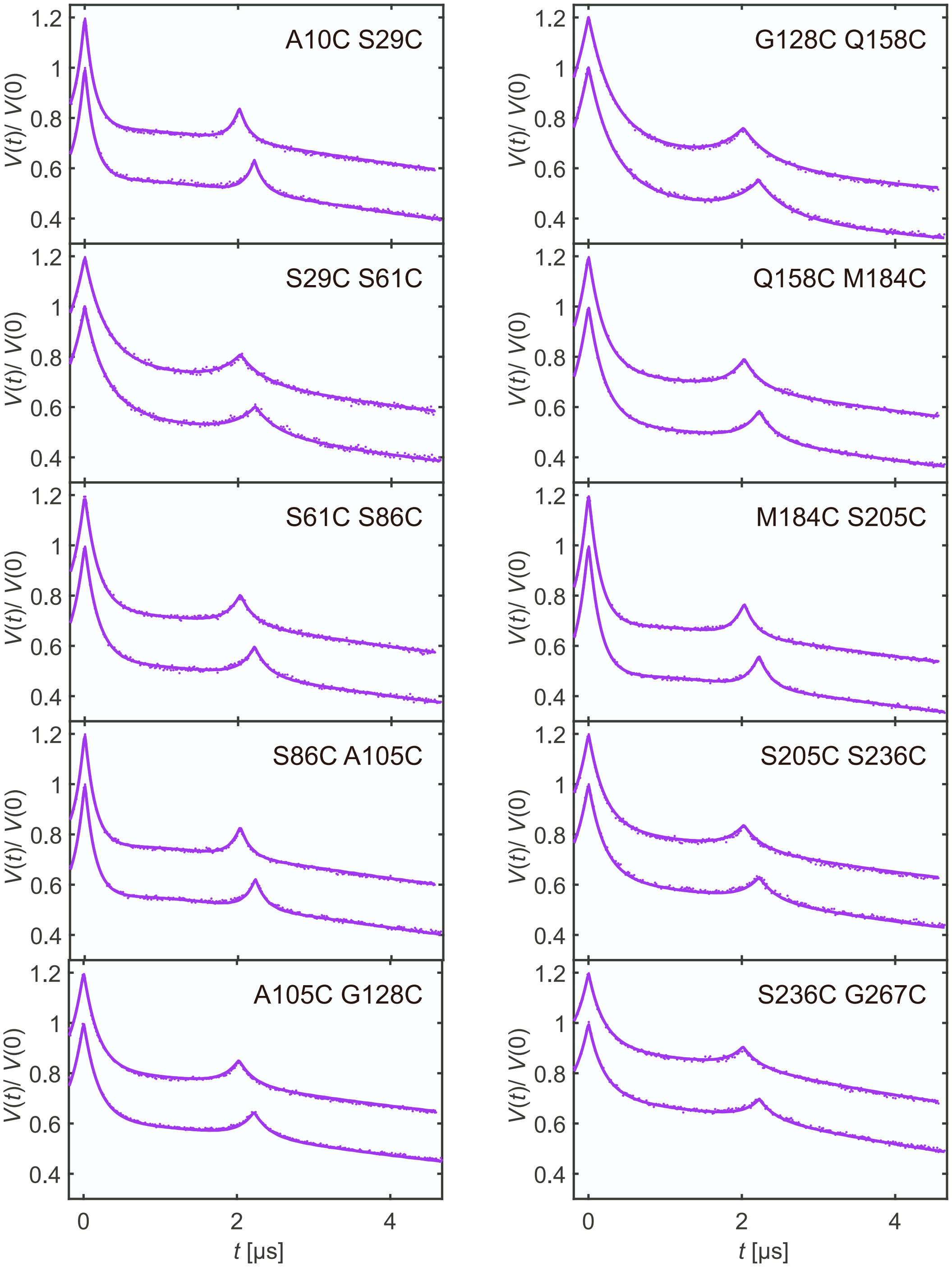

Fig. S5 5-Pulse primary DEER data of biphasic FUS. Two data sets for each mutant were measured with different pulse sequence timings. Spin-label positions are indicated in the upper right corner of each panel. Raw data are shown as violet dots, and the fits as violet lines with the 95% confidence intervals obtained via 1000 bootstrap samples shown as shaded area.

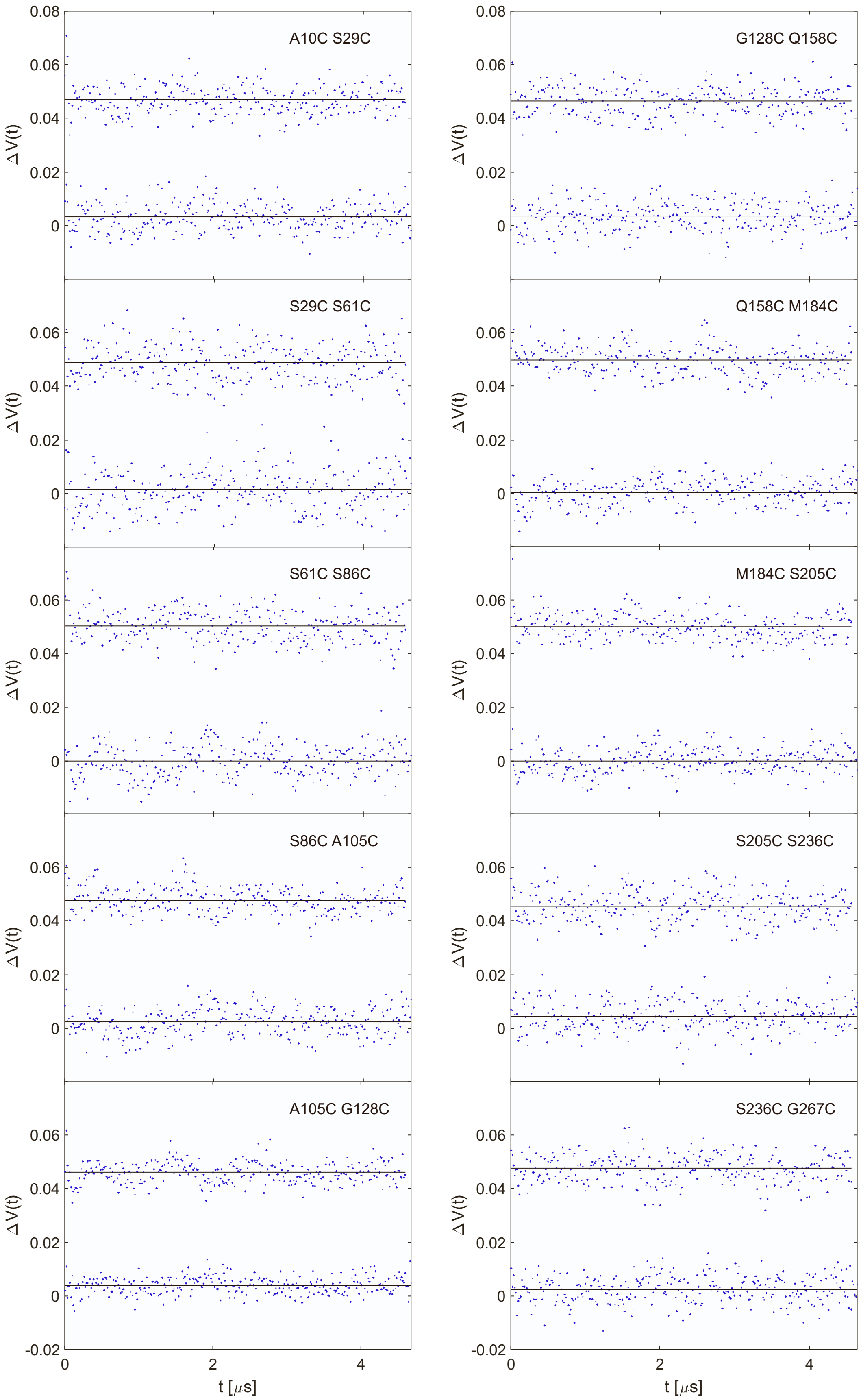

Fig. S6 Fit residuals for 5-pulse DEER data of biphasic FUS. Two data sets for each mutant were measured with different pulse sequence timings. Spin-label positions are indicated in the upper right corner of each panel. Grey horizontal lines denote the mean value of the residual.

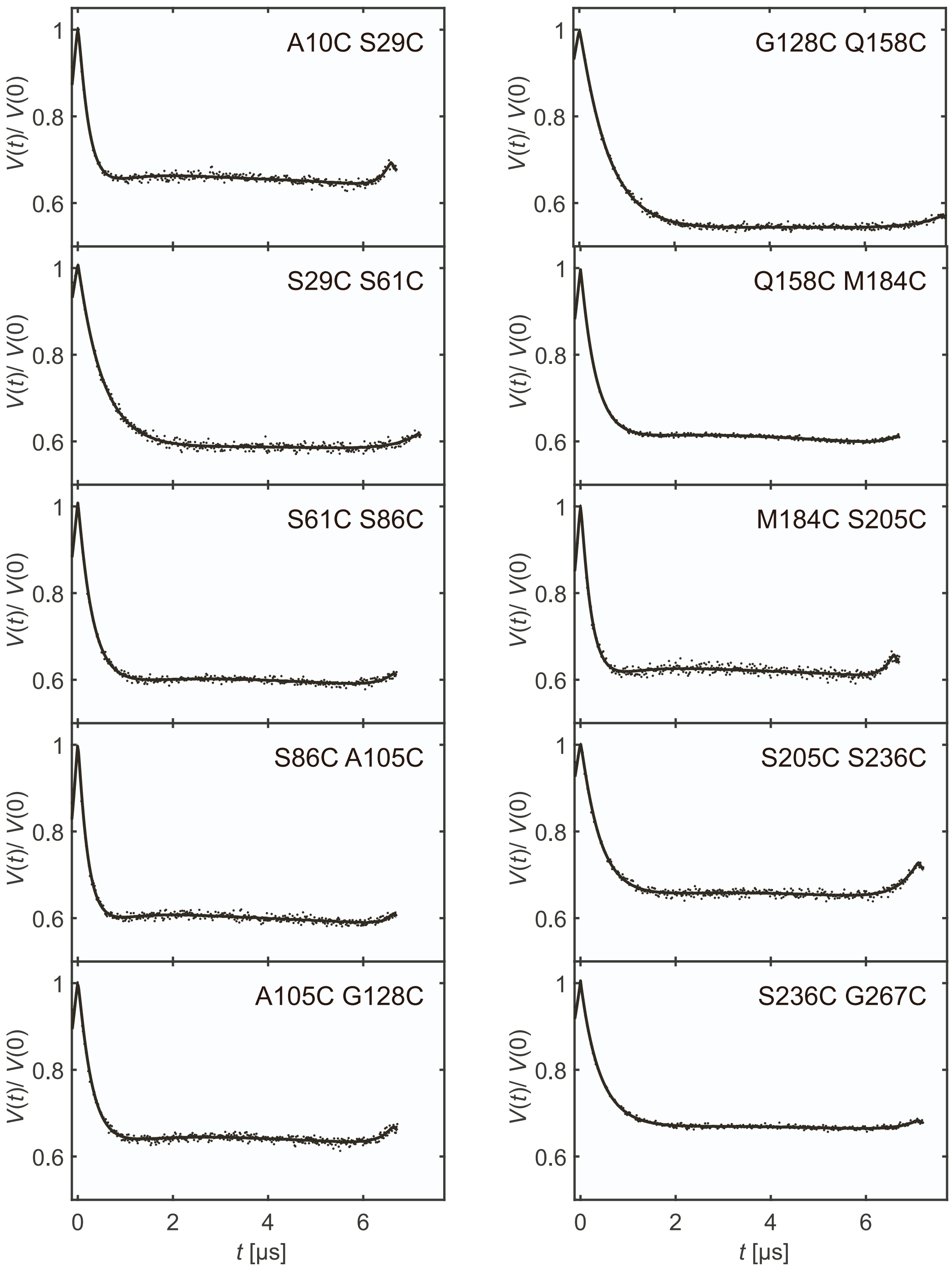

Fig. S7 Primary DEER data of FUS NTD under denaturing conditions. Spin-label positions are indicated in the upper right corner of each panel. Raw data are shown as dots, and Gaussian fits as solid lines with the 95% confidence intervals obtained via 1000 bootstrap samples shown as shaded area.

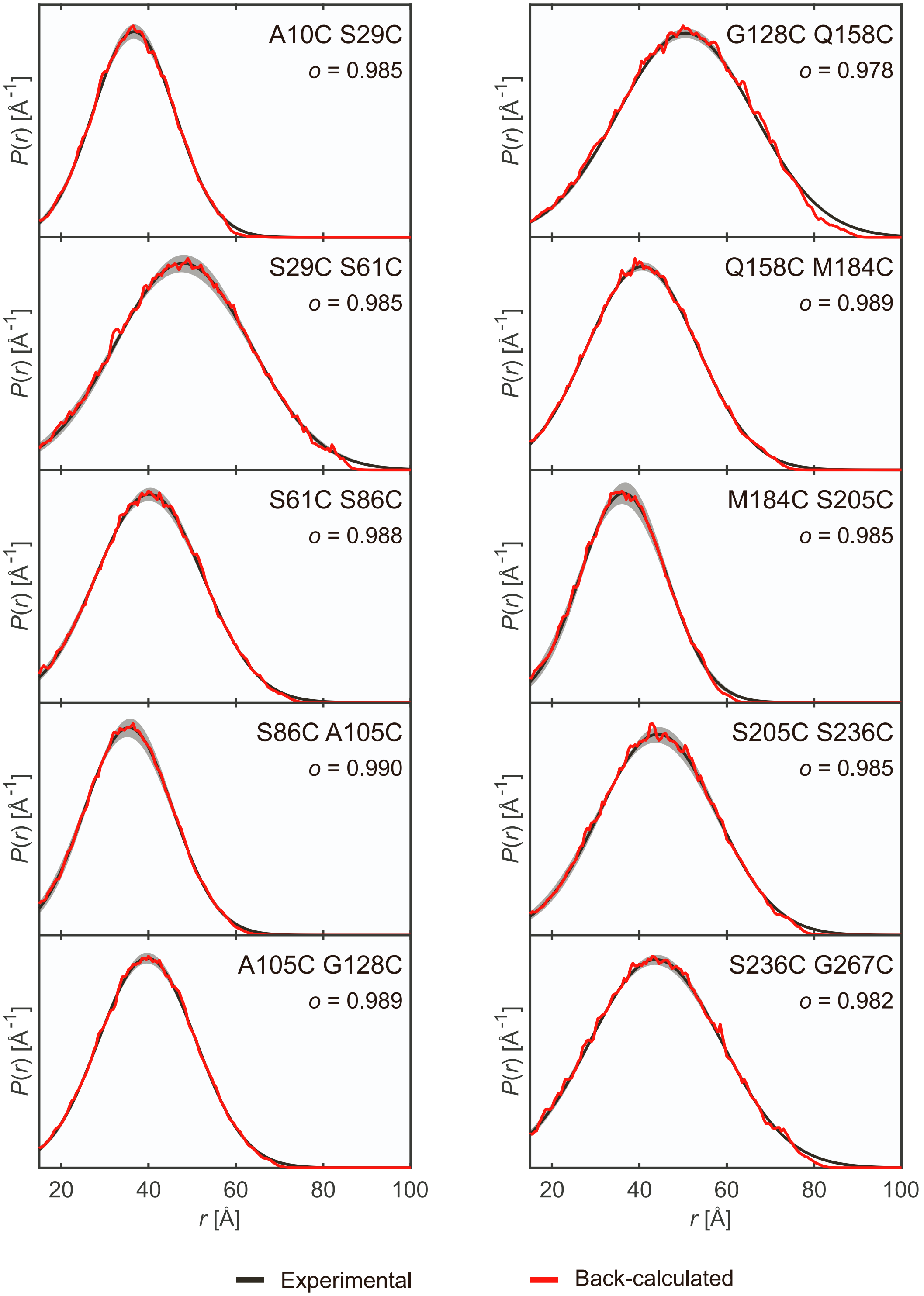

Fig. S8 Restraint fulfilment of the denatured ensemble. Back-calculated distance distributions (red) and experimental restraints (black, with shaded area for the 95% confidence interval obtained by bootstrapping with 1000 samples) are in virtually perfect agreement with each other. Spin-label positions are indicated in the upper right corner of each panel. The overlap o between the experimental and back-calculated distribution is given below.

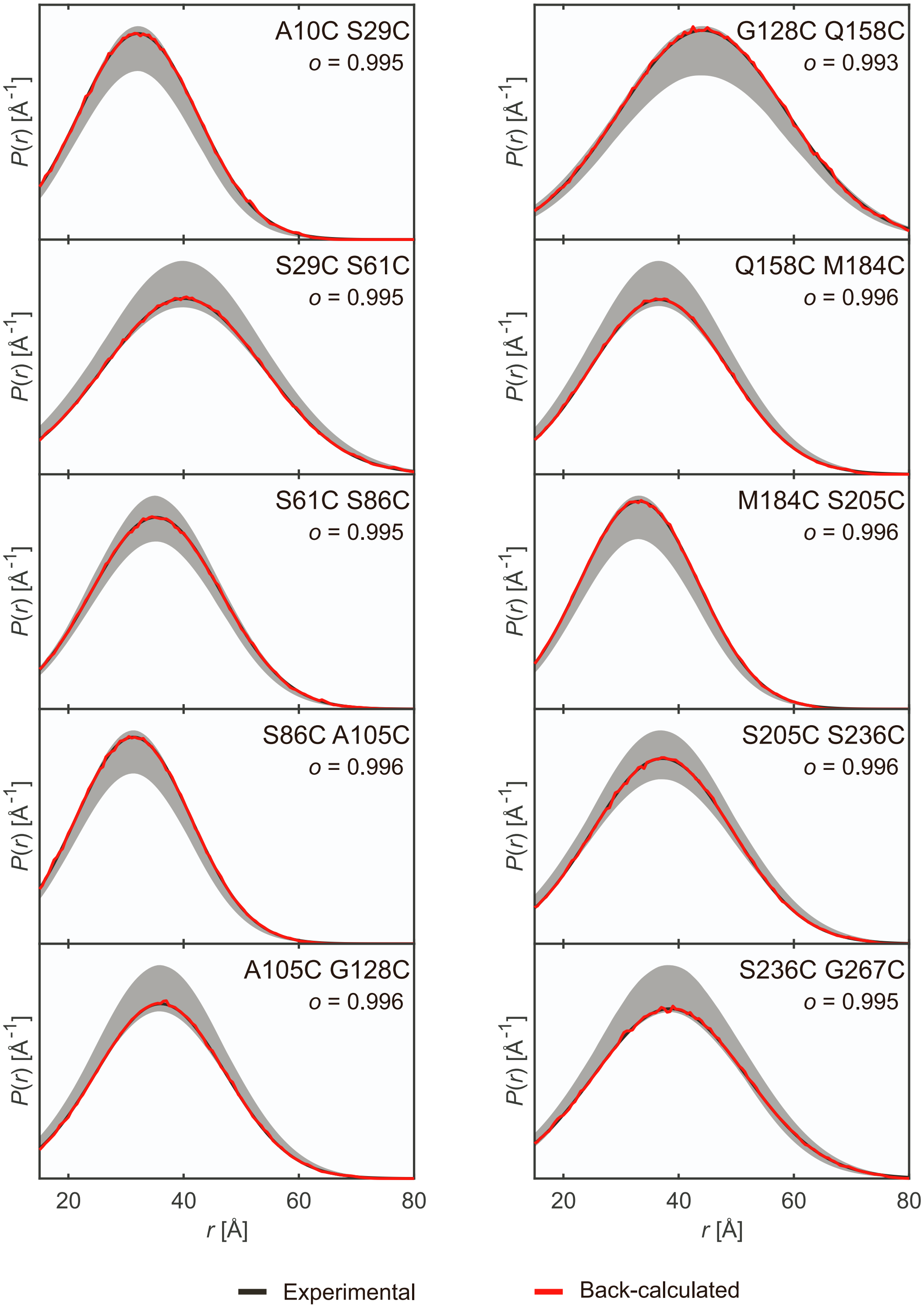

Fig. S9 Restraint fulfilment of the dispersed ensemble. Back-calculated distance distributions (red) and experimental restraints (black, with shaded area for the 95% confidence interval obtained by bootstrapping with 1000 samples) are in perfect agreement with each other. Spin-label positions are indicated in the upper right corner of each panel. The overlap o between the experimental and back-calculated distribution is given below.

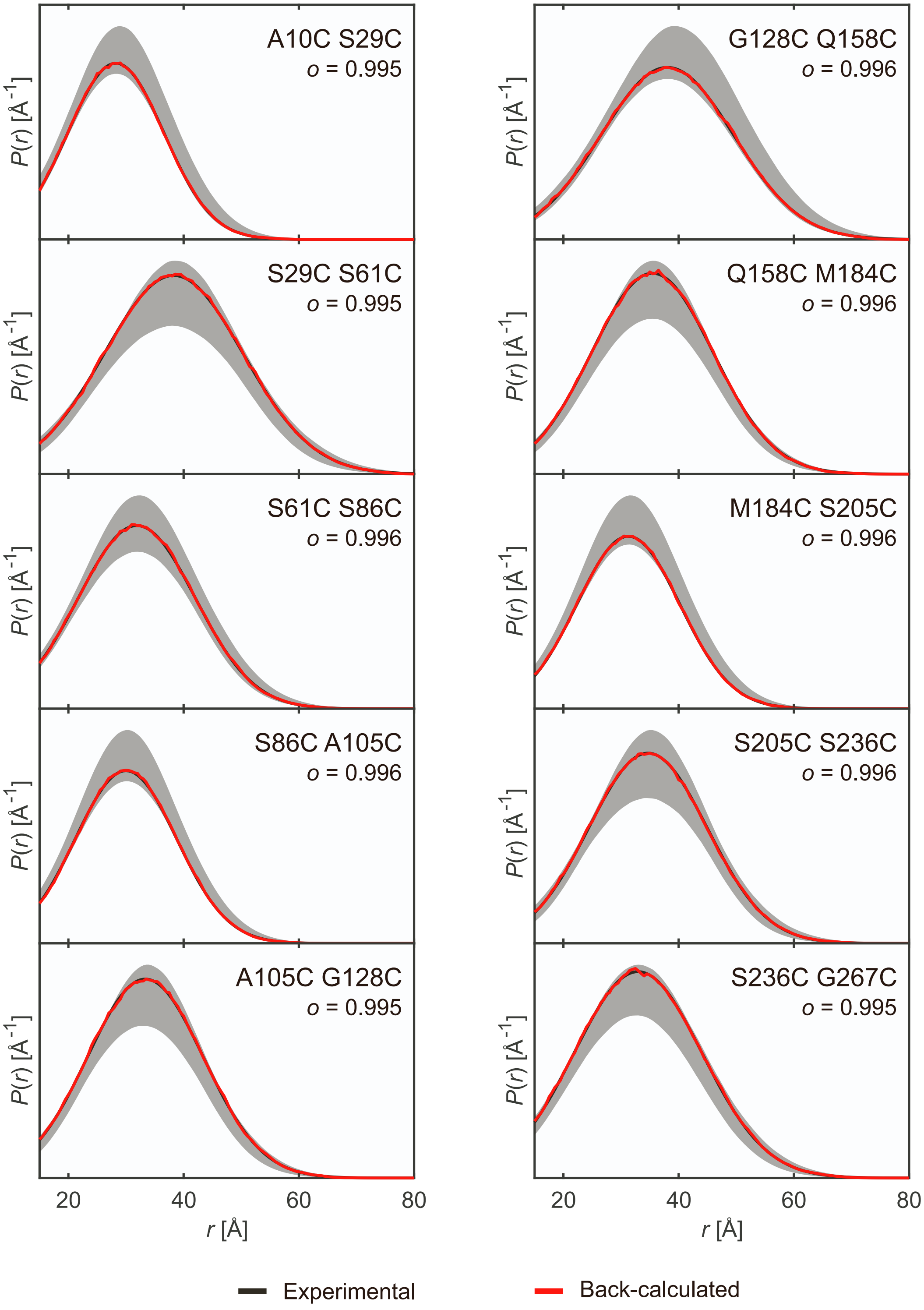

Fig. S10 Restraint fulfilment of the condensed ensemble. Back-calculated distance distributions (red) and experimental restraints (black, with shaded area for the 95% confidence interval obtained by bootstrapping with 1000 samples) are in perfect agreement with each other. Spin-label positions are indicated in the upper right corner of each panel. The overlap o between the experimental and back-calculated distribution is given below.

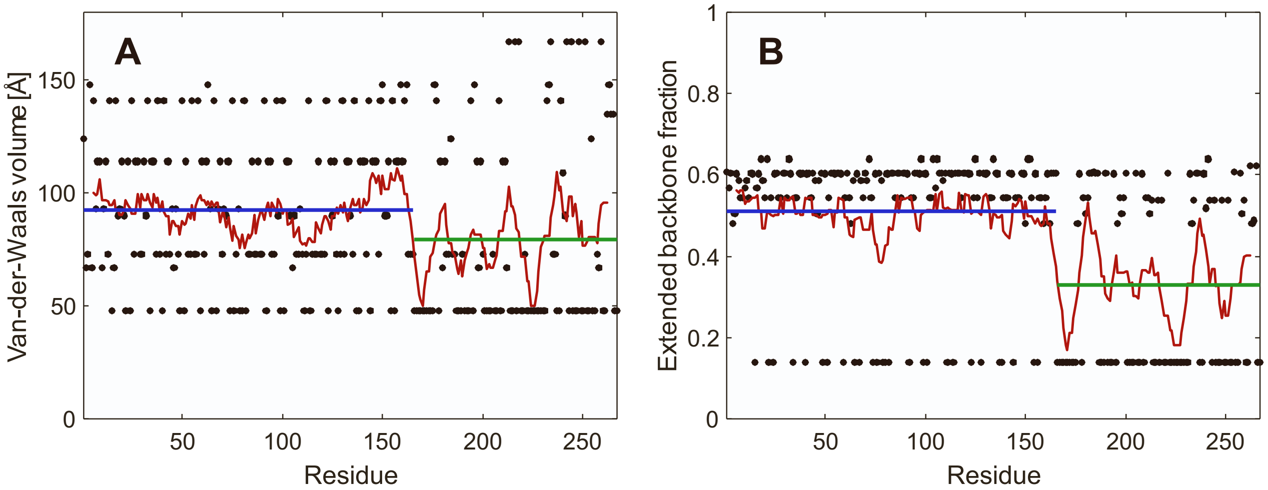

Fig. S11 Van-der-Waals volume of residues (A) and fraction of extended conformations (B) in FUS NTD. Black dots denote individual residues, crimson lines a moving average over a window of 11 residues, blue lines the mean for the QGSY-rich domain (residues 1-165), and green lines the mean for the RGG1 domain (residues 166-267). The fraction of extended conformations is defined as the population in the range $\phi = (-180^\circ, -45^\circ)$, $\psi = (45^\circ, 180^\circ)$ in residue-specific Ramachandran plots.

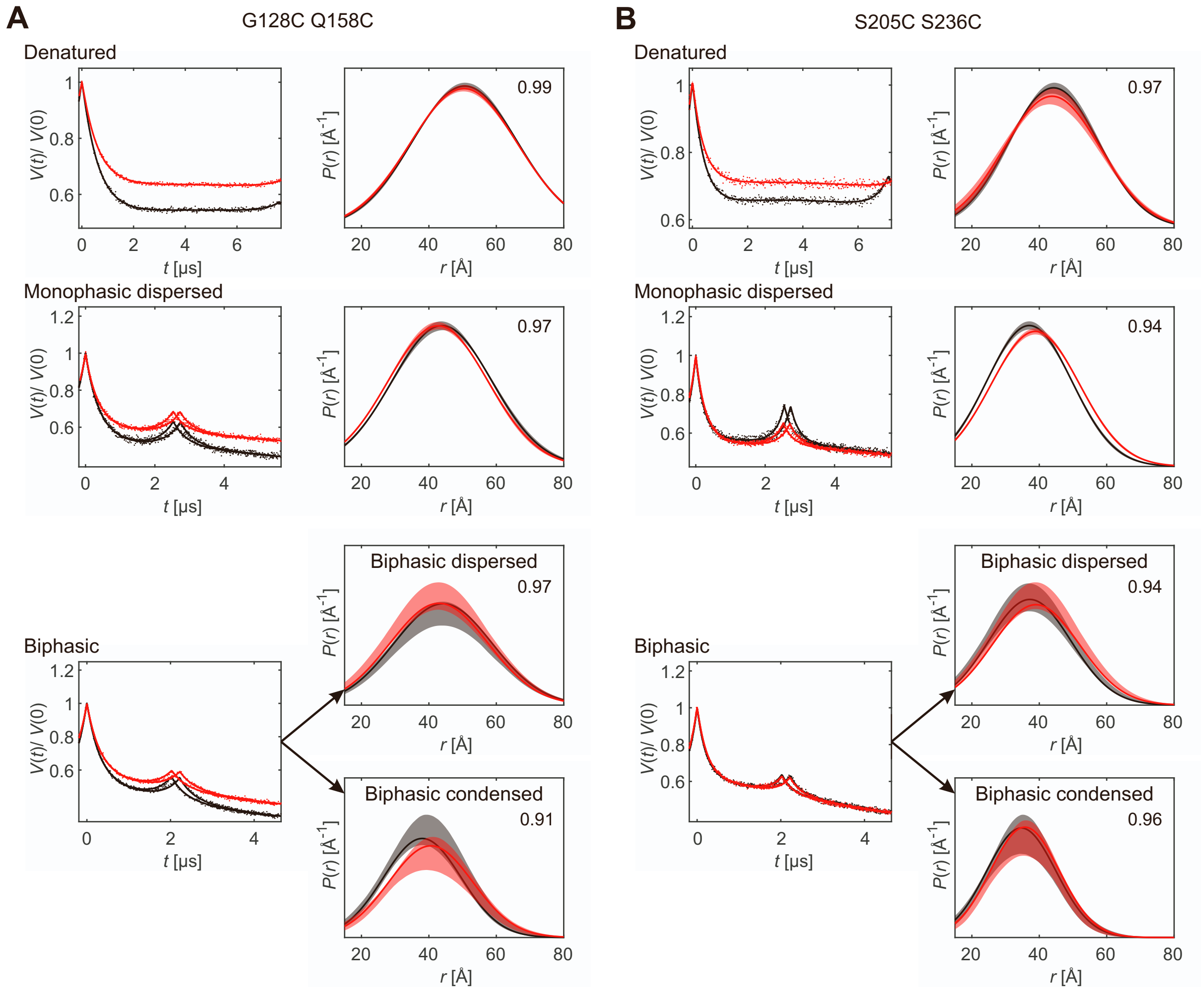

Fig. S12 Reproducibility of DEER measurements for mutants (A) G128C Q158C and (B) S205C S236C. Comparison of original (black) and reproduced (red) primary DEER data (left, raw data are shown as dots, and fits as solid lines) and corresponding distance distributions (right) in the denatured, monophasic dispersed, and biphasic states. Shaded areas correspond to the 95% confidence intervals. The overlap value between the original and reproduced distance distribution is displayed in the upper right corner of each panel. Gaussian fit parameters are reported in Table S3

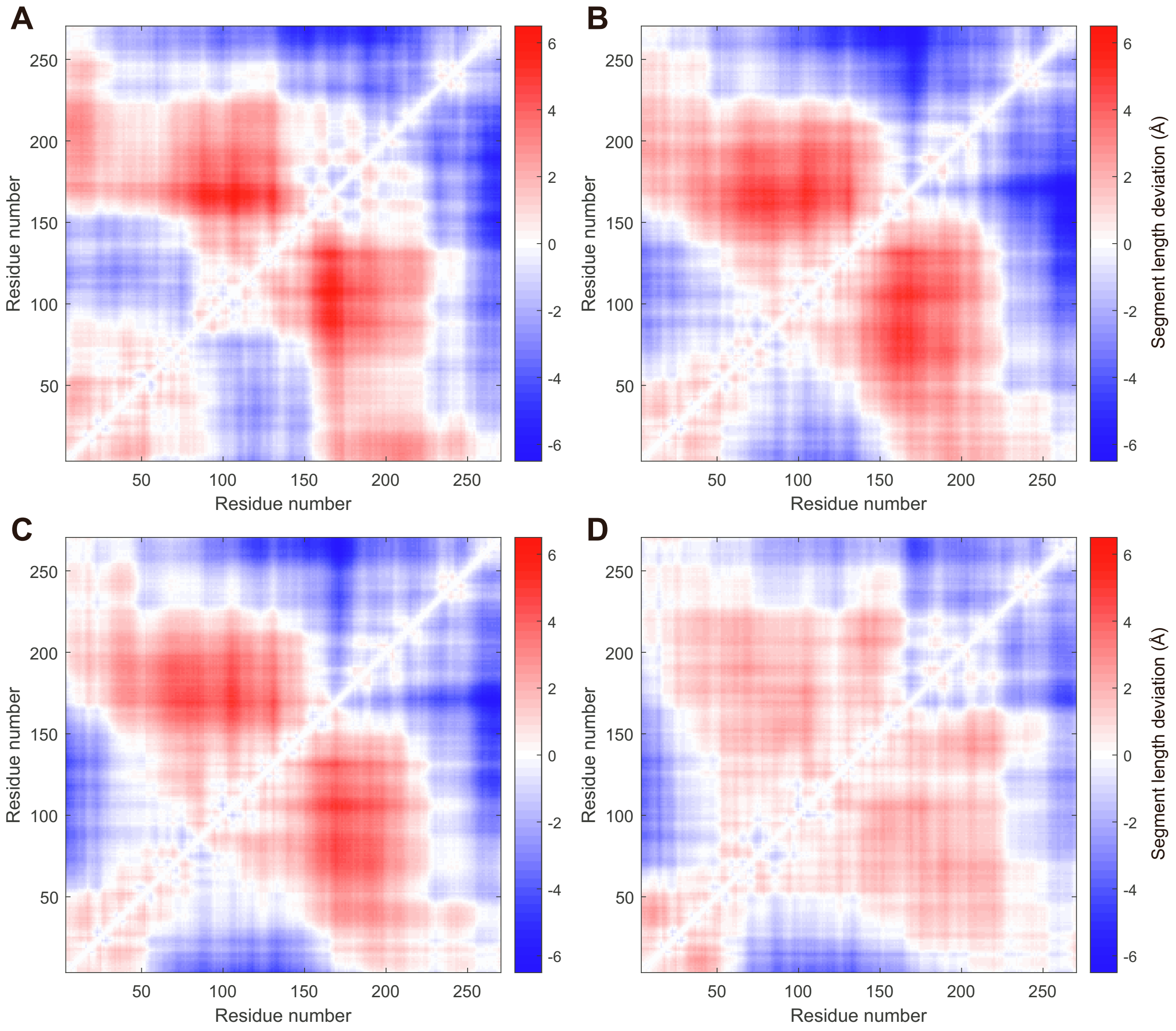

Fig. S13 Section-length deviation ΔR from the average RMS Cα-Cα distance for a given sequence separation ΔN for the dispersed state. The section-length deviations are shown for (A) the main ensemble reported in the main text (except for a slight change in color scale, same as Figure 6C), (B) an ensemble obtained with the same restraints as the main ensemble by reweighting of the unrestrained ensemble, (C) an ensemble obtained by reweighting of the unrestrained ensemble with the restraints for sections 128-158 and 205-236 by restraints obtained in technical repeats, and (D) an ensemble obtained by reweighting of the unrestrained ensemble with the restraint for section 128-158 skipped and all other restraints being the same as for the main ensemble. Each subplot is visualized with the same color axis.

Supplemental Table

Table S1 Parameter range employed for the analysis of the biphasic DEER measurements. The parameter range of the mean $\langle r \rangle_{\mathsf{disp}}$ and full width at half maximum Γ_{disp} of the biphasic dispersed state were constrained to the lower and upper 95% confidence interval (indexed with "low" and "up", respectively) of the monophasic dispersed state measured at 0.6 M urea concentration. We note that $\Gamma = 2\sqrt{2\ln 2}\sigma$ is related to the standard deviation σ only by a constant factor. Hence, restraining and fitting Γ is equivalent to restraining and fitting σ. $T_{0,2}^{(1)}$ and $T_{0,2}^{(2)}$ are the refocusing times of the additional modulated dipolar pathway of the first and second 5-pulse DEER trace, respectively. The parameter η corresponds to the fraction of protein in the dispersed phase and was constrained to $\pm$10% of the fraction determined by DOSY experiments f_{DOSY}.

	Lower	Upper
$\langle r \rangle_{\mathrm{disp}}$ [Å]	$\langle r \rangle_{\mathrm{low}}$	$\langle r \rangle_{\mathrm{up}}$
Γ_{disp} [Å]	Γ_{low}	Γ_{up}
k_{disp} [μs^{-1}]	0	0.09
$\langle r \rangle_{\mathrm{cond}}$ [Å]	10	80
Γ_{cond} [Å]	2	50
k_{cond} [μs$^{-d/3}$]	0	1
d_{cond}	2	4
$T_{0,2}^{(1)}$ [μs]	2.20	2.25
$T_{0,2}^{(2)}$ [μs]	2.00	2.05
λ_0	0	1
λ_1	0	1
λ_2	0	1
η	$0.9(1-f_{\mathrm{DOSY}})$	$1.1(1-f_{\mathrm{DOSY}})$

Table S2 Gaussian fit parameters (mean distances $\langle r \rangle$ and standard deviations σ) of the DEER measurements on sections of FUS NTD. The lower and upper bounds of the bootstrapped 95% confidence intervals are given in brackets next to the fitted values. The fit parameters of the denatured, biphasic dispersed, and biphasic condensed conditions were employed for ensemble modelling. Data for double mutants A10C S29C and A105C G128C have been taken from [2].

		Dispersed fraction		Condensed fraction	
Mutant	Condition	$\langle r \rangle$ [Å]	σ [Å]	$\langle r \rangle$ [Å]	σ [Å]
A10C S29C	Denatured	36.7 [36.4, 36.9]	9.4 [9.1, 9.8]	-	-
	Monophasic	31.8 [31.5, 32.1]	9.9 [9.6, 10.4]	-	-
	Biphasic	32.1 [31.5, 32.1]	10.4 [9.6, 10.4]	28.2 [28.0, 29.3]	8.3 [8.0, 9.3]
	Bulk	-	-	29.7 [29.7, 29.7]	10.9 [10.9, 10.9]
S29C S61C	Denatured	47.9 [47.3, 48.4]	15.6 [14.9, 16.3]	-	-
	Monophasic	39.8 [39.6, 40.0]	14.2 [13.9, 14.6]	-	-
	Biphasic	40.0 [39.6, 40.0]	13.9 [13.9, 14.6]	38.3 [37.3, 39.0]	12.1 [11.0, 13.5]
S61C S86C	Denatured	40.2 [39.9, 40.6]	12.3 [11.9, 12.7]	-	-
	Monophasic	35.0 [34.8, 35.2]	11.1 [10.9, 11.3]	-	-
	Biphasic	35.2 [34.8, 35.2]	11.3 [10.9, 11.3]	31.9 [31.5, 32.7]	10.1 [9.7, 11.2]
S86C A105C	Denatured	35.5 [35.0, 35.9]	10.1 [9.7, 10.6]	-	-
	Monophasic	31.2 [31.1, 31.3]	9.8 [9.7, 10.0]	-	-
	Biphasic	31.3 [31.1, 31.3]	10.0 [9.7, 10.0]	30.0 [29.8, 30.5]	8.8 [8.5, 9.6]
A105C G128C	Denatured	39.7 [39.5, 39.9]	11.2 [10.9, 11.5]	-	-
	Monophasic	35.9 [35.7, 36.0]	11.5 [11.2, 11.7]	-	-
	Biphasic	36.0 [35.7, 36.0]	11.2 [11.2, 11.7]	33.4 [32.6, 33.9]	10.1 [9.2, 10.9]
	Bulk	-	-	31.6 [31.6, 31.6]	11.7 [11.7, 11.7]
G128C Q158C	Denatured	50.6 [50.3, 50.9]	15.6 [15.3, 16.0]	-	-
	Monophasic	43.9 [43.6, 44.2]	14.8 [14.4, 15.3]	-	-
	Biphasic	44.2 [43.6, 44.2]	14.7 [14.4, 15.3]	38.0 [37.4, 39.8]	11.5 [11.0, 12.6]
Q158C M184C	Denatured	40.7 [40.5, 40.9]	12.6 [12.4, 12.9]	-	-
	Monophasic	36.5 [36.4, 36.6]	12.2 [12.0, 12.4]	-	-
	Biphasic	36.6 [36.4, 36.6]	12.0 [12.0, 12.4]	35.4 [35.0, 35.8]	10.5 [9.7, 11.2]
M184C S205C	Denatured	36.4 [35.8, 36.9]	9.9 [9.4, 10.5]	-	-
	Monophasic	32.9 [32.8, 33.1]	10.2 [10.1, 10.4]	-	-
	Biphasic	33.1 [32.8, 33.1]	10.4 [10.1, 10.4]	31.3 [31.1, 31.9]	8.9 [8.7, 9.7]
S205C S236C	Denatured	44.2 [43.6, 44.8]	13.4 [13.0, 14.0]	-	-
	Monophasic	37.0 [36.8, 37.2]	12.6 [12.2, 13.0]	-	-
	Biphasic	37.2 [36.8, 37.2]	12.2 [12.2, 13.0]	34.7 [34.0, 35.4]	10.3 [9.5, 11.6]
S236C G267C	Denatured	43.8 [43.4, 44.1]	15.1 [14.7, 15.5]	-	-
	Monophasic	38.1 [37.8, 38.5]	12.9 [12.4, 13.5]	-	-
	Biphasic	38.5 [37.8, 38.5]	13.2 [12.4, 13.5]	33.0 [32.0, 33.7]	11.1 [10.3, 12.2]

Table S3 Reproducibility of Gaussian fit parameters (mean distances $\langle r \rangle$ and standard deviations σ) of the DEER measurements on selected sections of FUS NTD. The selected mutants were measured twice; S1 as shown in Table S2 and employed for ensemble modelling, and the reproduced sample S2.

			Dispersed fraction		Condensed fraction	
Mutant	Condition	Sample	$\langle r \rangle$ [Å]	σ [Å]	$\langle r \rangle$ [Å]	σ [Å]
G128C Q158C	Denatured	S1	50.6 [50.3, 50.9]	15.6 [15.3, 16.0]	-	-
		S2	50.4 [50.1, 50.6]	15.9 [15.5, 16.2]	-	-
	Monophasic	S1	43.9 [43.6, 44.2]	14.8 [14.4, 15.3]	-	-
		S2	42.7 [42.4, 43.0]	14.8 [14.4, 15.2]	-	-
	Biphasic	S1	44.2 [43.6, 44.2]	14.7 [14.4, 15.3]	38.0 [37.4, 39.8]	11.5 [11.0, 12.6]
		S2	43.0 [42.4, 43.0]	14.6 [14.4, 15.2]	40.6 [38.8, 41.7]	12.4 [11.4, 14.1]
S205C S236C	Denatured	S1	44.2 [43.6, 44.8]	13.4 [13.0, 14.0]	-	-
		S2	43.8 [42.8, 44.6]	14.3 [13.5, 15.3]	-	-
	Monophasic	S1	37.0 [36.8, 37.2]	12.6 [12.2, 13.0]	-	-
		S2	38.8 [38.7, 39.0]	13.2 [12.9, 13.5]	-	-
	Biphasic	S1	37.2 [36.8, 37.2]	12.2 [12.2, 13.0]	34.7 [34.0, 35.4]	10.3 [9.5, 11.6]
		S2	39.0 [38.7, 39.0]	12.9 [12.9, 13.5]	35.7 [34.8, 36.3]	10.2 [9.2, 11.2]

Supporting References

1 J. P. Gallivan and D. A. Dougherty, *Proc. Natl. Acad. Sci. U.S.A.*, 1999, **96**, 9459–9464.

2 L. Emmanouilidis, L. Esteban-Hofer, F. F. Damberger, T. de Vries, C. K. X. Nguyen, L. F. Ibáñez, S. Mergenthal, E. Klotzsch, M. Yulikov, G. Jeschke and F. H.-T. Allain, *Nat. Chem. Biol.*, 2021, **17**, 608–614.

3 K. Yokosawa, S. Kajimoto, D. Shibata, K. Kuroi, T. Konno and T. Nakabayashi, *J. Phys. Chem. Lett.*, 2022, **13**, 5692–5697.

4 J. J. Virtanen, L. Makowski, T. R. Sosnick and K. F. Freed, *Biophys. J.*, 2010, **99**, 1611–1619.

5 P. Huang, H. Xing, X. Zou, Q. Han, K. Liu, X. Sun, J. Wu and J. Fan, *Front. Mol. Biosci.*, 2021, **8**, 756075.
